# Genomic homeostasis is dysregulated in favour of apoptosis in the colonic epithelium of the azoxymethane treated rat

**DOI:** 10.1186/1472-6793-13-2

**Published:** 2013-01-23

**Authors:** Caroline A Kerr, Barney M Hines, Janet M Shaw, Robert Dunne, Lauren M Bragg, Julie Clarke, Trevor Lockett, Richard Head

**Affiliations:** 1 CSIRO Preventative Health Flagship, CSIRO, North Ryde, NSW 2113, Australia; 2 CSIRO, Food and Nutritional Sciences, North Ryde, NSW 2113, Australia; 3 CSIRO Division of Livestock Industries, Queensland Biosciences Precinct, St Lucia, Queensland 4067, Australia; 4 CSIRO, Mathematical and Information Sciences, North Ryde, New South Wales 1670, Australia; 5 CSIRO Food and Nutritional Sciences, Adelaide 5000, South Australia; 6 Graduate School of Medicine, University of Wollongong, Wollongong, NSW, Australia

**Keywords:** Colorectal cancer, Azoxymethane, Rats, Gene expression

## Abstract

**Background:**

The acute response to genotoxic carcinogens in rats is an important model for researching cancer initiation events. In this report we define the normal rat colonic epithelium by describing transcriptional events along the anterior-posterior axis and then investigate the acute effects of azoxymethane (AOM) on gene expression, with a particular emphasis on pathways associated with the maintenance of genomic integrity in the proximal and distal compartments using whole genome expression microarrays.

**Results:**

There are large transcriptional changes that occur in epithelial gene expression along the anterior-posterior axis of the normal healthy rat colon. AOM administration superimposes substantial changes on these basal gene expression patterns in both the distal and proximal rat colonic epithelium. In particular, the pathways associated with cell cycle and DNA damage and repair processes appear to be disrupted in favour of apoptosis.

**Conclusions:**

The healthy rats’ colon exhibits extensive gene expression changes between its proximal and distal ends. The most common changes are associated with metabolism, but more subtle expression changes in genes involved in genomic homeostasis are also evident. These latter changes presumably protect and maintain a healthy colonic epithelium against incidental dietary and environmental insults. AOM induces substantial changes in gene expression, resulting in an early switch in the cell cycle process, involving p53 signalling, towards cell cycle arrest leading to the more effective process of apoptosis to counteract this genotoxic insult.

## Background

Colorectal cancer (CRC) is the third most common cancer in males and second most common in females world-wide [1]. The majority of these cancers are considered preventable by appropriate diet and associated lifestyle factors [2]. Dietary patterns consisting of micronutrient dense, low-fat, high-fibre food patterns protect against colorectal cancer [3,4]. Conversely, specific sources of dietary protein have been linked to increased CRC risk [5] and animal studies have indicated that different dietary proteins can induce DNA damage in the rats’ colon [6]. Consequently, the challenge is to translate this information into strategies that prevent CRC. One of the first steps to doing this is to understand the early molecular events involved in oncogenesis and develop hypotheses on the role played by environmental factors such as diet in this process.

The azoxymethane (AOM)-treated rodent provides an important tool in the study of sporadic CRC development and progression [7]. It has been used extensively to study colon carcinogenesis and its prevention, in at least two formats that model different aspects of CRC [8,9]. One version of this model studies tumour development (at least 14 weeks post-treatment) to find the underlying signalling pathways of colon carcinogenesis. For instance, it has been used to investigate mouse models of colorectal carcinogenesis using gene expression profiling and has provided significant insights into the role of reactivated embryonic signatures in colon tumours [10]. The other main version of the AOM model is the ‘cancer initiation’ model, which is used to study the early response to the carcinogen, where tissues are harvested shortly after treatment (around 0–48 hours) [11]. Using this latter acute AOM model, we report here some of the early transcriptional events induced by this carcinogen in mucosal tissue along the length of the colon in rats.

## Results and discussion

The colonic epithelium is one of the largest epithelial barriers in the body and is in a constant state of self-renewal. In order to understand the effects of a carcinogenic insult to this tissue, it is important to develop an understanding of the natural morphologic and molecular features of the normal rat colon. It has been demonstrated that rat colonic stem cells are located in different positions and behave differently in crypts sampled from different points along the anterior-posterior length of the colon [12]. In distal sections, stem cells are located in the crypt base from whence progeny differentiating cells then migrate up towards the lumen, ultimately undergoing anoikis and sloughing off into the digesta [12]. In proximal sections, stem cells are located in the middle one-third of the crypt. Differentiating cells migrate bi-directionally from this source with some differentiating colonocytes migrating towards the lumen, while others migrate into the crypt base [12]. Our own data confirm the observations of others that the crypt height in the normal rat distal colon is greater than that for the proximal colon (34.4 ± 0.26 and 27.4 ± 0.27 cells respectively, P<0.0001, n=10). Despite these morphological differences, no significant differences in rates of baseline apoptosis between the proximal and distal normal (saline treated) colon (0.018 ± 0.012 and 0.057 ± 0.028 cells per crypt, respectively) were observed.

**Figure 1:**
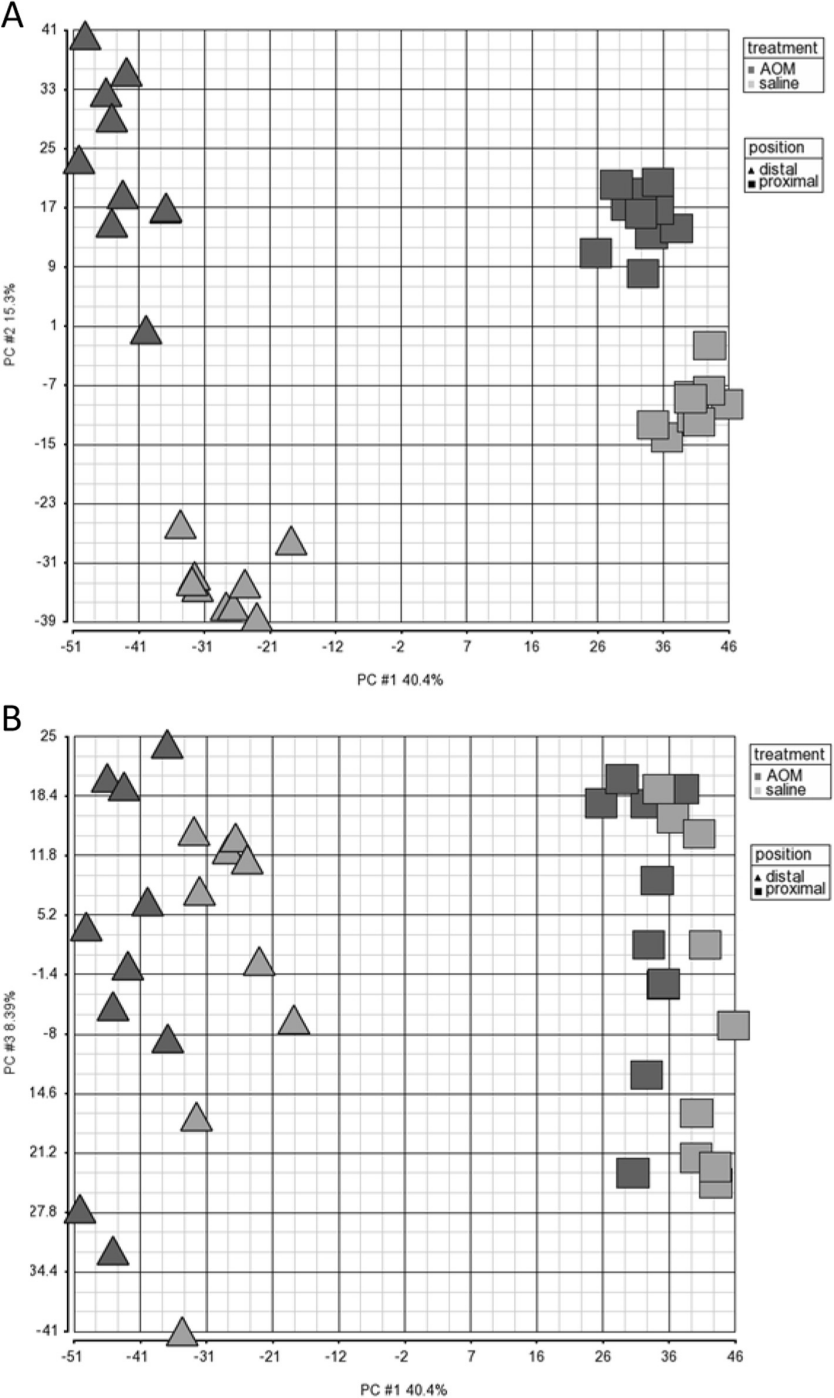
**Global gene expression depicted as a principal component analyses (PCA, covariate) showing the A) PC1 and PC2 and B) PC1 and PC3, illustrating the effect of six hours post-AOM on proximal and distal sections of the rat colon (AOM distal**▲**, AOM proximal **■**, saline distal “light gray triangle symbol” and saline proximal “light gray square symbol”).**

It has been shown in mice that significant numbers of genes are differentially expressed along functionally distinct regions of the gastrointestinal tract [13,14] and this is also true for the normal human colon [15,16]. When gene expression in the normal colon of the rat was examined at the level of individual genes, the proximal expression profile differed markedly from that of the distal colonic epithelium with 4527 genes differentially expressed (False Discovery Rate (FDR) 0.05) (Figure 1). These genes are listed in Additional file 1: Table S1. This microarray dataset was also validated by demonstrating that the top 8 genes most differentially expressed between the proximal and distal colon were also 100% consistently differentially expressed using real time RTPCR (as shown in Additional file 1: Table S3).

**Figure 2:**
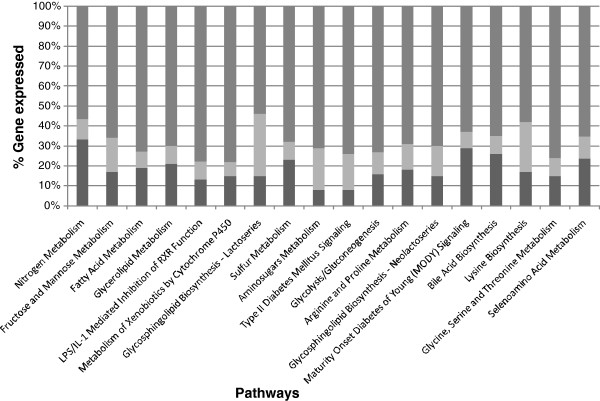
**The top 20 pathways differentially expressed along the length of the normal healthy rat colon (proximal compared to distal) depicted as up (**□**), down (**■**) or no overlap with the dataset (i.e. genes in the canonical pathway but not differentially expressed) (gray square symbol).**

When the functional groupings of these genes were considered through pathway analysis, most of the top 20 pathways identified were broadly associated with intestinal metabolism functions (see Figure 2 for the top 20 pathways). The magnitude of these changes in expression can be very large (changes up to 104 fold). We consider this association with metabolism most likely reflects the changing profile of digestive functions naturally occurring along the length of the colon. Consequently, these position-associated profiles provide the background against which changes in gene expression induced by colonic carcinogens need to be assessed.

The carcinogens AOM and 1,2-Dimethylhydrazine are metabolised by cytochrome P450 (CYP2E1) into methylazoxymethanol. In turn this breaks down to form highly reactive alkylating species which can lead to the addition of methyl adducts at the O^6^ position of Guanine residues in the DNA to form the promutagenic modified base O^6^ methyl guanine (O6-mdGua). If this modified base is not repaired, it can lead to G:C to A:T transition mutations during replication Tan [17,18]. These DNA adduct-induced mutations are found commonly in colorectal cancers [18]. So not surprisingly, AOM induces substantial transcriptional changes in the mucosa of the rat colon six hours after subcutaneous administration (Figure 1). The genes differentially expressed in response to AOM are listed in Additional file 1: Table S1. There were 1960 and 9441 genes differently expressed (FDR 0.05) in the proximal and distal colons respectively of AOM-treated rats when compared with the same tissues from normal (saline treated) animals. The fold changes were up to 6.6 in the proximal and 10.7 in the distal colon.

At a whole genome level, principal component analysis (PCA) revealed that the magnitude of the site effect on gene expression (proximal versus distal colon) was equal to or greater than that of AOM for the two highest principal components (PCs) (Figure 1A). Further examination of the PCA revealed that PC1 and PC3 best explained the effect of AOM (Figure 1B), and PC1 and PC4 best explained the effect of ‘site’ (not shown). As it has been previously shown that the greatest effects of AOM in the rat, in terms of tumours numbers are exhibited in the distal colon [10] and human tumours predominately occur in the most distal colonic region, i.e. sigmoid colon and the rectum [16], it is not surprising that there almost 10-fold more genes expressed in the distal rat colon at 6 hours post treatment. As a consequence, this report will concentrate predominantly on the effects this carcinogen in this colonic region with a particular focus on DNA damage and repair.

In a previous study using the “cancer initiation” AOM model in Sprague Dawley rats, Tan et al. measured levels of O6-mdGua accumulating in the DNA from a number of tissues harvested 6 hours and 48 hours after subcutaneous injection of this carcinogen. They observed that 6 hours after AOM exposure, the highest levels O6-mdGua occurred in the following tissues (in order of highest to lowest): liver, distal colon, proximal colon, proximal small intestine (SI), and kidney. The stomach, distal SI, bladder, spleen, blood and lung had relatively low levels O6-mdGua. While levels of this highly mutagenic alkylation product had dropped in most tissues tested by 48 h post AOM administration, O6-mdGua levels remained high at this time point in the proximal and distal colon, kidney and bladder. This is a significant finding as the distal colon is more prone to AOM induced tumours than any other tissue [17] and tumours in the bladder and kidney have been observed in animals treated with high levels of dimethyl hydrazine, a precursor of AOM [19].

**Figure 3:**
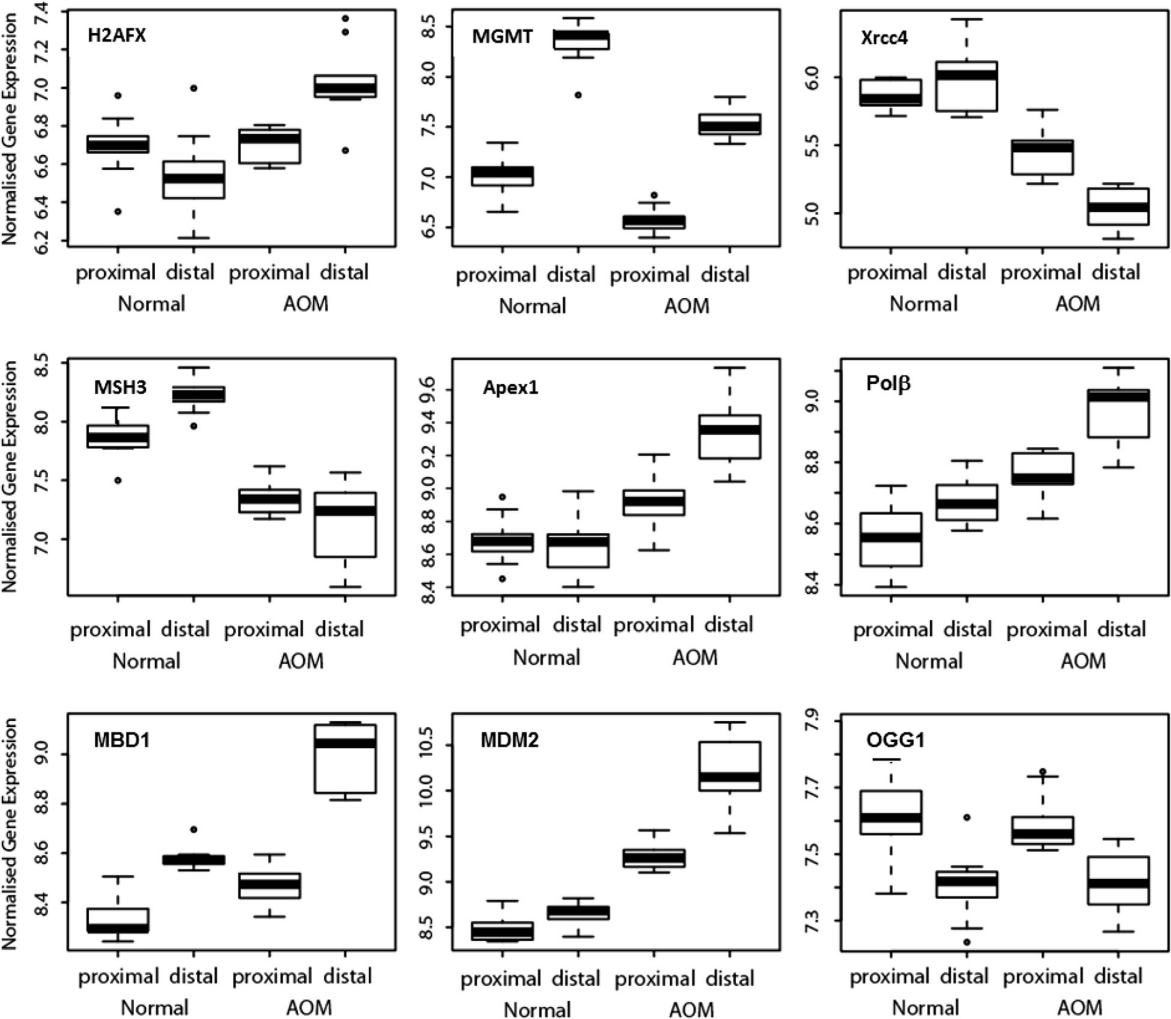
**Box plots demonstrating gene expression (microarray dataset) six hours after AOM treatment in the proximal and distal colonic epithelium for the genes: H2AFX (H2A histone family member X), MGMT (O-6-methylguanine DNA methyltransferase), Xrcc4 (X-ray repair complementing defective repair in Chinese hamster cells 4), MSH3 (mutS homolog 3 (E. coli)), Apex1 (apurinic/apyrimidinic endonuclease 1), Pol β (polymerase, DNA directed beta), MBD1 (methyl-CpG binding domain protein 1), MDM2 (p53 E3 ubiquitin protein ligase homolog), OGG1** (**8-oxoguanine DNA glycosylase).**

A key enzyme involved in the repair of O6-mdGua is O-6-methylguanine DNA methyltransferase (MGMT). In a ‘suicide’ reaction the methyl adduct from one modified guanine base is transferred to a cysteine residue in the active site of one molecule of enzyme resulting in the inactivation of that molecule of enzyme and earmarking it for ubiquitination and degradation [20]. Interestingly, in the current study, in the normal colon, the level of expression of MGMT was greater in the distal section compared to the proximal section (Figure 3). This would be consistent with an adaptation to a higher basal metabolic demand for DNA adduct repair in the distal colonic mucosa relative to the proximal. This could arise in response to dietary mutagens in the colonic digesta becoming more concentrated as more and more water is removed during its transit from proximal to distal colon. Whatever the drivers may be, however, this change in MGMT levels from proximal to distal colon is likely to form a part of an innate homeostatic process to maintain genomic integrity in a healthy colonic mucosa.

Six hours after the administration of AOM, MGMT expression was down-regulated in both the proximal and distal colonic epithelium (fold changes −1.39 and −1.79 respectively). As there are high levels of O6-mdGua present in the DNA of the distal colon at this time [17] and with MGMT being the primary enzyme for repair of DNA methyl adducts, it appears that MGMT is rapidly depleted instead of being up-regulated in response to AOM. As the animals survive AOM challenge well with no apparent significant loss of colonic function, this observation suggests that other repair mechanisms are brought into play to ensure the rapid return to normal colonic function.

Further analysis revealed that the expression of a number of other DNA repair and damage genes was also altered in response to AOM, particularly in the distal colon (see Figure 3 and Additional file 1: Table S2). Expression of the damaged DNA binding and sensing H2A histone family member X (H2AFX) gene was significantly up-regulated in response to AOM (p=3.08E-08, fold change 1.5) (Figure 3), confirming that repair mechanisms other than MGMT are deployed in response to the AOM perturbation. In terms of single strand break repair, there are a number of nucleotide-excision repair (NER) (n=16) genes differentially expressed in the distal colon in response to AOM treatment and 80% of them were up-regulated. This is important as NER is the most flexible of the DNA repair pathways as it repairs bulky DNA lesions [21]. Other base-excision repair associated genes also showing increased expression in response to treatment with AOM include Apex1 (apurinic/apyrimidinic endonuclease 1) had a 1.6 fold change and Polβ, (polymerase, DNA directed beta) a 1.4 fold change (see Figures 3). The mismatch repair (MMR) pathway is an important pathway involved in the DNA damage response to carcinogen induced lesions resulting in cell cycle arrest and, at high lesion load, apoptosis [22]. However, AOM treatment led to the down-regulated response of MMR genes (n=4). For instance, MSH3 (mutS homolog 3 (E. coli)), which recognises insertion/deletion mismatches containing two or more extra bases [23] showed decreased expression (−2.1 fold change) with AOM (Figure 3). These observations suggest that the MMR pathway in general may be down-regulated in response to AOM and are consistent with AOM’s major mode of action involving DNA adduct formation and induction of point mutations rather than the formation of multi-base mismatches.

Double-strand breaks (DSB), in which both strands in the DNA double helix are severed, are particularly hazardous to the cell because they can lead to genome rearrangements [24]. DSB repair via homologous recombination (HR) is an important process as it takes place late in the S- and G2-phases of the cell cycle to prevent unrepaired double strand breaks from causing down-stream problems in transcription, replication and chromosome segregation [25]. In the distal colon there were nine genes from this pathway up-regulated in response to AOM. For instance, Xrcc2, which plays a central role in this pathway and encodes a member of the Rad51 family of proteins, was up-regulated 1.5-fold. Conversely, the DSB repair via non-homologous end-joining (NHEJ) pathway was down-regulated with AOM, demonstrated by the decreased expression of Xrcc4 (X-ray repair complementing defective repair in Chinese hamster cells 4, -1.94 fold change) (Figure 3). Consequently, there is some evidence that single and double strand break repair functions may be compromised in response to AOM treatment. These data coupled with the accumulation of unrepaired O6-mdGua lesions in colonic epithelium in response to carcinogen, indicates that at six hours post treatment other cellular processes such as cell cycle arrest and apoptosis becomes more important in maintaining mucosal integrity in response to this genomic insult.

**Figure 4:**
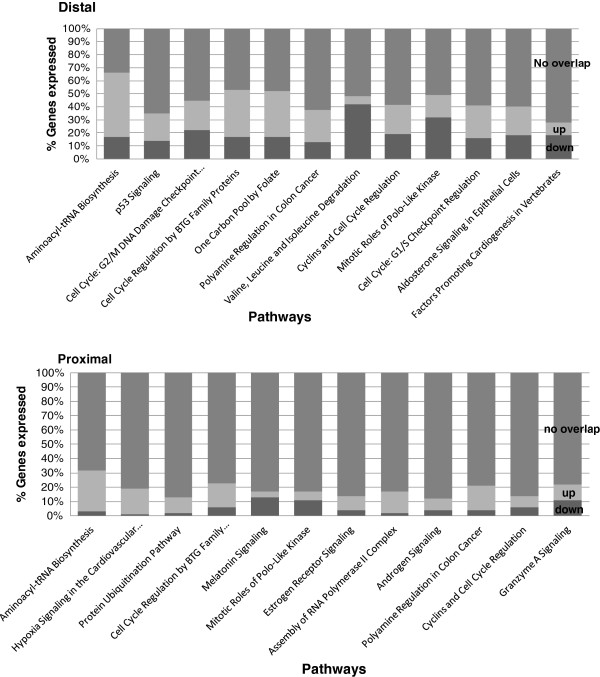
**The top 12 pathways differentially expressed in response to AOM in the distal and proximal sections.** Depicted as up □), down (■) or no overlap with the dataset (i.e. genes in the canonical pathway but not differentially expressed) (gray square symbol).

To investigate the biological consequences of unrepaired DNA damage, such as the O6-mdGua lesions, the functionality of the top 800 genes differentially expressed in response to AOM in the proximal and distal colon, was examined through pathways analysis. The top 12 pathways in which the highest percentage of component genes displayed AOM-associated differential expression relative to the saline treated control in the distal and proximal colonic mucosa are shown in Figure 4. There was a number of cell cycle regulation pathways differentially expressed in the distal colon 6 hours after the treatment with AOM. These included, “p53 signalling” and “Cell cycle regulation by B cell translocation (BTG)” (see Figure 4 for more cell cycle pathways). This is noteworthy as deregulated cell cycle processes are a prominent feature of oncogenesis [25]. Overall, the response suggests a trend towards cell cycle arrest in response to AOM.

**Figure 5:**
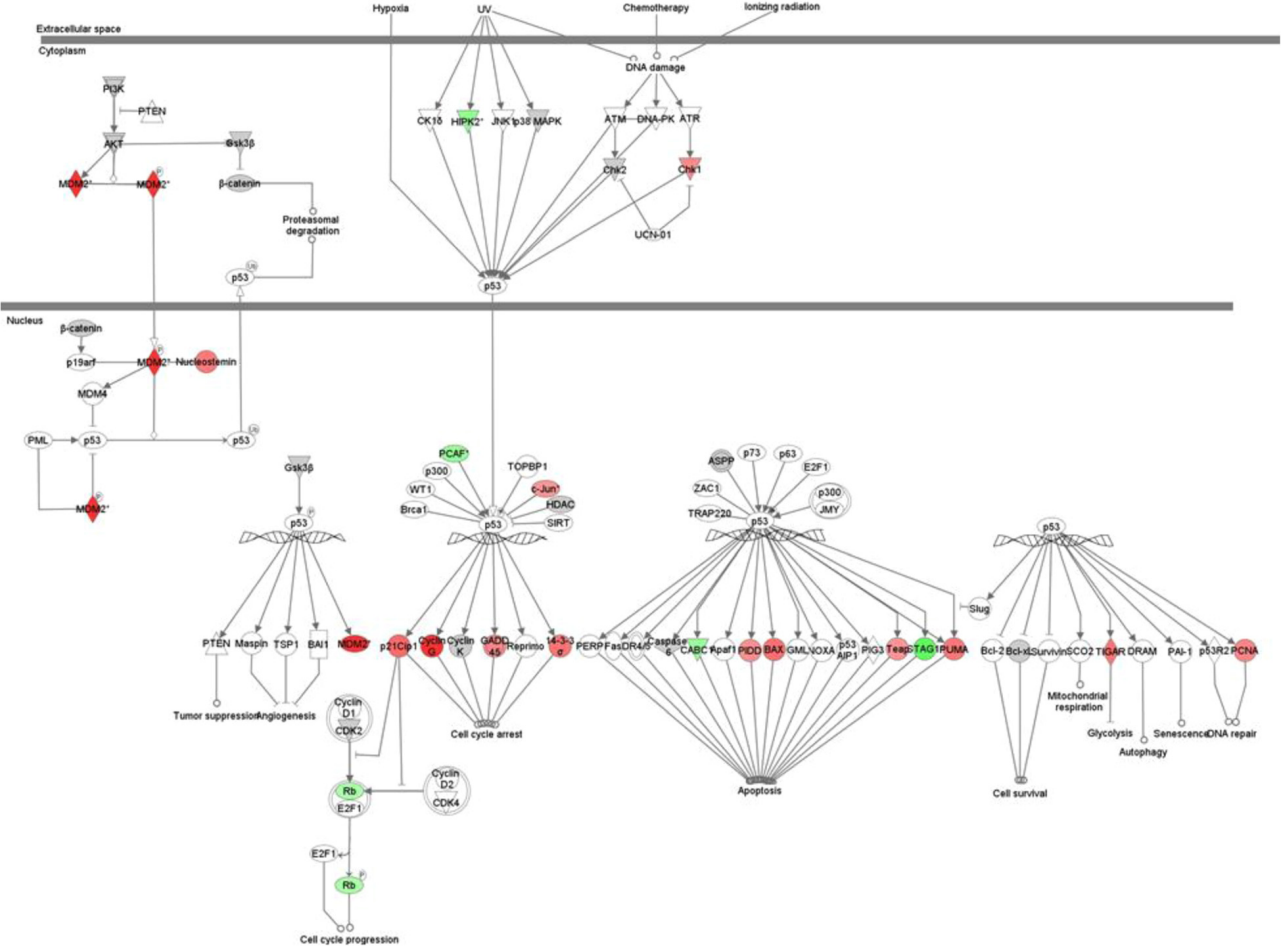
**p53 signalling pathway in the distal colon in response to AOM “red circle symbol” up-regulated and “green circle symbol” down-regulated).** Details of the expressed genes can be found in Additional file 1: TableS4.

DNA damage checkpoint control mechanisms tightly regulate progression through the cell cycle, ensuring the fidelity of cell division which is an important self-defence mechanism for the maintenance of genome stability [26]. A number of observations support the involvement of the p53 signalling (see Figure 5) and BTG pathways in AOM-induced cell cycle arrest in the distal colon: Cyclin G1, a protein involved in G2/M phase arrest and regulates p53 [27] expression increased 4.34 fold; cyclin dependent kinase inhibitor 1 (p21Cip1), which inhibits the activity of cyclin-CDK2 or -CDK4 complexes [28], is marginally up-regulated, and as a result Retinoblastoma 1 (Rb1) is down-regulated (1.5 fold); BTG family member 2 (BTG2), an important transcriptional regulator that impairs G1-S cell cycle progression [29] increased 2.34 fold and MDM2 (p53 binding protein homolog, 2.81 fold change increase) (Figure 3) which is also a member of another expressed pathway, “Cell Cycle: G2/M DNA Damage Checkpoint Regulation” (p= 0.002). This latter cell cycle pathway has other genes differentially expressed. For example, Chek1, which is a checkpoint regulator of cell cycle arrest and putative tumour suppressor in response to DNA damage [30], is up-regulated (1.6 fold change). Most cancer cells harbour mutations in tumour suppressors and/or oncogenes which would normally control cell cycle checkpoints [26]. Therefore, cell cycle regulation is important in the maintenance of genomic stability and to prevent cells that have undergone malignant transformation progressing through the cell cycle phases.

**Figure 6:**
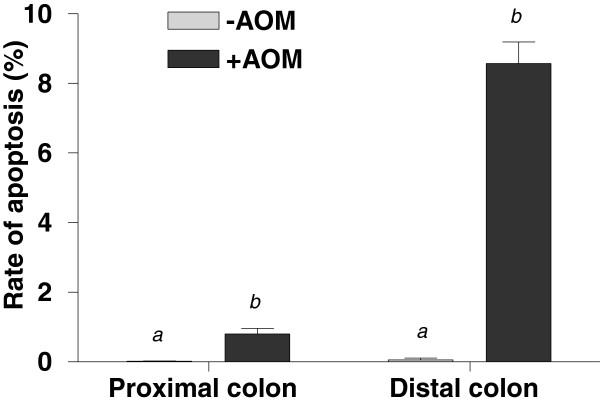
**Apoptotic indices (% apoptotic cells per crypt height) of the proximal and distal colonic epithelium of rats 6 h after injection with saline (−AOM) and AOM (+AOM) as determined by morphological assessment of the colonic mucosa.** Data are mean±SEM (n=20). Means within the same colonic section with different superscripts are significantly different (P<0.0001).

The p53 signaling pathway (pvalue 1.66×10^-4^) is significantly expressed (the second most highly expressed pathway in the distal colon) in response to AOM. Loss of p53 function is thought to be a contributing factor in colorectal cancer, because the p53-dependent pathway shuts down damaged cells, either through apoptosis, cell-cycle arrest or cellular senescence [31]. Apoptosis was observed histologically to have significantly increased at six hours after the AOM treatment (Figure 6). As MGMT is depleted at this time point, we hypothesise that the main cellular response to AOM involves the early depletion of MGMT then a switch to the induction of apoptosis and this most likely first occurs through the p53 signalling pathway. This is demonstrated through closer examination of the p53 signalling pathway response to AOM in the distal colon (see Figure 5). Firstly, genes such as TP53INP1 (tumour protein p53 inducible nuclear protein 1), which is a key transcriptional regulator that responds to a variety of cellular stresses, including DNA damage, oxidative stress and activated oncogenes, to regulate key cellular processes including the induction of apoptosis [32], is up-regulated 1.5 fold. Furthermore, Caspases 1 (Casp1) and Casp3 are up-regulated in response to AOM in the distal colon (1.2 fold change for both). The activation of Casp3 triggers an execution arm of the apoptosis response initiating DNA fragmentation [33]. The apoptotic function of caspases is regulated by the Bcl-2 family of proteins [34]. Accordingly, in response to AOM, Bcl-associated X protein (Bax) which is critically important in the up-regulation of apoptosis, is increased two fold. Furthermore, there is also decreased expression of the caspase-activated inhibitor Avon by 1.2-fold. Therefore, these results indicate that at 6 hours after being treated with AOM, one of the major effects of this carcinogen occurs through the p53 signalling pathway and the result is cell cycle arrest and a cellular switch towards apoptosis.

**Figure 7:**
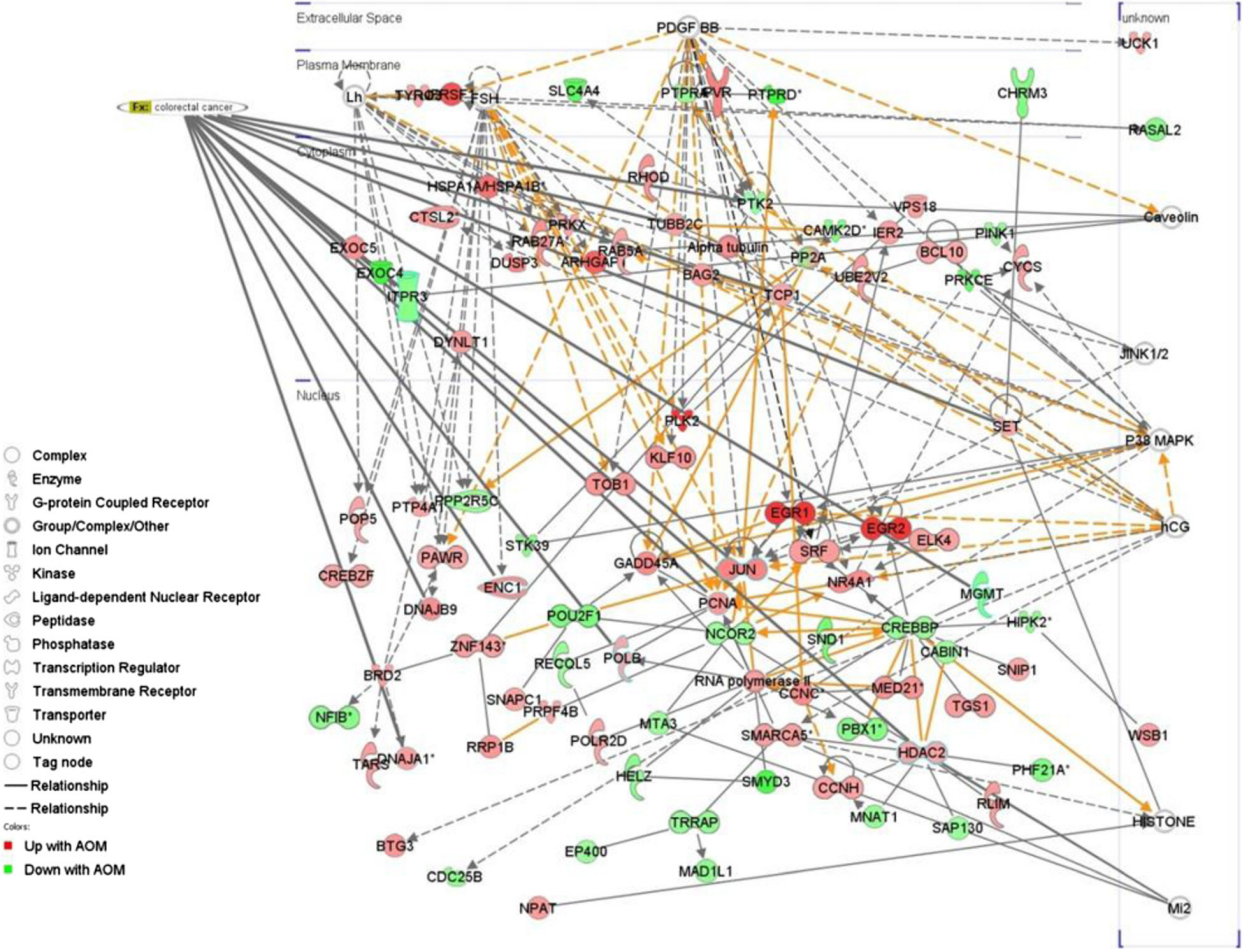
**Network expression in the proximal rat colon 6 hours after the administration of AOM.** The top 800 differentially expressed genes were networked and the top three networks were merged. Genes relevant to two cancer pathways and individual cancer related genes are highlighted.

**Figure 8:**
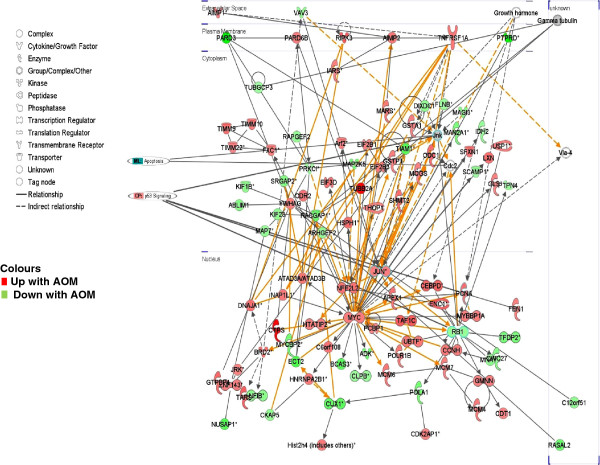
**Network expression in the distal rat colon 6 hours after the administration of AOM.** The top 800 differentially expressed genes were networked (Ingenuity®) and the top three networks were merged. Genes relevant to two cancer pathways and individual cancer related genes are highlighted.

Gene network analysis was used to further understand the function of genes expressed in response to AOM in each colonic segment, in particular the early induction of apoptosis. When the top three AOM/proximal networks were merged (Figure 7) there were 13 genes with functional annotations (p= 7.16×10^-4^) associated with colorectal cancer including the up-regulation (1.6-fold) of transcriptional regulator c-JUN (jun proto-oncogene) and the down-regulation of MGMT (1.4-fold). In terms of the AOM/distal network there were eight genes associated with colon cancer (p= 5.04×10^-4^), including the transcriptional regulator MYC (up-regulated 1.6 -fold) and the previously mentioned c-JUN (Figure 8). When the p53 signalling pathway and apoptosis genes were cross-referenced in this network, c-Jun Kinase (JNK), which regulates cJUN and is an important regulator of cell death [35], was a gene common to both and is linked through the gene network to the down-regulated p53 signalling transcriptional regulator, Retinoblastoma 1 (Rb1). Taken together these results suggest that the early genomic damage effects of AOM on the colonic mucosa may be mediated through the p53 pathway, favouring apoptosis through c-JUN/JNK signalling and preventing cell cycle progression through reduced Rb1 expression. Whilst this mechanism is hypothetical, it provides a framework for the further elucidation of the key mechanisms underpinning the cellular switch towards apoptosis in the gut mucosa in response to alkylating carcinogen challenge.

## Conclusion

The healthy rat colonic mucosa exhibits extensive gene expression changes from its proximal to distal end reflecting regional changes in metabolic function. The normal rat colon also has naturally occurring protective and genomic repair mechanisms expressed dynamically, albeit subtly, along the proximal/distal axis. Six hours after administration of AOM, substantial changes in gene expression have occurred in the colonic mucosa and these also differ along the length of the colon. The changes are greater in the distal colon and appear particularly associated with the sensing of genomic damage, associated cell cycle arrest and a cellular switch towards the induction of apoptosis. Consequently, the genomic homeostasis mechanisms that naturally exist to combat dietary and environmental insults in the colon of the normal rat appear to be dysregulated by AOM resulting in a cellular switch through p53 signaling to more efficient genes associated with the apoptotic response, a genetic response that is also reflected histologically.

## Methods

### Animals and diets

Forty male Sprague Dawley rats weighing approximately 176 ± 2.4 g were purchased from the Animal Resource Centre, Western Australia. They were housed in wire-bottomed caging in a temperature controlled room (22-24°C) with a 12 h light/dark cycle. They were randomly allocated into two groups (n=20) with approximately equal body weights. They were given free access to water and a modified AIN-93G diet [36]. Both groups were fed this diet for 28 days. One group was then injected subcutaneously with azoxymethane (AOM; 15 mg/kg; Sigma Chemical Co., St. Louis, MO, USA) the other with saline. Six hours after injection the rats were anaesthetised with isoflurane and killed by exsanguination. The large bowel (excluding the rectum) was removed, opened longitudinally along the mesenteric border and digesta removed. The colon was rinsed clean with PBS and transferred to a chilled ceramic plate for dissection. The colons were on average 15 ± 0.5 cm long. The last 0.5 cm distal and first 0.5 cm proximal sections of the colon were discarded and the next 2 cm from both ends placed into 10% buffered formalin (Sigma) for morphological assessment of apoptosis. Mucosal samples for gene expression and protein analyses were collected by scraping the next 4 cm of proximal and distal colon with new microscope slides. The mucosal samples were placed in RNA*later* (Sigma Chemical Co., St. Louis, MO, USA) and then stored at −80°C for later processing. All instruments were replaced or cleaned thoroughly between animals.

All procedures involving animals were approved by the Commonwealth Scientific and Industrial Research Organisation (CSIRO) Human Nutrition Animal Ethics Committee and complied with the *Australian code of practice (2004).* [ http://www.nhmrc.gov.au/publications/synopses/ea16syn.htm].

### Measurement of crypt height and colonocyte apoptosis

The rate of apoptosis was determined on paraffin-embedded sections (4 μm) stained with haematoxylin (Harris’, BDH Laboratory Supplies, England). An Olympus BX-41 light microscope (Olympus Corp., Japan) was used to identify 20 randomly chosen intact crypts and to determine the crypt height by counting the total number of cells from the base to the lumen using a previously validated technique [37]. The number of apoptotic cells was identified by cell shrinkage, presence of condensed chromatin, and sharply delineated cell borders surrounded by a clear halo as described by [38]. All histological analyses were performed in a blinded fashion by a single operator. The rates of apoptosis for each section of colon (±AOM) were analysed with Mann Whitney t-tests using GraphPad Prism Version 4.00 (GraphPad Software Inc. San Diego, CA, USA). Data are expressed as mean ± standard error of the mean (SEM).

### Acquisition and data analysis

Proximal and distal sections from ten rats were used from each group. It was ascertained in a preliminary study that investigated baseline variation in this model and tissue type, that n=10 was a sufficient sample (see Gene Expression Omnibus (GEO) accession number GSE13802 for the complete dataset of this pilot study). The distal and proximal colonic mucosal samples from the AOM and saline treated rats were removed from the RNAlater stabilisation reagent (Sigma, Australia) and placed in 1ml of TRIzol® Reagent (Invitrogen, Sydney, N.S.W., Australia). Samples were then homogenised using beads (mix of 2.5 mm glass and 0.1 - 1.0 mm diameter silicon-zirconian beads) in a MiniBeadbeater-8™ (BioSpec Products Inc. Oklahoma, US). Total RNA was extracted according to the TRIzol® Reagent manufacturer’s instruction after which samples were further purified using RNAeasy mini spin columns (QIAGEN, Doncaster, Victoria, Australia) with a DNase on-column digestion as per the manufacturer’s instructions. The integrity of the RNA was checked using a Bioanalyzer 2100 (Agilent Technologies) and quantified using a NanoDrop® ND-1000 Spectrophotometer. Ten AOM rat and nine saline rat proximal and distal colonic epithelia (one saline set was dropped due to substandard RNA quality), i.e. 38 RNA (4.5 μg) samples, were processed for microarray expression analysis using high-density oligonucleotide arrays (Affymetrix® GeneChip array, Affymetrix®, Santa Clara, CA, USA) commensurate with the manufacturer’s instructions. The complete microarray dataset from this study can be sourced at NCBI’s Gene Expression Omnibus (GEO accession GSE15184).

Affymetrix® Gene Chip Rat Expression 230® results were analysed using the Partek® genomics suite software for differential expression, using an RMA normalization method. This software was used to Principal Component Analysis (PCA) which is a mathematical algorithm that reduces the dimensionality of the data by identifying directions, called principal components (e.g. PC1, PC2, etc.), along which the variation in the data is maximal [39]. The results were then plotted so that it is possible to visually assess similarities and differences between samples and determine whether samples can be grouped. The Partek software was also used to generate lists of differentially expressed genes by obtaining estimates of variance components for mixed models, using the method of moments estimation [40], restricted maximum likelihood estimation (REML) [41], and minimum variance quadratic unbiased estimation (MIVQUE) [42] using Analysis of Variance model that included rat number, colonic position (proximal or distal) and treatment (AOM or saline). As there is multiplicity of genes in microarray datasets, particularly for genes with small standard errors that can generate false discoveries, we used the False Discovery Rate (FDR) [43] to restrict our gene lists beyond p-values. The Gene Ontology Biological Processing and Molecular function terms [44] were added to the lists of differentially expressed. Individual gene data is presented using Box and Whisker plots which describes the dataset on an interval scale, i.e. as explanatory data analysis, to demonstrate the shape of the distribution, its central value, and its variability. The ends of the box are the upper and lower quartiles, so the box spans the interquartile range, the median is marked by a vertical line inside the box and the whiskers are the two lines outside the box that extend to the highest and lowest observations.

### Pathway and network expression

While the characterization of each gene that is differentially expressed in response to AOM as outlined above provides useful data, the identification of specific pathways that are changed in response to the AOM treatment is important for understanding the early changes that occur at a transcriptome level. To further understand the biology of gene expression comparisons, beyond the lists of expressed gene, pathway and network analysis was also performed using Ingenuity Pathway Analysis (Ingenuity® Systems, Inc., Redwood City, CA, USA, http://www.ingenuity.com), a curated knowledge base with over 1·5 million entries to determine the pathways that are perturbed by AOM. IPA identifies differentially expressed pathways based on the probability of having the observed number of differentially expressed genes associated with the dataset for that pathway in Ingenuity’s propriety database, by random chance and the p-value is calculated with the right-tailed Fisher’s Exact Test (Ingenuity® Systems, http://www.ingenuity.com). This analysis was applied to lists of the top 800 differentially expressed genes from comparisons of normal proximal rat colon to normal distal rat colon and the AOM-induced changes in both the proximal and distal colon. The gene network analysis was performed as described by [45] and Ingenuity® Systems, http://www.ingenuity.com.

### Real-time PCR validation

As there is a risk of false discovery associated with microarray experiments (see above) it is important to verify data using an independent technology platform such as RTPCR. As a result the top eight differentially expressed genes between proximal and distal rat colon that were identified by microarray data analysis were chosen to be a representative subset and were measured by qRTPCR using TaqMan® Universal PCR Master Mix commensurate with the manufacturer’s instructions). Reactions were performed in 20 ul reaction volumes using an ABI PRISM® 7700 Sequence Detection System. Data were normalised using the Relative Quantitation of Gene Expression method as outlined in the ABI 7700 manual. An aliquot of any given RNA sample used for microarray gene expression analysis was reverse-transcribed to provide the substrate for qRTPCR quantification.

## Competing interests

The authors declare that they have no competing interests.

## Authors’ contributions

CK co-designed the study with JC, co-authored the manuscript with RD, LMB, JC, TL and RH, lead the molecular work, guided the study concept and helped with data interpretation. BMH and JMS performed the molecular work, JC carried out the animal study and the tissue results, RD, JMS and LMB carried out the data analysis, TL and RH developed the concept of the study and sought funding. All authors read and approved the final manuscript.

## Supplementary Material

Additional file 1: Table S1 The complete listing of transcript contrasts between proximal and distal in the normal epithelium (saline); between saline and AOM epithelium in the proximal and between saline and AOM epithelium in the distal colon. **Table S2.** A) Genes with Gene Ontology Biological Processing term associated with DNA damage and repair in the AOM versus saline contrast in the proximal colon. B) Genes with Gene Ontology Biological Processing term associated with DNA damage and repair in the AOM versus saline contrast in the distal colon. **Table S3.** Summary of genes validated by real time PCR for microarray differential expression. Genes: UDP-GlcNAc: betaGal beta-1,3-N-acetylglucosaminyltransferase 7, solute carrier family 34 (sodium phosphate), member 2; similar to N-acetylglucosamine 6-O-sulfotransferase (predicted), peroxiredoxin 6, homeo box D10 (predicted), protein kinase, cAMP dependent, catalytic, beta; cytochrome P450, family 4, subfamily f, polypeptide 1 and cancer susceptibility candidate 4 (predicted). **Table S4:** Details of the genes expressed in the p53 signaling pathway. Click here for file
