# Supplementary material for: Genomic homeostasis is dysregulated in favour of apoptosis in the colonic epithelium of the azoxymethane treated rat
Source: BMC Physiol. 2013 Jan 23;13:2. doi: 10.1186/1472-6793-13-2 (PMC3561103; doi:10.1186/1472-6793-13-2)
Supplement: Additional file 1: Table S1 — The complete listing of transcript contrasts between proximal and distal in the normal epithelium (saline); between saline and AOM epithelium in the proximal and between saline and AOM epithelium in the distal colon. Table S2. A) Genes with Gene Ontology Biological Processing term associated with DNA damage and repair in the AOM versus saline contrast in the proximal colon. B) Genes with Gene Ontology Biological Processing term associated with DNA damage and repair in the AOM versus saline contrast in the distal colon. Table S3. Summary of genes validated by real time PCR for microarray differential expression. Genes: UDP-GlcNAc: betaGal beta-1,3-N-acetylglucosaminyltransferase 7, solute carrier family 34 (sodium phosphate), member 2; similar to N-acetylglucosamine 6-O-sulfotransferase (predicted), peroxiredoxin 6, homeo box D10 (predicted), protein kinase, cAMP dependent, catalytic, beta; cytochrome P450, family 4, subfamily f, polypeptide 1 and cancer susceptibility candidate 4 (predicted). Table S4: Details of the genes expressed in the p53 signaling pathway. [file 1472-6793-13-2-S1.pdf]

Table S1: Lists of transcript contrasts between proximal and distal in the normal epithelium (saline), between saline and AOM epithelium in the proximal and again distal colon.

SALINE: Distal versus Proximal				PROXIMAL: Saline versus AOM				DISTAL: Saline versus AOM			
ID	symbol	t	adj.P.Val	ID	symbol	t	adj.P.Val	Probe ID	symbol	t	adj.P.Val
1368467_at	"Cyp4f1"	" 127.9239"	"0.00000000"	1394564_at	NA	-18.2403	0.00000002	1367764_at	Ccng1	-16.623	0
1383878_at	"Hoxd10_predicted"	"-110.6893"	"0.00000000"	1374495_at	Lrba_predicted	17.7553	0.00000002	1374347_at	Cdkal1	23.3626	0
1385258_at	"B3gnt7"	" -77.7640"	"0.00000000"	1396763_at	LOC301455	17.0256	0.00000005	1368513_at	Enpep	16.9854	0
1368746_a_at	"Atp12a"	" -60.7514"	"0.00000000"	1368106_at	Plk2	-16.8478	0.00000006	1381445_at	Esrrg	18.5234	0
1387994_at	"Hsd17b6"	" 56.1454"	"0.00000000"	1394456_at	NA	16.7966	0.00000006	1370153_at	Gdf15	-26.584	0
1383014_at	NA	" 42.1280"	"0.00000000"	1383770_at	NA	16.9002	0.00000006	1383491_at	Isg20l1_pre	-17.742	0
1387254_at	"Ghrl"	" 40.7556"	"0.00000000"	1379117_at	NA	16.6013	0.00000008	1378334_a_at	Itpr2	18.1964	0
1369732_a_at	"St3gal2"	" -38.5068"	"0.00000000"	1387969_at	Cxcl10	-16.4933	0.00000009	1396763_at	LOC301455	25.1332	0
1394264_at	"RGD1561144_predicted"	" -38.4072"	"0.00000000"	1373892_at	Fbxl17_predicted	15.6726	0.0000002	1390552_at	Magi1	18.3569	0
1392444_at	"RGD1561144_predicted"	" -38.2809"	"0.00000000"	1383069_at	LOC680726	15.4429	0.00000026	1387809_at	Map2k6	16.5718	0
1376775_at	"RGD1309969"	" 38.2342"	"0.00000000"	1389150_at	NA	15.4163	0.00000027	1380017_at	NA	17.3843	0
1383757_at	NA	" -37.7027"	"0.00000000"	1374586_at	LOC689079	15.1824	0.00000034	1379597_at	NA	30.294	0
1369090_at	"Prkg2"	" 36.7766"	"0.00000000"	1378457_at	NA	15.0492	0.0000004	1368053_at	Pard3	27.7246	0
1377643_at	"Hoxd10_predicted"	" -36.0480"	"0.00000000"	1387809_at	Map2k6	14.884	0.00000048	1375224_at	Phlda3	-17.092	0
1380133_at	"Osr2"	" 35.8002"	"0.00000000"	1388738_at	RGD1563485_predicted	14.647	0.00000063	1374042_at	RGD1310453_pre	18.5935	0
1379107_at	"Slc4a4"	" 35.4450"	"0.00000000"	1394290_at	NA	14.2394	0.000001	1381607_at	Zfp407	19.1243	0
1373333_at	"MGC109340"	" 34.8190"	"0.00000000"	1390240_at	NA	14.2115	0.00000103	1388639_at	Bcas3	22.9047	0
1377635_at	"Fmo2"	" -34.7043"	"0.00000000"	1388825_at	NA	13.9677	0.00000138	1388738_at	Atrnl1	23.6839	0
1382031_at	NA	" -34.6443"	"0.00000000"	1384227_at	Ptprk	13.9273	0.00000144	1369777_a_at	Shank2	20.1273	0
1367969_at	"Prdx6"	" 33.6355"	"0.00000000"	1387306_a_at	Egr2	-13.7039	0.00000188	1373718_at	Tubb2b	-16.57	0
1386907_at	"Eno3"	" -32.4485"	"0.00000000"	1373207_at	NA	13.626	0.00000207	1395132_at	Utrn	16.9349	0
1388619_at	"Rdx"	" -32.3928"	"0.00000000"	1388639_at	RGD1560788_predicted	13.6151	0.0000021	1371520_at	Znf291	19.8905	0
1371615_at	"Dgat2"	" 30.7888"	"0.00000000"	1390552_at	LOC500261	13.4795	0.00000247	1380546_at	LOC298250	15.3376	0.00000001
1368168_at	"Slc34a2"	" -30.2798"	"0.00000000"	1397622_at	Ddx46	13.285	0.00000314	1390189_at	LOC298977	15.0793	0.00000001
1392647_at	"LOC687992"	" 30.1763"	"0.00000000"	1370905_at	Dock9	13.2743	0.00000318	1376132_at	Gtdc1	14.9889	0.00000001
1383146_at	"RGD1562629_predicted"	" 29.9941"	"0.00000000"	1376774_at	Exoc4	13.2104	0.00000344	1376747_at	Magi1	14.8143	0.00000001
1391045_at	"Dgat2"	" 29.9189"	"0.00000000"	1369995_at	Faf1	13.1854	0.00000355	1396872_at	Magi1	16.2841	0.00000001
1387092_at	"Fxyd4"	" -29.7550"	"0.00000000"	1374846_at	Clp1	-13.1748	0.00000359	1381005_at	LOC682925	15.6139	0.00000001
1372479_at	NA	" 28.1042"	"0.00000000"	1367764_at	Ccng1	-13.086	0.00000401	1374586_at	ARL15	14.8888	0.00000001
1387999_at	"Slc18a1"	" 27.8937"	"0.00000000"	1383166_at	Ncoa1_predicted	12.9898	0.00000453	1375888_at	LOC691036	15.7124	0.00000001
1378287_at	"Rdx"	" -27.5963"	"0.00000000"	1379107_at	Slc4a4	12.9203	0.00000494	1377749_at	NA	-16.201	0.00000001
1370371_a_at	"Ceacam10"	" -27.4230"	"0.00000000"	1394577_at	NA	12.908	0.00000502	1389186_at	NA	15.5324	0.00000001
1375925_at	NA	" -27.3230"	"0.00000000"	1379824_at	Tox_predicted	12.6908	0.00000661	1379117_at	NA	16.0637	0.00000001
1393808_at	"Wdr59"	" 27.1602"	"0.00000000"	1389102_at	RGD1566072_predicted	12.6331	0.00000712	1395613_at	Pard3	15.8741	0.00000001
1371360_at	"Ndrg1"	" 26.9024"	"0.00000000"	1391460_at	NA	12.6026	0.00000741	1370182_at	Ptprn2	15.1325	0.00000001
1388743_at	NA	" 26.5868"	"0.00000000"	1388723_at	Bre	12.3929	0.00000972	1372019_at	Med10	-15.064	0.00000001
1386344_at	"Ank"	" 26.4345"	"0.00000000"	1384504_at	NA	12.3816	0.00000986	1385271_at	RGD1565731_pre	15.0179	0.00000001
1368167_at	"Ctse"	" -26.2150"	"0.00000000"	1383795_at	NA	12.3352	0.00001048	1370474_at	Thrb	15.1202	0.00000001
1368858_at	"Ugt8"	" 25.8033"	"0.00000000"	1368321_at	Egr1	-12.3161	0.00001074	1387127_at	Atrn	14.3762	0.00000002
1371044_at	"Pde7a"	" -25.5031"	"0.00000000"	1379980_at	NA	12.1928	0.00001263	1367994_at	Dpyd	14.3682	0.00000002
1390812_a_at	"RGD1562829_predicted"	" 25.4623"	"0.00000000"	1397698_at	Fbxl17_predicted	12.1915	0.00001266	1387306_a_at	Egr2	-14.254	0.00000002
1368034_at	"Chgb"	" 25.2953"	"0.00000000"	1376132_at	LOC362129	12.1684	0.00001305	1376774_at	Exoc4	14.7666	0.00000002
1371835_at	"Prkacb"	" -25.2173"	"0.00000000"	1380387_at	RGD1559697_predicted	12.1569	0.00001325	1390183_at	LOC360990	14.2004	0.00000002
1376319_at	NA	" -25.1106"	"0.00000000"	1389388_at	NA	12.1538	0.0000133	1381402_at	NA	14.6533	0.00000002
1377803_at	"RGD1305351"	" -25.0838"	"0.00000000"	1389180_at	NA	12.1051	0.00001419	1370724_a_at	Nfia	14.4353	0.00000002
1374060_at	NA	" -24.7049"	"0.00000000"	1376071_at	NA	12.0399	0.00001548	1367847_at	Nupr1	-14.435	0.00000002

1369195_at	"Fabp2"	" 24.6860"	"0.00000000"	1384427_at	Mdm2_predicted	-12.0275	0.00001574	1374341_at	Thap7	-14.495	0.00000002
1394574_at	"Prdx6"	" 24.4506"	"0.00000000"	1383064_at	NA	12.0169	0.00001596	1383069_at	LOC680726	13.7689	0.00000003
1388337_at	"Np"	" -24.3058"	"0.00000000"	1390437_at	NA	12.0123	0.00001606	1372833_at	NA	13.9949	0.00000003
1393961_at	NA	" -24.0366"	"0.00000000"	1390193_at	NA	11.9069	0.0000185	1395203_at	NA	14.0382	0.00000003
1385709_x_at	"Ank"	" 23.8768"	"0.00000000"	1377760_at	Noc4l	-11.8075	0.00002116	1384227_at	Ptprk	14.0653	0.00000003
1368754_at	"P2ry6"	" -23.8485"	"0.00000000"	1376747_at	LOC500261	11.7744	0.00002213	1376416_at	RGD1309461	14.0782	0.00000003
1372476_at	"Fads3"	" -23.8424"	"0.00000000"	1381418_at	NA	11.7111	0.00002412	1374852_at	RGD1559909_predicted	-13.825	0.00000003
1393018_at	NA	" 23.7465"	"0.00000000"	1374593_at	Prkce	11.6867	0.00002493	1382117_at	RGD1560538_predicted	-13.815	0.00000003
1383472_at	"Aldh1b1"	" 23.6881"	"0.00000000"	1370163_at	Odc1	-11.6416	0.00002652	1368834_at	Camk2d	13.4361	0.00000004
1384594_at	"Ltk_predicted"	" -23.3358"	"0.00000000"	1385128_at	NA	11.5419	0.00003041	1387979_at	Golgb1	13.7429	0.00000004
1380139_at	NA	" 23.2565"	"0.00000000"	1379597_at	NA	11.4684	0.00003367	1374131_at	NA	13.4182	0.00000004
1392055_at	"RGD1561188_predicted"	" 23.2347"	"0.00000000"	1384198_at	Cova1_predicted	11.4609	0.00003402	1380180_at	NA	13.5369	0.00000004
1368435_at	"Cyp8b1"	" 23.0799"	"0.00000000"	1377751_at	NA	11.4164	0.00003619	1395029_at	NA	13.6515	0.00000004
1397053_at	"RGD1562829_predicted"	" 23.0328"	"0.00000000"	1386962_at	Plcb4	11.3998	0.00003704	1371056_at	Neo1	13.6742	0.00000004
1368282_at	"Dpep1"	" 23.0263"	"0.00000000"	1389011_at	RGD1305156	11.391	0.00003749	1369678_a_at	Nfia	13.4176	0.00000004
1369111_at	"Fabp1"	" 23.0065"	"0.00000000"	1389128_at	Wdfy3_predicted	11.3466	0.00003989	1389261_at	RGD1305508_predicted	-13.701	0.00000004
1370811_at	"Mpst"	" 22.8214"	"0.00000000"	1368834_at	Camk2d	11.3461	0.00003992	1380387_at	RGD1559697_predicted	13.4906	0.00000004
1387669_a_at	"Ephx1"	" 22.7338"	"0.00000000"	1392972_at	Trio	11.2973	0.00004274	1371785_at	Tnfrsf12a	-13.603	0.00000004
1370420_at	"Srd5a1"	" 22.4589"	"0.00000000"	1377884_at	RGD1560155_predicted	11.2534	0.00004546	1396076_at	NA	13.2952	0.00000005
1393439_a_at	"Ank"	" 22.4082"	"0.00000000"	1373430_at	Baz2b_predicted	11.2149	0.00004799	1390453_at	NA	13.3053	0.00000005
1380536_at	"RGD1305928_predicted"	" 22.3846"	"0.00000000"	1388870_at	RGD1560397_predicted	11.084	0.00005777	1377697_at	RGD1562438_predicted	13.2518	0.00000005
1387315_at	"Sftpd"	" -22.3517"	"0.00000000"	1390453_at	NA	11.0801	0.00005808	1378902_at	Rsafd1_predicted	13.2729	0.00000005
1381528_at	"St3gal2"	" -22.3068"	"0.00000000"	1389923_at	Btbd9	11.0062	0.00006454	1387002_at	G10	-13.093	0.00000006
1390569_at	"Cndp1"	" 22.3039"	"0.00000000"	1383743_at	Lrrc16_predicted	10.9767	0.00006733	1387405_at	Garnl1	13.1193	0.00000006
1383825_at	"Rdx"	" -22.2667"	"0.00000000"	1373502_at	Dym_predicted	10.9139	0.00007368	1380912_at	Glrp1_predicted	13.0252	0.00000006
1385175_at	"Hoxb13_predicted"	" -22.2419"	"0.00000000"	1376194_at	NA	10.8766	0.00007775	1396831_at	Glrp1_predicted	13.161	0.00000006
1383343_at	"LOC690559"	" 22.2224"	"0.00000000"	1396872_at	LOC500261	10.8254	0.00008373	1372449_at	NA	13.1055	0.00000006
1384772_at	"Prkacb"	" -22.1993"	"0.00000000"	1374591_at	RGD1561090_predicted	10.7905	0.00008807	1373207_at	NA	13.1444	0.00000006
1387988_at	"Hsd3b6"	" 22.0762"	"0.00000000"	1387116_at	Dnajb9	-10.7842	0.00008888	1377760_at	Noc4l	-13.128	0.00000006
1390825_at	"Slc35b3_predicted"	" 21.9488"	"0.00000000"	1385271_at	RGD1565731_predicted	10.7753	0.00009004	1375686_at	Ppil3	-13.151	0.00000006
1374073_at	"RGD1309472"	" -21.9429"	"0.00000000"	1389310_at	NA	10.7091	0.00009915	1371724_at	Rexo2	-13.068	0.00000006
1370746_at	"Prkacb"	" -21.8730"	"0.00000000"	1379663_at	Cachd1_predicted	10.6794	0.00010355	1395148_at	RGD1561090_predicted	13.0666	0.00000006
1395810_at	NA	" 21.7302"	"0.00000000"	1379578_at	Zbtb20_predicted	10.6709	0.00010485	1390987_at	RGD1565283_predicted	13.1237	0.00000006
1367566_at	"Scgb1a1"	" -21.6560"	"0.00000000"	1383103_at	LOC680620	10.6508	0.00010798	1378943_at	RGD1566072_predicted	13.0313	0.00000006
1379445_at	NA	" 21.6463"	"0.00000000"	1383931_at	Ptprk	10.6384	0.00010997	1368123_at	Igf1r	12.8671	0.00000007
1374029_at	NA	" 21.4056"	"0.00000000"	1383485_at	Mdm2_predicted	-10.6211	0.00011278	1395595_at	Mki67ip	-12.797	0.00000007
1374284_at	"Rassf4"	" 21.3211"	"0.00000000"	1368053_at	Pard3	10.6143	0.00011392	1383494_at	RGD1562788_predicted	12.8094	0.00000007
1395153_at	"Cldn15_predicted"	" 21.2839"	"0.00000000"	1371949_at	Bzw1	-10.554	0.0001245	1389923_at	Btbd9	12.7211	0.00000008
1382612_at	"Hoxa9_predicted"	" 21.1488"	"0.00000000"	1389431_at	Prkdc_predicted	10.5327	0.00012849	1388443_at	Cdk2ap1_predicted	-12.771	0.00000008
1385464_at	"Foxq1"	" -21.1346"	"0.00000000"	1389159_at	NA	10.5147	0.00013194	1374846_at	Clp1	-12.584	0.00000008
1369785_at	"Ppat"	" -21.1199"	"0.00000000"	1368955_at	Cask	10.5121	0.00013246	1397698_at	Fbxl17_predicted	12.5791	0.00000008
1377123_at	"Slc27a4"	" 21.0207"	"0.00000000"	1368019_at	Frap1	10.486	0.00013767	1382794_at	Galnt10	12.7072	0.00000008
1379936_at	"Tpm1"	" -20.9438"	"0.00000000"	1383491_at	Isg20l1_predicted	-10.4184	0.00015223	1391046_at	LOC498295	12.6438	0.00000008
1371101_at	"Ryk"	" -20.9376"	"0.00000000"	1391916_at	NA	10.3556	0.00016719	1383288_at	Mdm2_predicted	-12.668	0.00000008
1372374_at	"Car1_predicted"	" 20.7692"	"0.00000000"	1390289_at	RGD1311375_predicted	10.2768	0.00018817	1383493_at	NA	12.6422	0.00000008
1380285_at	"Chrd"	" 20.7585"	"0.00000000"	1371633_at	Ctnnbl1	10.225	0.00020347	1379277_at	NA	12.6753	0.00000008
1370547_at	"Pzp"	" 20.6081"	"0.00000000"	1381607_at	RGD1310645_predicted	10.2098	0.00020819	1382758_at	P7	12.6648	0.00000008
1374531_at	NA	" 20.5694"	"0.00000000"	1389355_at	Ier5	-10.2095	0.00020827	1371631_at	RGD1304686	-12.576	0.00000008
1391251_at	NA	" 20.5572"	"0.00000000"	1383498_at	NA	10.0968	0.00024714	1372845_at	Rpp21	-12.729	0.00000008
1385246_at	NA	" 20.5350"	"0.00000000"	1390010_at	Ncoa1_predicted	10.0272	0.00027487	1387374_at	Tcf12	12.6585	0.00000008

1386926_at	"Acsl5"	" -20.2690"	"0.00000000"
1387791_at	"Ace"	" 20.2164"	"0.00000000"
1368281_at	"Dpep1"	" 20.2036"	"0.00000000"
1375863_a_at	NA	" -20.2012"	"0.00000000"
1370530_a_at	"Pld1"	" 19.9225"	"0.00000000"
1378606_at	NA	" 19.8974"	"0.00000000"
1377058_at	"RGD1564160_predicted"	" -19.8571"	"0.00000000"
1398341_at	"RGD1559720_predicted"	" 19.7327"	"0.00000000"
1385767_at	"LOC304000"	" 19.4867"	"0.00000000"
1369381_a_at	"Slc15a1"	" -19.4668"	"0.00000000"
1383978_at	NA	" -19.4660"	"0.00000000"
1373028_at	"Ryk"	" -19.3243"	"0.00000000"
1377412_at	NA	" 19.3113"	"0.00000000"
1370299_at	"Aldob"	" 19.2783"	"0.00000000"
1387123_at	"Cyp17a1"	" -19.2631"	"0.00000000"
1369531_at	"Sult1c2"	" 19.2371"	"0.00000000"
1375295_at	"Cs"	" -19.2104"	"0.00000000"
1387235_at	"Chga"	" 19.1331"	"0.00000000"
1393945_at	NA	" 19.0817"	"0.00000000"
1395814_at	NA	" -19.0627"	"0.00000000"
1374081_at	"Casc4_predicted"	" 19.0408"	"0.00000000"
1387121_a_at	"Ndrg2"	" -19.0340"	"0.00000000"
1391684_at	"Tmem14a_predicted"	" 18.9416"	"0.00000000"
1372330_at	"LOC652955"	" 18.8537"	"0.00000000"
1383104_at	"RGD1561459_predicted"	" 18.7808"	"0.00000000"
1369007_at	"Nr4a2"	" -18.7535"	"0.00000000"
1369162_at	"Gucy2c"	" 18.7338"	"0.00000000"
1375266_at	"Slc6a9"	" -18.6721"	"0.00000000"
1370623_at	"Fgl2"	" 18.6662"	"0.00000000"
1390471_at	"Tpm1"	" -18.6382"	"0.00000000"
1395355_at	NA	" 18.5301"	"0.00000000"
1370392_at	"Trpm4"	" 18.5263"	"0.00000000"
1388138_at	"Thbs4"	" -18.4667"	"0.00000001"
1389142_at	"LOC691966"	" 18.3565"	"0.00000001"
1395148_at	"RGD1561090_predicted"	" 18.3310"	"0.00000001"
1372255_at	"Rars_predicted"	" -18.3043"	"0.00000001"
1370468_at	"Slc13a1"	" 18.2699"	"0.00000001"
1373586_at	NA	" -18.1749"	"0.00000001"
1390050_at	"LOC680692"	" -18.1462"	"0.00000001"
1375247_at	"Mgll"	" 18.0481"	"0.00000001"
1370948_a_at	"Marcks"	" -18.0391"	"0.00000001"
1371479_at	"Mettl7a"	" 17.9921"	"0.00000001"
1377116_at	"Rnasel"	" 17.8808"	"0.00000001"
1393057_at	"LOC308320"	" -17.8064"	"0.00000001"
1388644_at	"Mgll"	" 17.7546"	"0.00000001"
1368869_at	"Akap12"	" -17.7239"	"0.00000001"
1387193_a_at	"Spink1"	" -17.6947"	"0.00000001"
1392905_at	"Gng2"	" -17.6916"	"0.00000001"
1374815_at	"Stard3nl"	" 17.5289"	"0.00000001"
1370448_at	"Gpc2"	" 17.5112"	"0.00000001"

1383494_at	RGD1562788_predicted	10.0146	0.00028024
1389655_at	RGD1306819	-10.0119	0.0002814
1391169_at	RGD1562562_predicted	9.9938	0.00028934
1381445_at	Esrrg	9.9694	0.0003004
1373265_at	NA	9.891	0.00033901
1388554_at	Bzw1	-9.8836	0.00034289
1389613_at	NA	9.8826	0.00034341
1373530_at	Ccne1	-9.7942	0.00039399
1379277_at	NA	9.717	0.00044445
1381871_at	LOC500261	9.6585	0.00048718
1373393_at	LOC299907	9.6582	0.00048739
1389476_at	RGD1307100	9.6577	0.00048781
1379500_at	LOC498404	-9.6468	0.00049623
1374466_at	NA	9.6383	0.00050291
1398392_at	NA	9.6314	0.00050841
1383288_at	Mdm2_predicted	-9.6285	0.00051072
1370893_at	Acaca	9.5978	0.00053607
1390217_at	Garnl1	9.59	0.00054276
1382993_at	Bbc3	-9.5227	0.00060386
1379244_at	NA	9.5205	0.00060595
1372744_at	Pkp4_predicted	9.4874	0.00063866
1386959_a_at	Map2k5	9.4688	0.00065794
1376273_at	Rnasen	9.4105	0.00072207
1389371_at	NA	9.3632	0.00077909
1369777_a_at	Shank2	9.3288	0.00082334
1367790_at	Snd1	9.3223	0.00083206
1383194_a_at	NA	9.2969	0.00086683
1393772_at	NA	-9.2703	0.00090489
1374743_at	RGD1565362_predicted	9.2656	0.00091174
1394510_at	NA	9.2581	0.00092295
1392044_at	NA	9.2542	0.00092887
1389291_at	Chchd3_predicted	9.2397	0.00095083
1377576_at	Ppa2_predicted	9.2361	0.00095646
1395998_at	Nol5a	-9.1575	0.00108689
1373479_at	Ppp3ca	9.1461	0.00110733
1399056_at	LOC365592	9.129	0.00113858
1388352_at	Nat5_predicted	-9.1106	0.00117332
1392653_at	NA	9.1071	0.00118007
1385798_at	NA	9.0652	0.00126406
1367994_at	Dpyd	9.0615	0.00127172
1390343_at	Ccnc	-9.0489	0.00129838
1388622_at	Nol5a	-9.0244	0.00135172
1390709_at	Trio	9.0107	0.00138276
1395148_at	RGD1561090_predicted	9.0001	0.00140696
1374131_at	NA	8.9802	0.00145392
1381685_a_at	RGD1305773_predicted	8.974	0.00146897
1390865_at	LOC681395	8.9622	0.00149792
1371571_at	App	8.8653	0.0017594
1367736_at	Rraga	-8.8599	0.00177532
1392770_at	Neo1	8.8319	0.00186033

1371505_at	Hnrpc	-12.474	0.00000009
1374495_at	Lrba_predicted	12.5191	0.00000009
1390010_at	Ncoa1_predicted	12.46	0.00000009
1389431_at	Prkdc_predicted	12.5524	0.00000009
1383017_at	Ptprm	12.466	0.00000009
1378445_at	RGD1561537_predicted	12.4925	0.00000009
1388906_at	RGD1564930_predicted	12.5443	0.00000009
1398876_at	Abcf1	-12.278	0.0000001
1388601_at	Abt1	-12.272	0.0000001
1397622_at	Ddx46	12.2408	0.0000001
1372189_at	Dnajc13_predicted	12.2992	0.0000001
1388154_at	E2f5	-12.405	0.0000001
1368225_at	Exoc2	12.2781	0.0000001
1373892_at	Fbxl17_predicted	12.3882	0.0000001
1377653_at	LOC366669	12.3779	0.0000001
1389024_at	LOC498353	12.2354	0.0000001
1382001_at	LOC502710	12.3495	0.0000001
1372815_at	Magoh_predicted	-12.237	0.0000001
1389167_at	Mapkap1	12.3025	0.0000001
1390437_at	NA	12.2374	0.0000001
1376103_at	NA	12.242	0.0000001
1389371_at	NA	12.3234	0.0000001
1374262_at	NA	12.3431	0.0000001
1378091_at	NA	12.3439	0.0000001
1395704_at	NA	12.3707	0.0000001
1389698_at	NA	12.3864	0.0000001
1370489_a_at	Plcb4	12.2778	0.0000001
1388310_at	Sui1-rs1_predicted	-12.225	0.0000001
1388130_at	Epha1_predicted	-12.128	0.00000011
1388990_at	Mki67ip	-12.127	0.00000011
1376275_at	NA	-12.112	0.00000011
1394510_at	NA	12.0927	0.00000011
1372708_at	NA	12.0942	0.00000011
1382162_at	NA	12.166	0.00000011
1386529_at	NA	12.1854	0.00000011
1390204_at	NA	12.1995	0.00000011
1388180_at	Phax	-12.202	0.00000011
1389476_at	RGD1307100	12.1753	0.00000011
1391731_at	NA	12.0494	0.00000012
1373419_at	Ptprg	12.0774	0.00000012
1388780_at	Terf2ip	-12.064	0.00000012
1367719_at	Dd5	11.9843	0.00000013
1383382_at	LOC681740	11.9639	0.00000013
1393409_at	NA	11.9913	0.00000013
1368106_at	Plk2	-12.003	0.00000013
1372634_at	Adprhl2_predicted	-11.912	0.00000014
1371423_at	Mrpl41	-11.872	0.00000014
1394456_at	NA	11.9004	0.00000014
1373265_at	NA	11.9186	0.00000014
1392316_at	Ptprg	11.9026	0.00000014

1368550_at	"Foxq1"	" -17.4803"	"0.00000001"	1385109_at	Josd3	-8.8283	0.00187136	1373965_at	RGD1310931_predicted	-11.884	0.00000014
1375874_at	"RGD1309759_predicted"	" -17.4612"	"0.00000001"	1369630_at	Adk	8.8283	0.00187143	1391608_at	RGD1565449_predicted	11.8761	0.00000014
1391458_at	"Ndrg1"	" 17.4257"	"0.00000001"	1390502_at	RGD1309104_predicted	8.7794	0.00203102	1390267_at	Fars2	11.8129	0.00000015
1367888_at	"Pcdh21"	" 17.3074"	"0.00000002"	1390987_at	RGD1565283_predicted	8.7788	0.00203325	1390217_at	Garnl1	11.8235	0.00000015
1372641_at	"Alpk3_predicted"	" -17.2981"	"0.00000002"	1368117_at	Gphn	8.7721	0.00205611	1379242_at	LOC684996	-11.83	0.00000015
1395750_at	NA	" -17.2707"	"0.00000002"	1389556_at	Kifap3_predicted	8.7374	0.00217958	1373477_at	Mrpl19	-11.765	0.00000015
1393777_at	NA	" 17.2669"	"0.00000002"	1389009_at	Rsrc1	8.7307	0.00220436	1383702_at	NA	-11.771	0.00000015
1377660_at	"RGD1309350_predicted"	" -17.2277"	"0.00000002"	1392818_at	Gas5	-8.7226	0.00223435	1377603_at	Snx24	11.7631	0.00000015
1379909_at	"Gkap1"	" 17.0947"	"0.00000002"	1368980_at	Plce1	8.7093	0.00228494	1370905_at	Dock9	11.7041	0.00000016
1398270_at	"Bmp2"	" -17.0696"	"0.00000002"	1376690_at	Surb7_predicted	-8.7027	0.00231079	1375562_at	NA	11.6945	0.00000016
1383584_at	"Vmd2l1_predicted"	" -17.0525"	"0.00000002"	1367671_at	Pcna	-8.6952	0.00234023	1394577_at	NA	11.7174	0.00000016
1379108_at	"RGD1559968_predicted"	" 17.0455"	"0.00000002"	1383278_at	Centg2_predicted	8.6893	0.0023636	1376060_at	NA	11.7318	0.00000016
1372714_at	"RGD1307778"	" -17.0385"	"0.00000002"	1373145_at	RGD1560511_predicted	8.6795	0.00240293	1372882_at	RGD1308635_predicted	-11.722	0.00000016
1373169_at	"Agpat5_predicted"	" -17.0333"	"0.00000002"	1388611_at	Tcea3	8.678	0.00240913	1398965_at	LOC501069	11.6341	0.00000017
1381968_at	"Creg_predicted"	" -17.0286"	"0.00000002"	1376337_at	Smarca2	8.6726	0.00243115	1374593_at	Prkce	11.6439	0.00000017
1370531_a_at	"Pld1"	" 16.9763"	"0.00000002"	1396834_at	Braf	-8.6642	0.0024658	1389226_at	Stag1_predicted	11.6592	0.00000017
1370943_at	"Sult1c2"	" 16.9656"	"0.00000002"	1377603_at	Snx24	8.6476	0.00253595	1379578_at	Zbtb20_predicted	11.6757	0.00000017
1373483_at	"Ankrd47_predicted"	" -16.9047"	"0.00000002"	1372255_at	Rars_predicted	-8.6218	0.00264923	1373411_at	Eif1b_predicted	-11.579	0.00000018
1372308_at	NA	" 16.8900"	"0.00000002"	1373285_at	NA	8.5861	0.00281489	1398922_at	NA	11.5961	0.00000018
1379546_at	NA	" 16.8862"	"0.00000002"	1369680_at	Slc2a13	8.5843	0.0028235	1374764_at	RGD1305605_predicted	-11.578	0.00000018
1371470_at	"Adh4"	" 16.8824"	"0.00000002"	1377926_at	Centg2_predicted	8.5834	0.00282822	1369995_at	Faf1	11.5546	0.00000019
1367501_at	"LOC498351"	" -16.8190"	"0.00000003"	1396076_at	NA	8.5814	0.00283759	1398371_at	RGD1304751	11.5533	0.00000019
1375523_at	"Marcks"	" -16.8107"	"0.00000003"	1390648_at	Herc2_predicted	8.567	0.00290828	1388408_at	RGD1307129	-11.527	0.00000019
1377761_at	"Gfpt2"	" -16.7853"	"0.00000003"	1392498_at	LOC691318	8.5586	0.00294985	1374034_at	Cars_predicted	-11.48	0.0000002
1370529_a_at	"Pld1"	" 16.7302"	"0.00000003"	1377254_a_at	Cohh1_predicted	8.5142	0.00318236	1370684_s_at	LOC501546	11.5117	0.0000002
1374300_at	"Entpd4_predicted"	" -16.6658"	"0.00000003"	1382441_at	Arid1b	8.5138	0.00318448	1383103_at	LOC680620	11.4663	0.0000002
1371922_at	NA	" 16.6608"	"0.00000003"	1375358_at	NA	8.4995	0.00326343	1384163_at	NA	-11.511	0.0000002
1370313_at	"Acot7"	" 16.6462"	"0.00000003"	1390692_at	Ctps_predicted	-8.4818	0.00336352	1371427_at	RGD1306911_predicted	-11.47	0.0000002
1392993_at	NA	" 16.5837"	"0.00000003"	1368279_at	Mllt3	8.4715	0.00342372	1371478_at	RGD1307752	-11.458	0.0000002
1383447_at	"Etv5_predicted"	" 16.5684"	"0.00000003"	1371718_at	Sra1	-8.4505	0.00354947	1377674_at	RGD1560612_predicted	11.5155	0.0000002
1367805_at	"Gls"	" 16.5672"	"0.00000003"	1375995_at	RGD1304592_predicted	8.4398	0.00361492	1396144_at	RGD1561817_predicted	11.4912	0.0000002
1392274_at	"Pdgfc"	" 16.5530"	"0.00000004"	1370503_s_at	Epb4.1l3	8.4345	0.0036485	1374599_at	Herc1_predicted	11.4417	0.00000021
1369186_at	"Casp1"	" 16.5244"	"0.00000004"	1389587_at	Umps	-8.4296	0.0036792	1374857_at	LOC499709	-11.405	0.00000021
1386922_at	"Ca2"	" 16.4821"	"0.00000004"	1372059_at	RGD1309437	8.4276	0.00369175	1394448_at	NA	11.4437	0.00000021
1387867_at	"Aldh9a1"	" 16.4027"	"0.00000004"	1371520_at	Znf291	8.4065	0.00382863	1376686_at	Tmtc2_predicted	11.4329	0.00000021
1368528_at	"Mic2l1"	" 16.3957"	"0.00000004"	1394940_at	RGD1311381_predicted	-8.4035	0.00384831	1370893_at	Acaca	11.341	0.00000022
1377672_at	"Sult1c2"	" 16.2987"	"0.00000005"	1388953_at	Gnl3	-8.3918	0.00392707	1388643_at	Fut8	11.3635	0.00000022
1380425_at	"Rnasel"	" 16.2715"	"0.00000005"	1368371_at	Kcnq1	8.3339	0.0043405	1399000_at	LOC291411	11.3492	0.00000022
1374586_at	"LOC689079"	" 16.2525"	"0.00000005"	1376619_at	RGD1561090_predicted	8.2859	0.00471686	1382500_at	NA	-11.399	0.00000022
1373968_at	"RGD1561940_predicted"	" 16.2377"	"0.00000005"	1390423_at	Phr1_predicted	8.2703	0.00484675	1372642_at	NA	-11.375	0.00000022
1384201_at	"Cs"	" -16.2113"	"0.00000005"	1367719_at	Dd5	8.2698	0.00485144	1389613_at	NA	11.3693	0.00000022
1372449_at	NA	" 16.2025"	"0.00000005"	1372833_at	NA	8.26	0.00493461	1370318_at	Pik4ca	11.353	0.00000022
1387263_at	"Pklr"	" 16.1808"	"0.00000005"	1390267_at	Fars2	8.2594	0.00494024	1372066_at	RGD1310022	-11.355	0.00000022
1388102_at	"Ltb4dh"	" 16.1749"	"0.00000005"	1389167_at	Mapkap1	8.2566	0.00496408	1390492_a_at	Usp40	11.3601	0.00000022
1368608_at	"Cyp2f4"	" -16.1523"	"0.00000005"	1373030_at	NA	-8.2524	0.00500018	1371484_at	LOC690349	-11.321	0.00000023
1372911_at	NA	" 16.1502"	"0.00000005"	1371498_at	MGC125271	-8.2468	0.00504961	1383102_at	NA	11.3094	0.00000023
1374687_at	NA	" 16.0934"	"0.00000006"	1370153_at	Gdf15	-8.2435	0.00507893	1387911_at	Rabggtb	-11.33	0.00000023
1398431_at	"Car8"	" 16.0500"	"0.00000006"	1391445_at	NA	-8.214	0.00534736	1376062_at	Sdc1	-11.283	0.00000023
1387878_at	"Glud1"	" 16.0460"	"0.00000006"	1388646_at	RGD1311784_predicted	8.2028	0.0054526	1390709_at	Trio	11.2841	0.00000023
1372443_at	"Lrp11_predicted"	" 16.0227"	"0.00000006"	1367456_at	Ube2d3	-8.1996	0.00548312	1372218_at	Wdr12	-11.308	0.00000023

1394259_at	"Cldn15_predicted"	" 15.9837"	"0.00000007"	1391411_at	RGD1560248_predicted	8.1936	0.00554166	1377254_a_at	Cohh1_predicted	11.2656	0.00000024
1390884_a_at	"B3gnt7"	" -15.9798"	"0.00000007"	1389562_at	NA	8.1851	0.0056245	1381905_at	Herpud1	11.246	0.00000024
1369770_at	"Sstr1"	" -15.9428"	"0.00000007"	1368123_at	Igf1r	8.1819	0.00565642	1384427_at	Mdm2_predicted	-11.235	0.00000024
1388511_at	"Centd2"	" 15.9301"	"0.00000007"	1378492_at	Farp1_predicted	8.1808	0.00566705	1382144_at	Mrpl47	-11.243	0.00000024
1367733_at	"Ca2"	" 15.9066"	"0.00000007"	1389301_at	LOC686892	8.169	0.0057853	1391460_at	NA	11.2546	0.00000024
1370072_at	"Mme"	" -15.8711"	"0.00000007"	1394849_at	Zbtb20_predicted	8.1689	0.00578692	1381612_at	NA	11.2624	0.00000024
1392900_at	"LOC691149"	" -15.8637"	"0.00000007"	1372863_at	Phr1_predicted	8.1625	0.00585161	1372941_at	Pdrg1	-11.226	0.00000024
1367712_at	"Timp1"	" -15.8560"	"0.00000007"	1395704_at	NA	8.1624	0.0058531	1383805_at	RGD1561817_predicted	11.2605	0.00000024
1394313_at	"Tnfrsf11a_predicted"	" 15.8408"	"0.00000008"	1388167_at	Nfib	8.1621	0.00585583	1399004_at	LOC679572	-11.207	0.00000025
1367900_at	"Gyg1"	" 15.8374"	"0.00000008"	1376465_at	RGD1563869_predicted	8.1615	0.00586209	1391445_at	NA	-11.201	0.00000025
1374043_at	"Gramd3"	" 15.8247"	"0.00000008"	1389226_at	Stag1_predicted	8.1603	0.00587486	1384463_at	NA	11.2005	0.00000025
1390386_at	"Casp3"	" 15.8006"	"0.00000008"	1374225_at	LOC500700	8.1524	0.00595623	1367518_at	NA	11.2182	0.00000025
1396178_at	"C1qtnf4_predicted"	" 15.7941"	"0.00000008"	1371505_at	Hnrpc	-8.1498	0.00598432	1389033_at	RGD1306917_predicted	-11.201	0.00000025
1373847_at	"Tm4sf1_predicted"	" -15.7792"	"0.00000008"	1371963_at	Pcca	8.1492	0.0059896	1374806_at	Sfn_predicted	-11.152	0.00000026
1391841_at	"Tpm1"	" -15.7640"	"0.00000008"	1389287_at	NA	8.1424	0.00606242	1371512_at	Bre	-11.096	0.00000027
1384177_at	"Gramd3"	" 15.7606"	"0.00000008"	1384280_at	NA	8.1399	0.00608875	1398955_at	Cops8	-11.13	0.00000027
1397278_at	NA	" -15.7597"	"0.00000008"	1374349_at	Ctdspl_predicted	8.1381	0.00610853	1392818_at	Gas5	-11.122	0.00000027
1387410_at	"Nr4a2"	" -15.7514"	"0.00000008"	1368070_at	Stx8	8.1365	0.00612497	1385109_at	Josd3	-11.077	0.00000027
1388103_at	"Tmem37"	" -15.6919"	"0.00000009"	1371402_at	Atp6v1b2	-8.1143	0.00636979	1394535_at	Kif16b_predicted	11.1315	0.00000027
1387209_at	"Lztr2"	" 15.6892"	"0.00000009"	1391577_at	Pgam5	-8.0926	0.00661792	1379290_at	LOC300963	11.0807	0.00000027
1371499_at	"Cd9"	" -15.6641"	"0.00000009"	1388403_at	LOC361596	8.0923	0.00662087	1371485_at	NA	11.0995	0.00000027
1367673_at	"Selenbp1"	" 15.6446"	"0.00000009"	1388645_at	RGD1307982	8.0883	0.00666808	1390965_at	NA	11.1074	0.00000027
1395373_at	NA	" 15.6306"	"0.00000010"	1379396_at	Elmo1_predicted	8.0831	0.00672955	1379445_at	NA	11.1188	0.00000027
1369073_at	"Nr1h4"	" 15.5739"	"0.00000010"	1374886_at	Bcs1l	-8.0806	0.00675858	1388622_at	Nol5a	-11.125	0.00000027
1370949_at	"Marcks"	" -15.5682"	"0.00000010"	1389566_at	Ccnb2	8.0775	0.00679677	1383115_at	Prkg1	-11.131	0.00000027
1382387_at	"Tmem16a_predicted"	" -15.5623"	"0.00000010"	1386586_at	MGC125015	-8.0756	0.00681861	1374253_at	RGD1307235_predicted	11.0954	0.00000027
1374771_at	NA	" 15.5387"	"0.00000011"	1380527_at	Pde7b	8.0665	0.00692909	1394786_at	Sorl1_predicted	11.1354	0.00000027
1393840_at	"Gnat2_predicted"	" -15.5028"	"0.00000011"	1376868_at	Cobll1_predicted	8.058	0.00703496	1370807_at	Tmem49	-11.084	0.00000027
1375823_at	"Slc4a4"	" 15.4803"	"0.00000011"	1390189_at	LOC298977	8.0523	0.00710625	1388394_at	Aars	-11.07	0.00000028
1368180_s_at	"Gsta2"	" -15.4401"	"0.00000012"	1385043_at	RGD1565362_predicted	8.0382	0.0072847	1377926_at	Centg2_predicted	11.0389	0.00000028
1398445_at	NA	" 15.4378"	"0.00000012"	1371828_at	RGD1310861	-8.0362	0.00731146	1376683_at	NA	11.0602	0.00000028
1373248_at	"RGD1565800_predicted"	" -15.4185"	"0.00000012"	1391046_at	LOC498295	8.0352	0.00732455	1389862_at	RGD1305327	-11.054	0.00000028
1371996_at	"Aebp2_predicted"	" -15.4030"	"0.00000012"	1379832_at	Polr2d_predicted	-8.0343	0.00733634	1373017_at	Suclg2	11.0578	0.00000028
1373078_at	"RGD1564895_predicted"	" 15.3668"	"0.00000013"	1371818_at	NA	-8.0303	0.00738784	1368019_at	Frap1	11.0192	0.00000029
1378889_at	NA	" 15.3507"	"0.00000013"	1382001_at	LOC502710	8.0278	0.0074214	1374494_at	NA	11.0309	0.00000029
1368447_x_at	"Spink1"	" -15.3322"	"0.00000014"	1379352_at	Pip5k1a	8.0229	0.00748573	1374424_at	Prmt5_predicted	-10.999	0.00000029
1376973_at	"Sdcbp2"	" 15.2886"	"0.00000014"	1391607_at	RGD1566319_predicted	-8.013	0.00761799	1390579_at	RGD1305222_predicted	-11.021	0.00000029
1388300_at	"Mgst3_predicted"	" 15.2800"	"0.00000014"	1368650_at	Klf10	-7.9823	0.00804517	1389102_at	RGD1566072_predicted	11.0018	0.00000029
1373432_at	"Marcks"	" -15.2788"	"0.00000014"	1386916_at	Aco1	7.9741	0.00816274	1376065_at	Rrs1_predicted	-11.005	0.00000029
1391125_at	NA	" -15.2176"	"0.00000015"	1373870_at	RGD1305486	-7.9696	0.00822937	1390040_at	Bre	10.9738	0.0000003
1384668_at	NA	" -15.2159"	"0.00000015"	1388710_at	Rreb1_predicted	7.9651	0.0082957	1384076_at	Cachd1_predicted	10.9412	0.0000003
1370813_at	"Gstm5"	" -15.1884"	"0.00000016"	1377653_at	LOC366669	7.9645	0.00830363	1372225_at	Cap350	10.9494	0.0000003
1368905_at	"Ces2"	" 15.1786"	"0.00000016"	1367518_at	NA	7.9624	0.00833554	1369976_at	Dynll1	-10.945	0.0000003
1389580_at	"Smarca3_predicted"	" 15.1785"	"0.00000016"	1395203_at	NA	7.9512	0.00850239	1374777_at	LOC502894	-10.956	0.0000003
1399040_at	"Gba2"	" -15.1387"	"0.00000017"	1388546_at	Ablim1_predicted	7.9492	0.00853295	1382084_at	LOC682100	10.9803	0.0000003
1390187_at	NA	" -15.1004"	"0.00000018"	1371202_a_at	Nfib	7.9299	0.0088329	1375452_at	Mgea6_predicted	10.9902	0.0000003
1388440_at	"Aph1b"	" -15.0896"	"0.00000018"	1370330_at	Sipa1l1	7.9278	0.00886542	1377208_at	NA	10.9757	0.0000003
1389587_at	"Umps"	" -15.0739"	"0.00000018"	1393478_at	NA	7.918	0.00902244	1383166_at	Ncoa1_predicted	10.9636	0.0000003
1380186_at	NA	" -15.0621"	"0.00000018"	1386882_at	Tctex1	-7.9135	0.009094	1368173_at	Nol5	-10.967	0.0000003
1390031_at	"RGD1311873"	" -15.0288"	"0.00000019"	1368225_at	Exoc2	7.9068	0.00920359	1370177_at	PVR	-10.953	0.0000003

1383542_at	NA	" -15.0151"	"0.00000019"	1389608_at	Abcf2_predicted	-7.8959	0.00938512	1398919_at	RGD1304704	-10.95	0.0000003
1391005_at	"Fbxl4_predicted"	" 14.9689"	"0.00000021"	1390492_a_at	Usp40	7.8955	0.00939186	1392452_at	RGD1563764_predicted	10.9307	0.0000003
1371089_at	"Yc2"	" 14.9663"	"0.00000021"	1370294_a_at	Cdc20	7.8936	0.00942472	1374824_at	Rpl37	10.9548	0.0000003
1370467_at	"Slc13a1"	" 14.9661"	"0.00000021"	1369679_a_at	Nfia	7.8807	0.00964402	1376067_at	Cnot2	10.8937	0.00000031
1374610_at	NA	" 14.9654"	"0.00000021"	1372739_at	Tspan31	-7.8723	0.00979029	1395139_at	Kif16b_predicted	10.9129	0.00000031
1369407_at	"Tnfrsf11b"	" -14.9425"	"0.00000021"	1377263_at	Havcr2	-7.8662	0.00989843	1381871_at	LOC500261	10.9266	0.00000031
1391532_at	"Rap2ip"	" 14.9397"	"0.00000021"	1397748_at	NA	-7.8635	0.0099462	1390240_at	NA	10.8928	0.00000031
1369179_a_at	"Pparg"	" 14.9129"	"0.00000022"	1388816_at	Dlgh1	7.8267	0.01062539	1383795_at	NA	10.9073	0.00000031
1385107_at	"Slc6a14"	" -14.8582"	"0.00000023"	1377674_at	RGD1560612_predicted	7.8203	0.01074857	1388352_at	Nat5_predicted	-10.894	0.00000031
1370881_at	"Tst"	" 14.8543"	"0.00000023"	1370474_at	Thrb	7.8174	0.01080618	1398961_at	Pfn2	10.9091	0.00000031
1368219_at	"Clcn2"	" 14.8090"	"0.00000025"	1395613_at	Pard3	7.7933	0.01128419	1374651_at	Dopey2_predicted	10.8669	0.00000032
1386940_at	"Timp2"	" -14.7843"	"0.00000026"	1372218_at	Wdr12	-7.7872	0.01141035	1373038_at	Mllt10	10.8599	0.00000032
1387942_at	"Slc35e4"	" 14.7805"	"0.00000026"	1373191_at	Rab34	7.7691	0.01178764	1377703_at	Pip5k1a	-10.883	0.00000032
1384101_at	"LOC682507"	" -14.7717"	"0.00000026"	1374078_at	RGD1560612_predicted	7.7459	0.01229226	1374945_at	RGD1359191	-10.852	0.00000032
1373842_at	"LOC682507"	" -14.7658"	"0.00000026"	1393713_at	Gmds	7.7389	0.01244959	1374743_at	RGD1565362_predicted	10.8517	0.00000032
1370252_at	"Avpi1"	" -14.7370"	"0.00000027"	1374599_at	Herc1_predicted	7.7358	0.01252027	1373377_at	Scnm1_predicted	-10.879	0.00000032
1386945_a_at	"Prkab1"	" 14.7279"	"0.00000027"	1376715_at	Cbara1	7.7264	0.0127345	1388816_at	Dlgh1	10.8378	0.00000033
1391345_at	"Bmper_predicted"	" 14.7215"	"0.00000027"	1383926_at	Bub1b	7.7218	0.01284074	1379719_at	NA	10.8251	0.00000033
1397268_at	"LOC679784"	" 14.6892"	"0.00000029"	1390237_at	Timm8a	-7.7181	0.01292701	1380483_at	Pde8b	10.8302	0.00000033
1389759_at	"Celsr1"	" -14.6617"	"0.00000029"	1384076_at	Cachd1_predicted	7.7127	0.01305474	1373271_at	RGD1563971_predicted	-10.82	0.00000033
1384002_at	"LOC361929"	" 14.6355"	"0.00000030"	1390022_at	Arpc5	-7.7068	0.01319499	1398837_at	Tceb2	-10.839	0.00000033
1377653_at	"LOC366669"	" 14.6227"	"0.00000031"	1393230_s_at	RGD1308772_predicted	7.6927	0.01353663	1378248_at	RGD1562438_predicted	10.7997	0.00000034
1374590_at	"LOC680128"	" 14.6165"	"0.00000031"	1389137_at	Cit	7.6808	0.01383456	1389301_at	LOC686892	10.7526	0.00000035
1387206_at	"B4galt6"	" 14.5942"	"0.00000032"	1387127_at	Atrn	7.6755	0.01396831	1383485_at	Mdm2_predicted	-10.755	0.00000035
1383437_at	"RGD1562478_predicted"	" 14.5800"	"0.00000032"	1369629_at	Adk	7.6277	0.01523833	1383744_at	NA	-10.785	0.00000035
1393793_at	NA	" 14.5551"	"0.00000033"	1372929_at	Kcnma1	7.6197	0.0154638	1390480_at	NA	10.7528	0.00000035
1395991_at	"Rimbp2"	" 14.5402"	"0.00000034"	1368031_at	Nolc1	-7.6091	0.01576433	1374652_at	NA	10.7534	0.00000035
1367738_at	"Unc119"	" -14.5229"	"0.00000035"	1389256_at	NA	7.6004	0.01601711	1374715_at	RGD1309487	10.7684	0.00000035
1384288_at	NA	" 14.5130"	"0.00000035"	1388643_at	Fut8	7.5925	0.01625151	1397781_at	RGD1565498_predicted	10.7754	0.00000035
1387178_a_at	"Cbs"	" 14.5042"	"0.00000036"	1389908_at	NA	7.5834	0.01652276	1373283_at	Slc25a3	-10.786	0.00000035
1375884_at	"MGC105560"	" -14.4666"	"0.00000037"	1368064_a_at	Ddc	7.5815	0.01658103	1392481_at	RGD1560909_predicted	-10.736	0.00000036
1383991_at	"RGD1562754_predicted"	" -14.4650"	"0.00000037"	1374042_at	RGD1310453_predicted	7.5797	0.01663673	1386866_at	Ywhag	-10.748	0.00000036
1369967_at	"Cs"	" -14.4592"	"0.00000038"	1383256_at	Dnttip2_predicted	-7.5722	0.01686765	1372601_at	Atf5	-10.719	0.00000037
1381163_at	NA	" 14.4550"	"0.00000038"	1371999_at	NA	-7.5706	0.01691478	1387753_s_at	Magi3	10.7215	0.00000037
1371363_at	"Gpd1"	" -14.4409"	"0.00000038"	1375888_at	LOC691036	7.552	0.01750255	1391980_at	LOC360990	10.6951	0.00000038
1394077_at	"Rnd3"	" -14.4351"	"0.00000039"	1398981_at	LOC691278	-7.5416	0.01783912	1383910_at	NA	10.6919	0.00000038
1396207_at	"Rdx"	" -14.4296"	"0.00000039"	1370345_at	Ccnb1	7.5399	0.01789565	1374874_at	RGD1561042_predicted	10.6945	0.00000038
1370399_at	"Cyp4b1"	" 14.4061"	"0.00000040"	1390026_at	Bag3	-7.5321	0.0181543	1375186_at	LOC680594	-10.674	0.00000039
1388615_at	"Rap1a"	" -14.3944"	"0.00000041"	1393033_at	Yars2	-7.5302	0.01821622	1389150_at	NA	10.6696	0.00000039
1388368_at	"Med28_predicted"	" -14.3559"	"0.00000043"	1398771_at	Slc3a2	-7.5201	0.01855893	1371719_at	Brd2	-10.64	0.0000004
1368711_at	"Foxa2"	" -14.3475"	"0.00000043"	1376060_at	NA	7.5075	0.0189944	1393585_at	NA	10.6455	0.0000004
1384503_at	"Klk8"	" 14.3302"	"0.00000044"	1370188_at	Sfrs10	-7.493	0.01950739	1367483_at	RGD1309148_predicted	-10.636	0.0000004
1372229_at	"Pdk3"	" 14.2966"	"0.00000046"	1388584_at	NA	-7.4924	0.01952988	1376273_at	Rnasen	10.6437	0.0000004
1373374_at	"Lmo4"	" -14.2913"	"0.00000046"	1368476_at	Nr3c2	7.4895	0.01963648	1374621_at	Taf1c_predicted	-10.64	0.0000004
1369100_at	"Nalp6"	" 14.2751"	"0.00000047"	1382126_at	Ncor1	7.4846	0.01981237	1389608_at	Abcf2_predicted	-10.611	0.00000041
1383061_at	NA	" -14.2747"	"0.00000047"	1391608_at	RGD1565449_predicted	7.4826	0.01988618	1391280_at	H13_predicted	-10.623	0.00000041
1376709_at	"Slc39a8"	" 14.2677"	"0.00000047"	1372479_at	NA	7.4705	0.02033579	1383074_at	Man2a1	10.6084	0.00000041
1367680_at	"Acox1"	" 14.2563"	"0.00000048"	1379386_at	RGD1563164_predicted	-7.4699	0.0203578	1383770_at	NA	10.5885	0.00000041
1385338_at	"LOC690559"	" 14.2377"	"0.00000049"	1386059_at	RGD1562346_predicted	7.4656	0.0205217	1374687_at	NA	10.6124	0.00000041
1389265_at	"Gbe1"	" 14.2157"	"0.00000050"	1385667_x_at	NA	7.4626	0.02063278	1377656_at	Rbm13	-10.61	0.00000041

1372805_at	"RGD1310444_predicted"	" 14.2000"	"0.00000051"	1399000_at	LOC291411	7.4573	0.02083689	1381685_a_at	RGD1305773_predicted	10.5941	0.00000041
1375990_a_at	NA	" 14.1584"	"0.00000054"	1367713_at	Eif2s1	-7.4543	0.02095233	1372043_at	RGD1311709_predicted	-10.62	0.00000041
1383692_at	"LOC681037"	" 14.1428"	"0.00000055"	1390913_at	NA	-7.4538	0.02097293	1374591_at	RGD1561090_predicted	10.6122	0.00000041
1369712_at	"Stk3"	" 14.1273"	"0.00000056"	1372634_at	Adprhl2_predicted	-7.4469	0.02124159	1392465_at	Sap18	-10.625	0.00000041
1385426_at	"RGD1305326_predicted"	" -14.1256"	"0.00000056"	1374638_at	Pex13_predicted	-7.4005	0.02314888	1375521_at	Tceal8	-10.586	0.00000041
1367496_at	"Tm9sf2"	" 14.0706"	"0.00000060"	1375823_at	Slc4a4	7.3969	0.02330423	1379203_at	Tcfdp2_predicted	10.5846	0.00000041
1369871_at	"Areg"	" -14.0189"	"0.00000064"	1377719_a_at	NA	7.3926	0.02348843	1388038_at	Atrn	10.5704	0.00000042
1389113_at	"Slc45a3_predicted"	" -14.0028"	"0.00000065"	1392968_at	NA	7.3911	0.02355705	1380891_at	Gphn	10.5781	0.00000042
1393990_at	"Zfp503_predicted"	" 13.9927"	"0.00000066"	1374857_at	LOC499709	-7.3749	0.02427549	1390648_at	Herc2_predicted	10.561	0.00000042
1397589_at	NA	" 13.9712"	"0.00000068"	1386866_at	Ywhag	-7.3728	0.0243718	1373870_at	RGD1305486	-10.567	0.00000042
1373754_at	"LOC680692"	" -13.9695"	"0.00000068"	1371663_at	RGD1305138_predicted	-7.3685	0.02456542	1381983_at	RGD1307927_predicted	10.5587	0.00000042
1368082_at	"Slc4a2"	" 13.9424"	"0.00000070"	1398342_at	LOC501052	-7.3579	0.02505255	1383626_at	RGD1562173_predicted	-10.564	0.00000042
1386695_at	NA	" 13.9246"	"0.00000072"	1388424_at	Eif3s1_predicted	-7.3552	0.02517922	1390178_at	Srprb	-10.562	0.00000042
1393760_at	"LOC690654"	" 13.9174"	"0.00000073"	1368268_at	Tdg	-7.3531	0.02528063	1370976_at	G3bp	-10.541	0.00000043
1372755_at	"Mal2"	" 13.9011"	"0.00000074"	1370684_s_at	LOC501546	7.3497	0.02543937	1389388_at	NA	10.5476	0.00000043
1374440_at	"Dhrs8"	" 13.8370"	"0.00000080"	1375418_at	Trrap_predicted	7.3257	0.02660622	1392770_at	Neo1	10.5378	0.00000043
1398664_at	"Gramd3"	" 13.8265"	"0.00000081"	1369979_at	Scap2	7.3234	0.0267169	1370438_at	Capon	10.5261	0.00000044
1387408_at	"Siah2"	" 13.7866"	"0.00000085"	1386466_at	NA	-7.3218	0.02679638	1367808_at	Timm8b	-10.519	0.00000044
1382183_at	NA	" 13.7758"	"0.00000087"	1378543_at	Hnrpa2b1_predicted	-7.3186	0.02695798	1373634_at	NA	10.4944	0.00000045
1368471_at	"Guca2a"	" 13.7395"	"0.00000091"	1398496_at	NA	-7.3123	0.02727536	1378457_at	NA	10.4969	0.00000045
1368244_at	"As3mt"	" 13.7368"	"0.00000091"	1372066_at	RGD1310022	-7.3121	0.02728852	1383640_at	NA	10.507	0.00000045
1375428_at	"Creg_predicted"	" -13.7312"	"0.00000092"	1368936_at	Txnl1	-7.3024	0.02778725	1388508_at	RGD1561287_predicted	-10.497	0.00000045
1368540_at	"Tpbg"	" -13.7291"	"0.00000092"	1378153_at	NA	7.2981	0.02801164	1388819_at	Scamp1	10.5009	0.00000045
1370202_at	"Hrasls3"	" -13.7172"	"0.00000093"	1373272_at	Plekha5	7.295	0.02817373	1371791_at	Surf4	-10.499	0.00000045
1375954_at	"S100a13_predicted"	" -13.6905"	"0.00000096"	1376416_at	RGD1309461	7.2912	0.02837584	1391169_at	RGD1562562_predicted	10.4765	0.00000046
1393582_at	NA	" 13.6714"	"0.00000099"	1368889_at	Vti1a	7.2879	0.02854825	1392972_at	Trio	10.4721	0.00000046
1381994_at	"Aebp2_predicted"	" -13.6678"	"0.00000099"	1398976_at	Ncor1	7.2873	0.02858165	1371868_at	Bcas2_predicted	-10.444	0.00000047
1376589_at	"LOC683708"	" 13.6531"	"0.00000101"	1375974_at	NA	-7.2766	0.02916159	1372651_at	Cdw92	10.4441	0.00000047
1369536_at	"Edn2"	" 13.6457"	"0.00000102"	1396831_at	Glrp1_predicted	7.2674	0.02966831	1373719_at	Map4k3	10.4466	0.00000047
1386908_at	"Glrx1"	" 13.6410"	"0.00000103"	1373924_at	RGD1306568	7.2511	0.03058967	1371498_at	MGC125271	-10.45	0.00000047
1373215_at	"Abr_predicted"	" 13.6140"	"0.00000106"	1395488_at	Msh3	7.2456	0.03090556	1388825_at	NA	10.4208	0.00000048
1380360_at	"Oma1_predicted"	" 13.6132"	"0.00000106"	1383146_at	RGD1562629_predicted	7.2367	0.0314258	1389562_at	NA	10.4356	0.00000048
1367957_at	"Rgs3"	" -13.6030"	"0.00000108"	1373377_at	Scnm1_predicted	-7.2326	0.03166956	1389180_at	NA	10.4376	0.00000048
1368945_at	"Bmp2"	" -13.5978"	"0.00000108"	1370190_at	H3f3b	-7.2312	0.03175393	1377011_at	RGD1307034_predicted	10.4273	0.00000048
1392374_at	"Ccnd2"	" -13.5974"	"0.00000108"	1375686_at	Ppil3	-7.2269	0.03200712	1371454_at	Tmem93_predicted	-10.42	0.00000048
1380465_at	NA	" -13.5863"	"0.00000110"	1378056_at	Gmnn_predicted	-7.2265	0.03203542	1397556_at	Mak3_predicted	-10.393	0.0000005
1373559_at	NA	" -13.5688"	"0.00000112"	1373017_at	Suclg2	7.2226	0.03226788	1388667_at	Man2a1	10.3888	0.0000005
1394451_at	"Anxa1"	" -13.5268"	"0.00000119"	1379967_at	Zfp367	-7.2132	0.03284586	1380890_at	RGD1307055_predicted	10.3864	0.0000005
1379250_at	"Galm"	" 13.4797"	"0.00000126"	1383253_at	NA	-7.2118	0.03292926	1376240_at	RGD1566399_predicted	10.3683	0.00000051
1368512_a_at	"Enpep"	" -13.4653"	"0.00000128"	1372516_at	Kif22	7.1893	0.03435728	1378685_at	LOC689836	10.3343	0.00000053
1368163_at	"Dpp4"	" -13.4536"	"0.00000130"	1378943_at	RGD1566072_predicted	7.1774	0.03513432	1392492_at	NA	10.3344	0.00000053
1389320_at	"LOC364637"	" 13.4301"	"0.00000134"	1380890_at	RGD1307055_predicted	7.1666	0.03585875	1390475_at	NA	10.3389	0.00000053
1374189_at	"Zfp219"	" -13.4297"	"0.00000134"	1383063_a_at	NA	7.1593	0.0363537	1390132_at	NA	10.3393	0.00000053
1373047_at	"Prkci"	" 13.4228"	"0.00000135"	1373551_at	RGD1309762_predicted	7.1494	0.03703836	1387823_at	Plrg1	-10.348	0.00000053
1392694_at	"RGD1562344_predicted"	" 13.4070"	"0.00000138"	1377837_at	Znf183	-7.1238	0.03888117	1389472_at	RGD1311526_predicted	-10.337	0.00000053
1370870_at	"Me1"	" 13.4054"	"0.00000138"	1377651_at	Trio	7.1123	0.03973128	1383231_at	Snip1	-10.327	0.00000053
1388678_at	"Rnf138"	" -13.3701"	"0.00000145"	1373411_at	Eif1b_predicted	-7.11	0.03991038	1389128_at	Wdfy3_predicted	10.3275	0.00000053
1384315_at	"Pdk3"	" 13.3563"	"0.00000147"	1395132_at	Utrn	7.0907	0.04139618	1379832_at	Polr2d_predicted	-10.313	0.00000054
1372064_at	"Cxcl16"	" -13.3562"	"0.00000147"	1376686_at	Tmtc2_predicted	7.0886	0.0415618	1388723_at	Bre	10.3058	0.00000055
1384039_at	"RGD1310950_predicted"	" -13.3554"	"0.00000147"	1398819_at	Dnaja1	-7.0802	0.04223249	1374078_at	RGD1560612_predicted	10.3044	0.00000055

1372682_at	"RGD1307218"	" 13.3522"	"0.00000148"
1398980_at	"Supt16h_predicted"	" -13.3341"	"0.00000152"
1370195_at	"Snap23"	" -13.3216"	"0.00000154"
1367838_at	"Cth"	" 13.3176"	"0.00000155"
1386720_at	"Tmprss8"	" 13.3142"	"0.00000155"
1377199_at	NA	" 13.3127"	"0.00000156"
1370282_at	"Csrp2"	" 13.3111"	"0.00000156"
1384774_at	"Tmprss8"	" 13.3003"	"0.00000158"
1373352_at	"LOC685385"	" -13.2827"	"0.00000162"
1370631_at	"Reg3g"	" 13.2826"	"0.00000162"
1368007_at	"Dmbt1"	" 13.2804"	"0.00000162"
1383188_at	NA	" 13.2708"	"0.00000164"
1370071_at	"Ada"	" -13.2690"	"0.00000165"
1390026_at	"Bag3"	" -13.2557"	"0.00000168"
1378505_at	NA	" 13.2498"	"0.00000169"
1376924_a_at	"Palmd"	" 13.2416"	"0.00000171"
1389670_at	"RGD1566402_predicted"	" 13.2035"	"0.00000179"
1378739_at	"LOC500292"	" 13.1997"	"0.00000180"
1370387_at	"Cyp3a9"	" -13.1978"	"0.00000181"
1392888_at	"Gpc4"	" -13.1971"	"0.00000181"
1368973_at	"Adar"	" -13.1708"	"0.00000187"
1376520_at	NA	" 13.1599"	"0.00000190"
1384236_at	NA	" 13.1587"	"0.00000190"
1377603_at	"Snx24"	" 13.1423"	"0.00000194"
1394340_at	"Inpp1"	" 13.1350"	"0.00000196"
1370200_at	"Glud1"	" 13.1006"	"0.00000205"
1377181_at	NA	" -13.0784"	"0.00000211"
1367791_at	"Ramp1"	" 13.0726"	"0.00000213"
1367905_at	"Enpp3"	" -13.0676"	"0.00000214"
1380170_at	"Gabarapl2"	" -13.0674"	"0.00000214"
1390944_at	"MGC108776"	" 13.0645"	"0.00000215"
1390789_at	"Acad11_predicted"	" 13.0508"	"0.00000219"
1368651_at	"Pklr"	" 13.0218"	"0.00000227"
1389647_at	"Snx13_predicted"	" 13.0178"	"0.00000228"
1389609_at	"Tm7sf3"	" 12.9999"	"0.00000234"
1387631_at	"Hpgd"	" 12.9938"	"0.00000236"
1379467_at	NA	" -12.9726"	"0.00000242"
1372569_at	"Fhl3_predicted"	" 12.9691"	"0.00000243"
1381206_at	NA	" 12.9574"	"0.00000247"
1390146_at	NA	" -12.9547"	"0.00000248"
1384775_s_at	"Tmprss8"	" 12.9437"	"0.00000252"
1380695_at	NA	" -12.9240"	"0.00000258"
1373240_at	"Dhrs3"	" 12.9198"	"0.00000260"
1367987_at	"Rnpep"	" -12.9002"	"0.00000266"
1367614_at	"Anxa1"	" -12.9001"	"0.00000266"
1388454_at	"RGD1562933_predicted"	" 12.8799"	"0.00000274"
1395794_at	"Tpm1"	" -12.8748"	"0.00000276"
1382579_at	"Tnrc9_predicted"	" 12.8510"	"0.00000284"
1376191_at	"Hpgd"	" 12.8405"	"0.00000288"
1373649_at	"LOC691853"	" 12.8382"	"0.00000289"

1372619_at	Mrpl49	-7.0793	0.04230166
1368943_at	Rnase4	-7.0792	0.04231227
1372498_at	Ciapin1	-7.0779	0.04241245
1388990_at	Mki67ip	-7.0759	0.04257359
1375562_at	NA	7.0752	0.04263409
1390137_at	Traf4af1	7.0634	0.04359459
1372643_at	RGD1563977_predicted	7.0621	0.0437063
1389265_at	Gbe1	7.0596	0.04391724
1379668_at	RGD1564725_predicted	-7.0428	0.04534227
1389703_at	Zzef1_predicted	7.0415	0.04544968
1383415_at	Ppp3ca	7.0413	0.04546667
1373632_at	Taf9	-7.0246	0.04693765
1373036_at	RGD1561455_predicted	7.0217	0.04720374
1390856_at	NA	7.0176	0.04757011
1373898_at	LOC360760	7.0157	0.04774192
1372821_at	RGD1565969_predicted	7.0153	0.04778205
1379669_at	NA	7.0152	0.04778726
1367972_at	Cand1	-7.0081	0.04843789
1388437_at	NA	6.9933	0.04982966

1376562_at	RGD1561817_predicted	10.3041	0.00000055
1373696_at	RGD1564859_predicted	10.3004	0.00000055
1379971_at	Zc3h6_predicted	10.2951	0.00000055
1390818_at	NA	10.28	0.00000056
1384135_at	NA	10.2813	0.00000056
1382330_at	Ddef2_predicted	10.2328	0.00000058
1389450_at	LOC368084	-10.243	0.00000058
1371608_at	Mrps34_predicted	-10.259	0.00000058
1398039_at	NA	10.2339	0.00000058
1379363_at	NA	10.2369	0.00000058
1373941_at	NA	10.2376	0.00000058
1391577_at	Pgam5	-10.236	0.00000058
1377949_s_at	RGD1306359_predicted	10.2517	0.00000058
1391880_at	Tpd52_predicted	10.2386	0.00000058
1389035_at	Yipf3	-10.251	0.00000058
1383064_at	NA	10.2257	0.00000059
1387018_at	Argbp2	10.2098	0.0000006
1392514_at	Bxdc1_predicted	-10.211	0.0000006
1373393_at	LOC299907	10.21	0.0000006
1376619_at	RGD1561090_predicted	10.2092	0.0000006
1380182_at	RGD1563437_predicted	10.2063	0.0000006
1378198_at	Ophn1_predicted	10.1936	0.00000061
1388770_at	RGD1304890	-10.175	0.00000062
1371891_at	RGD1308430_predicted	-10.181	0.00000062
1383001_at	RGD1560812_predicted	-10.183	0.00000062
1380100_at	RGD1561817_predicted	10.1756	0.00000062
1377199_at	NA	10.1537	0.00000064
1386962_at	Plcb4	10.1497	0.00000064
1370366_at	Timm10	-10.145	0.00000064
1377755_at	Ascc3_predicted	10.133	0.00000065
1376098_a_at	Lad1_predicted	-10.134	0.00000065
1395999_at	NA	10.1323	0.00000065
1389131_at	Nt5c3l	-10.129	0.00000065
1371718_at	Sra1	-10.126	0.00000065
1375846_at	Xpr1_predicted	10.1382	0.00000065
1398811_at	Jtb	-10.109	0.00000066
1393057_at	LOC308320	-10.11	0.00000066
1367972_at	Cand1	-10.099	0.00000067
1374190_at	Clybl	10.1024	0.00000067
1383710_at	LOC362683	10.0955	0.00000067
1379785_at	NA	-10.103	0.00000067
1387950_at	Nip7	-10.091	0.00000067
1373479_at	Ppp3ca	10.0895	0.00000067
1367624_at	Atf4	-10.065	0.00000068
1383278_at	Centg2_predicted	10.0788	0.00000068
1398803_at	Dync1h1	10.07	0.00000068
1385128_at	NA	10.0564	0.00000068
1394290_at	NA	10.0625	0.00000068
1381336_at	NA	10.0638	0.00000068
1390387_at	NA	10.0657	0.00000068

1379910_at	"RGD1561967_predicted"	" -12.7954"	"0.00000306"
1368446_at	"Spink1"	" -12.7708"	"0.00000316"
1392045_at	"Tmem22"	" 12.7654"	"0.00000319"
1397375_at	"Acsl5"	" -12.7472"	"0.00000326"
1387690_at	"Casp3"	" 12.7232"	"0.00000337"
1387542_at	"Slc9a3"	" 12.6914"	"0.00000352"
1381858_at	NA	" 12.6856"	"0.00000354"
1373017_at	"Suclg2"	" 12.6749"	"0.00000359"
1382761_at	"Pde7a"	" -12.6703"	"0.00000362"
1399125_at	"Inpp1"	" 12.6623"	"0.00000365"
1373497_at	NA	" 12.6612"	"0.00000366"
1388738_at	"RGD1563485_predicted"	" 12.6567"	"0.00000368"
1378147_at	"RGD1561878_predicted"	" 12.6350"	"0.00000379"
1385739_at	NA	" 12.6308"	"0.00000381"
1389443_at	NA	" -12.6120"	"0.00000391"
1370834_at	"Hs3st1"	" -12.6036"	"0.00000395"
1380397_at	NA	" 12.5873"	"0.00000404"
1394132_at	"MGC72974"	" 12.5868"	"0.00000404"
1376066_at	NA	" -12.5799"	"0.00000408"
1367933_at	"Amd1"	" -12.5711"	"0.00000413"
1392894_at	"Fgl2"	" 12.5624"	"0.00000418"
1393170_at	"RGD1561431_predicted"	" 12.5569"	"0.00000421"
1375522_at	"Nmt1"	" -12.5557"	"0.00000422"
1393837_at	"RGD1565966_predicted"	" -12.5523"	"0.00000424"
1373780_at	"Tspan1"	" -12.5420"	"0.00000430"
1389703_at	"Zzef1_predicted"	" 12.5279"	"0.00000438"
1371557_at	"Thap4"	" 12.5246"	"0.00000440"
1379023_at	"Txndc12"	" -12.5016"	"0.00000454"
1389364_at	"Ndfip2_predicted"	" 12.4902"	"0.00000461"
1371520_at	"Znf291"	" 12.4863"	"0.00000463"
1384958_at	NA	" -12.4855"	"0.00000464"
1386916_at	"Aco1"	" 12.4816"	"0.00000466"
1393412_at	"Fam3c"	" 12.4791"	"0.00000468"
1394562_at	"Ptprm"	" -12.4762"	"0.00000470"
1368008_at	"Prom1"	" 12.4637"	"0.00000478"
1370237_at	"Hadhsc"	" 12.4438"	"0.00000491"
1376022_at	NA	" -12.4373"	"0.00000495"
1372149_at	"Auh_predicted"	" 12.4247"	"0.00000503"
1383697_at	NA	" -12.4103"	"0.00000513"
1376102_at	"Tmbim1"	" -12.4069"	"0.00000516"
1373616_at	"Slk"	" -12.3759"	"0.00000538"
1368164_at	"Blvra"	" 12.3726"	"0.00000540"
1371662_at	"Kars"	" -12.3657"	"0.00000546"
1379416_at	NA	" 12.3643"	"0.00000547"
1387270_at	"Hhex"	" -12.3594"	"0.00000550"
1387982_at	"Tlr4"	" -12.3466"	"0.00000560"
1392799_at	"RGD1561254_predicted"	" -12.3298"	"0.00000573"
1390553_at	NA	" -12.3072"	"0.00000591"
1367687_a_at	"Pam"	" -12.2931"	"0.00000602"
1392946_at	NA	" 12.2817"	"0.00000612"

1374967_at	NA	10.0695	0.00000068
1375358_at	NA	10.0791	0.00000068
1390711_at	NA	10.0795	0.00000068
1372813_at	RGD1306682_predicted	-10.08	0.00000068
1380309_at	RGD1309443_predicted	10.067	0.00000068
1387059_at	Stk39	10.0757	0.00000068
1373578_at	Trim2	10.0666	0.00000068
1390300_at	NA	10.0493	0.00000069
1376337_at	Smarca2	10.0437	0.00000069
1398866_at	Magi3	10.0329	0.0000007
1388794_at	Rbmxrt_predicted	-10.023	0.00000071
1376035_at	Ttc7b_predicted	10.0196	0.00000071
1374349_at	Ctdspl_predicted	10.0151	0.00000072
1377359_at	LOC501665	-10.017	0.00000072
1375414_at	Taf9	-10.012	0.00000072
1383931_at	Ptprk	9.9979	0.00000073
1379945_at	RGD1563166_predicted	-10.003	0.00000073
1386916_at	Aco1	9.9747	0.00000075
1369676_at	Itpr2	9.9813	0.00000075
1392916_at	Mtap7_predicted	9.9784	0.00000075
1367849_at	Sdc1	-9.9701	0.00000075
1387369_at	Exoc6	9.9593	0.00000076
1368476_at	Nr3c2	9.9553	0.00000076
1398810_at	Pdap1	-9.9585	0.00000076
1372614_at	RGD1311849_predicted	9.9586	0.00000076
1368944_at	Dlgh1	9.9305	0.00000078
1391415_at	LOC289809	-9.9358	0.00000078
1398935_at	LOC683687	9.9299	0.00000078
1372257_at	NA	9.9393	0.00000078
1373145_at	RGD1560511_predicted	9.932	0.00000078
1368321_at	Egr1	-9.9231	0.00000079
1374135_at	Ipo4_predicted	-9.9169	0.00000079
1372457_at	Mtus1	9.9282	0.00000079
1373464_at	NA	9.9261	0.00000079
1390873_at	Btbd11_predicted	9.9074	0.0000008
1368311_at	Mgmt	9.9021	0.0000008
1379244_at	NA	9.9078	0.0000008
1395325_s_at	Tmem32_predicted	-9.9136	0.0000008
1374070_at	Gpx2	-9.8898	0.00000081
1378791_at	Map3k4_predicted	9.8912	0.00000081
1389703_at	Zzef1_predicted	9.8948	0.00000081
1368032_at	Nolc1	-9.8812	0.00000082
1372059_at	RGD1309437	9.8799	0.00000082
1373430_at	Baz2b_predicted	9.858	0.00000084
1370317_at	LOC245960	-9.8623	0.00000084
1373069_at	Mrps30_predicted	-9.8528	0.00000084
1379269_at	NA	9.8608	0.00000084
1399034_at	Pcnx	9.8597	0.00000084
1393626_at	Sorl1_predicted	9.8524	0.00000084
1373962_at	Usp32_predicted	9.8613	0.00000084

1374310_at	"Ppm1j"	" -12.2812"	"0.00000612"
1373957_at	"Reln"	" 12.2757"	"0.00000617"
1378315_at	NA	" 12.2645"	"0.00000627"
1393319_a_at	"RGD1562478_predicted"	" 12.2444"	"0.00000644"
1384965_at	"Slk"	" -12.2379"	"0.00000650"
1378321_at	"Rassf4"	" 12.2164"	"0.00000669"
1377825_at	NA	" 12.2148"	"0.00000671"
1368593_at	"Cd1d1"	" -12.2103"	"0.00000675"
1390125_at	"Tm9sf1"	" 12.1996"	"0.00000685"
1390228_at	NA	" -12.1867"	"0.00000697"
1378197_at	"KIFC2"	" 12.1769"	"0.00000707"
1371527_at	"Emp1"	" 12.1649"	"0.00000719"
1398378_at	"Gstk1"	" 12.1626"	"0.00000721"
1374035_at	"Rem2"	" -12.1577"	"0.00000726"
1372095_at	"RGD1309821_predicted"	" 12.1525"	"0.00000731"
1369717_at	"Nmu"	" -12.1514"	"0.00000732"
1370211_at	"Nrgn"	" -12.1508"	"0.00000733"
1368838_at	"Tpm4"	" -12.1351"	"0.00000749"
1379390_at	"St6galnac2"	" 12.1173"	"0.00000768"
1389199_at	NA	" 12.1097"	"0.00000776"
1371791_at	"Surf4"	" -12.1015"	"0.00000785"
1369896_s_at	"Rbm16"	" -12.0747"	"0.00000814"
1370810_at	"Ccnd2"	" -12.0734"	"0.00000816"
1369518_at	"Pik3r3"	" -12.0729"	"0.00000816"
1381974_at	"Btbd3_predicted"	" 12.0716"	"0.00000818"
1367735_at	"Acadl"	" 12.0636"	"0.00000827"
1371004_at	"Sort1"	" -12.0572"	"0.00000834"
1384770_at	NA	" 12.0562"	"0.00000836"
1393114_at	"siat7D"	" -12.0478"	"0.00000845"
1372329_at	"RGD1311435"	" 12.0446"	"0.00000849"
1393039_a_at	"RGD1562478_predicted"	" 12.0428"	"0.00000851"
1383900_at	"RGD1562974_predicted"	" -12.0374"	"0.00000858"
1373357_at	NA	" 12.0212"	"0.00000877"
1384069_at	"Lrp11_predicted"	" 12.0166"	"0.00000883"
1391282_at	"RGD1306962_predicted"	" 12.0117"	"0.00000889"
1389862_at	"RGD1305327"	" -12.0061"	"0.00000896"
1373226_at	NA	" -11.9994"	"0.00000904"
1392948_at	"Clic6"	" -11.9953"	"0.00000909"
1381403_at	"Me2_predicted"	" -11.9873"	"0.00000920"
1382211_at	NA	" -11.9757"	"0.00000935"
1376159_at	"RGD1562321_predicted"	" -11.9440"	"0.00000977"
1371351_at	"MGC112727"	" -11.9284"	"0.00000999"
1373536_at	NA	" 11.9239"	"0.00001005"
1383212_at	NA	" 11.9197"	"0.00001011"
1370219_at	"Cyba"	" -11.9060"	"0.00001031"
1395707_at	NA	" 11.9045"	"0.00001033"
1379282_at	"Lrrfip2"	" 11.8973"	"0.00001043"
1369249_at	"Ank"	" 11.8931"	"0.00001050"
1379117_at	NA	" 11.8522"	"0.00001112"
1383964_at	"Ankrd50_predicted"	" 11.8359"	"0.00001138"

1390406_at	Arhgap18_predicted	9.8399	0.00000085
1389291_at	Chchd3_predicted	9.844	0.00000085
1373898_at	LOC360760	9.8444	0.00000085
1379997_at	NA	9.8468	0.00000085
1372069_at	Ankrd15	9.8234	0.00000086
1377945_at	Ddx18	-9.8274	0.00000086
1391238_at	Gmds	9.8368	0.00000086
1375895_at	NA	-9.8241	0.00000086
1384835_at	NA	9.8222	0.00000086
1384504_at	NA	9.834	0.00000086
1372744_at	Pkp4_predicted	9.8294	0.00000086
1390392_at	RGD1309602_predicted	-9.8282	0.00000086
1391478_at	Znf532_predicted	9.8243	0.00000086
1367713_at	Eif2s1	-9.8096	0.00000087
1390561_at	Lig4_predicted	-9.8092	0.00000087
1383752_at	Nol1_predicted	-9.8131	0.00000087
1373036_at	RGD1561455_predicted	9.8144	0.00000087
1372121_at	Usp9x_predicted	9.8031	0.00000088
1371726_at	NA	9.7906	0.00000089
1374410_at	NA	9.7944	0.00000089
1373105_at	Tmed1	-9.7906	0.00000089
1375995_at	RGD1304592_predicted	9.7804	0.0000009
1387925_at	Asns	-9.7724	0.00000091
1398231_at	NA	9.7682	0.00000091
1371572_at	App	9.763	0.00000092
1388953_at	Gnl3	-9.7565	0.00000092
1391474_at	RGD1560170_predicted	9.7548	0.00000092
1376608_at	RGD1565135_predicted	9.7625	0.00000092
1386882_at	Tctex1	-9.765	0.00000092
1389316_at	Usp9x_predicted	9.748	0.00000093
1384198_at	Cova1_predicted	9.7377	0.00000094
1372787_at	Crlz1	-9.7336	0.00000094
1375901_at	Ddx21b	-9.7326	0.00000094
1379999_at	Mical3	9.7332	0.00000094
1390515_at	NA	9.7289	0.00000094
1385857_at	Ophn1_predicted	9.7369	0.00000094
1390237_at	Timm8a	-9.7393	0.00000094
1389355_at	Ier5	-9.712	0.00000096
1388668_at	Mettl2_predicted	-9.7116	0.00000096
1370303_at	Slc35a4	-9.7107	0.00000096
1388560_at	Wdr77	-9.7195	0.00000096
1377387_a_at	NA	-9.7075	0.00000097
1393106_x_at	Gtf2f1	-9.6906	0.00000099
1368994_a_at	Garnl1	9.678	0.000001
1375140_at	LOC686892	9.6845	0.000001
1383378_at	NA	9.6794	0.000001
1374326_at	Ppan	-9.6806	0.000001
1373273_at	Prpf38a_predicted	-9.6787	0.000001
1383055_at	Gnaq	9.6678	0.00000101
1374746_at	LOC500877	-9.6643	0.00000101

1382253_at	"RGD1307173"	" -11.8258"	"0.00001154"
1372399_at	"RGD1307832_predicted"	" -11.7904"	"0.00001213"
1388126_at	"Minpp1"	" -11.7902"	"0.00001213"
1371899_at	"Prkra"	" 11.7879"	"0.00001217"
1386970_at	"Eif2b4"	" -11.7606"	"0.00001266"
1373412_at	"Nt5c3_predicted"	" -11.7547"	"0.00001276"
1389873_at	"Pycard"	" 11.7375"	"0.00001308"
1383687_at	NA	" 11.7318"	"0.00001318"
1380388_at	NA	" 11.7300"	"0.00001322"
1393992_at	"Hoxa7"	" 11.7268"	"0.00001328"
1373173_at	NA	" -11.7251"	"0.00001331"
1375728_at	"Entpd4_predicted"	" -11.6991"	"0.00001381"
1373036_at	"RGD1561455_predicted"	" 11.6888"	"0.00001401"
1394731_at	"Csnk1g3"	" -11.6858"	"0.00001407"
1378481_at	NA	" 11.6828"	"0.00001413"
1385513_at	NA	" 11.6478"	"0.00001486"
1395410_at	"Ppp2r3a"	" 11.6163"	"0.00001554"
1379632_at	"RGD1308774"	" 11.6161"	"0.00001555"
1383920_at	"Amt"	" 11.6159"	"0.00001555"
1370507_at	"Dlgap4"	" -11.5896"	"0.00001615"
1370699_a_at	"Egfr"	" 11.5886"	"0.00001617"
1396126_at	NA	" -11.5853"	"0.00001625"
1387952_a_at	"Cd44"	" 11.5720"	"0.00001656"
1371653_at	"Tpm4"	" -11.5682"	"0.00001665"
1368910_at	"Ppm2c"	" -11.5679"	"0.00001666"
1382629_at	"Mlstd2"	" 11.5673"	"0.00001667"
1397410_at	"RGD1564895_predicted"	" -11.5583"	"0.00001689"
1395268_at	"Wwp1"	" 11.5372"	"0.00001741"
1374935_at	NA	" 11.5294"	"0.00001761"
1387239_a_at	"Padi4"	" -11.5221"	"0.00001780"
1388748_at	"Laptm4a"	" -11.5162"	"0.00001795"
1388830_at	"Pkn2"	" 11.5072"	"0.00001818"
1382995_at	"Nrp2"	" 11.5062"	"0.00001821"
1378288_at	"RGD1311100_predicted"	" 11.4967"	"0.00001846"
1375061_at	NA	" -11.4892"	"0.00001866"
1369799_at	"Abat"	" -11.4797"	"0.00001892"
1388432_at	"Optn"	" 11.4785"	"0.00001895"
1391433_at	"Mte1"	" -11.4682"	"0.00001923"
1371103_at	"Rab6a"	" -11.4619"	"0.00001941"
1395980_at	"RGD1311640_predicted"	" 11.4600"	"0.00001946"
1373462_at	"Eed_predicted"	" -11.4160"	"0.00002075"
1383970_at	NA	" 11.4082"	"0.00002098"
1378791_at	"Map3k4_predicted"	" 11.4069"	"0.00002102"
1395532_at	"LOC688018"	" -11.4028"	"0.00002115"
1367734_at	"Akr1b4"	" 11.4023"	"0.00002116"
1381390_at	"RGD1564833_predicted"	" 11.3944"	"0.00002141"
1372809_at	"LOC290595"	" 11.3891"	"0.00002157"
1389118_s_at	"LOC685385"	" -11.3843"	"0.00002172"
1388909_at	"Oxnad1_predicted"	" 11.3756"	"0.00002200"
1372099_at	"LOC297530"	" 11.3736"	"0.00002206"

1393089_at	LOC690035	9.6733	0.00000101
1377019_at	NA	-9.6582	0.00000101
1391916_at	NA	9.6657	0.00000101
1383194_a_at	NA	9.6671	0.00000101
1383604_at	NA	9.67	0.00000101
1379386_at	RGD1563164_predicted	-9.6656	0.00000101
1380969_at	RGD1564670_predicted	9.6643	0.00000101
1375685_at	Tcfcp2l1_predicted	9.6614	0.00000101
1376100_at	Tubb6	-9.6535	0.00000101
1367693_at	Ywhah	-9.6576	0.00000101
1383719_at	Mtap7_predicted	9.6438	0.00000103
1389279_at	NA	-9.6399	0.00000103
1398473_at	Bloc1s2	-9.6328	0.00000104
1386755_at	LOC312863	9.6316	0.00000104
1371820_at	Mesdc2	-9.6346	0.00000104
1371822_at	Polr3d	-9.63	0.00000104
1399013_at	RGD1310905_predicted	-9.6362	0.00000104
1368070_at	Stx8	9.6207	0.00000105
1388159_at	NA	9.6167	0.00000106
1392088_at	Pde8b	9.6091	0.00000107
1373741_at	Pus1	-9.6055	0.00000107
1367787_at	Ica1	9.5978	0.00000108
1381145_at	NA	9.6016	0.00000108
1374481_at	Cno	-9.5902	0.00000109
1378359_at	RGD1563950_predicted	-9.584	0.0000011
1372679_at	Unc45a	-9.5849	0.0000011
1373738_at	LOC686999	-9.5771	0.00000111
1390194_at	NA	9.5724	0.00000111
1386918_a_at	Oprs1	-9.5684	0.00000112
1371663_at	RGD1305138_predicted	-9.558	0.00000113
1372143_at	Ube2v2	-9.5584	0.00000113
1399056_at	LOC365592	9.5508	0.00000114
1376639_at	Rnf126	-9.5531	0.00000114
1389510_at	Lyar	-9.5378	0.00000116
1393143_at	NA	9.5303	0.00000116
1372544_at	NA	9.534	0.00000116
1372176_at	Prkca	9.5356	0.00000116
1373516_at	RGD1305633_predicted	-9.5383	0.00000116
1376681_at	RGD1308302	-9.5402	0.00000116
1383461_at	RGD1311362	-9.541	0.00000116
1371571_at	App	9.5243	0.00000117
1388400_at	Cdc34_predicted	-9.521	0.00000117
1390856_at	NA	9.5277	0.00000117
1367833_at	Psmc5	-9.5218	0.00000117
1389245_at	Psmd7_predicted	-9.517	0.00000117
1371622_at	RGD1564623_predicted	-9.5157	0.00000117
1397596_at	Trim2	9.5239	0.00000117
1392983_at	Psmd12	-9.5122	0.00000118
1368083_at	Ccnh	-9.5037	0.00000119
1390692_at	Ctps_predicted	-9.5057	0.00000119

1388848_at	"RGD1308350"	" -11.3634"	"0.00002239"
1370194_at	"Snap23"	" -11.3550"	"0.00002267"
1369953_a_at	"Cd24"	" -11.3169"	"0.00002396"
1369747_at	"Nat2"	" 11.3163"	"0.00002399"
1369976_at	"Dynll1"	" -11.3071"	"0.00002431"
1374846_at	"Clp1"	" -11.3026"	"0.00002447"
1382235_at	"RGD1306809_predicted"	" 11.2995"	"0.00002458"
1394761_at	NA	" 11.2938"	"0.00002479"
1372920_at	"LOC680409"	" 11.2833"	"0.00002517"
1398395_at	"Itgb1bp1_predicted"	" -11.2746"	"0.00002549"
1394740_at	NA	" 11.2725"	"0.00002557"
AFFX_Rat_Hexok	"Hk1"	" -11.2640"	"0.00002589"
1384361_at	"Mal2"	" 11.2634"	"0.00002591"
1377034_at	"Serpinb1a"	" 11.2624"	"0.00002595"
1376804_at	"RGD1560646"	" 11.2598"	"0.00002605"
1385432_at	"LOC683713"	" 11.2587"	"0.00002609"
1371393_at	"Clstn1"	" 11.2579"	"0.00002613"
1382218_at	"RGD1305807"	" 11.2550"	"0.00002624"
1377854_at	"RGD1305647_predicted"	" -11.2529"	"0.00002632"
1391817_at	NA	" -11.2491"	"0.00002646"
1379803_at	"Lmo4"	" -11.2430"	"0.00002670"
1372816_at	NA	" 11.2382"	"0.00002689"
1399069_at	"RGD1310351_predicted"	" 11.2361"	"0.00002697"
1382592_at	"RGD1306908"	" 11.2356"	"0.00002699"
1392349_at	"Slc5a3"	" -11.2196"	"0.00002764"
1374315_at	NA	" 11.2181"	"0.00002770"
1388310_at	"Sui1-rs1_predicted"	" -11.2138"	"0.00002787"
1376989_at	NA	" -11.2017"	"0.00002837"
1373578_at	"Trim2"	" 11.1904"	"0.00002885"
1385269_s_at	"LOC304743"	" -11.1854"	"0.00002906"
1385054_at	NA	" -11.1706"	"0.00002971"
1377359_at	"LOC501665"	" -11.1703"	"0.00002972"
1383128_at	"Rpl5"	" -11.1679"	"0.00002982"
1378305_at	"Tm4sf1_predicted"	" -11.1624"	"0.00003006"
1367702_at	"Acadm"	" 11.1624"	"0.00003006"
1378284_at	NA	" -11.1597"	"0.00003018"
1380577_at	"Abcg2"	" 11.1549"	"0.00003040"
1387769_a_at	"Id3"	" -11.1472"	"0.00003075"
1374146_at	"Mad2l2"	" 11.1405"	"0.00003105"
1371797_at	"Cad"	" -11.1236"	"0.00003184"
1372019_at	"RGD1310128_predicted"	" -11.1229"	"0.00003187"
1367709_at	"Cd63"	" -11.1182"	"0.00003209"
1392534_at	"Tmepai_predicted"	" -11.1162"	"0.00003219"
1370901_at	"RGD1306215_predicted"	" -11.1162"	"0.00003219"
1391099_at	NA	" -11.1073"	"0.00003262"
1377666_at	"Chdh"	" 11.1041"	"0.00003277"
1369944_at	"Marcksl1"	" -11.0959"	"0.00003317"
1379739_at	NA	" -11.0945"	"0.00003324"
1373392_at	"Tparl"	" -11.0925"	"0.00003334"
1390437_at	NA	" 11.0844"	"0.00003374"

1384475_at	Plekha5	9.5034	0.00000119
1379575_at	Rere	9.499	0.00000119
1383250_at	Utp14a	-9.5038	0.00000119
1383606_at	Mtac2d1	9.4897	0.00000121
1374180_at	NA	-9.4894	0.00000121
1368117_at	Gphn	9.4773	0.00000122
1377751_at	NA	9.4752	0.00000122
1371455_at	Pmm1	-9.4758	0.00000122
1372496_at	RGD1561264_predicted	-9.4753	0.00000122
1394849_at	Zbtb20_predicted	9.4818	0.00000122
1396254_at	Ncoa2	9.4705	0.00000123
1373666_at	Rapgef5	9.4694	0.00000123
1374289_at	Rpo1-1	-9.4719	0.00000123
1383256_at	Dnttip2_predicted	-9.4603	0.00000124
1388424_at	Eif3s1_predicted	-9.4591	0.00000124
1376235_at	NA	9.4576	0.00000124
1367742_at	Cpt1b	-9.4536	0.00000125
1372990_at	Creb3	-9.4446	0.00000126
1389265_at	Gbe1	9.4399	0.00000126
1368355_at	Myo5b	9.4482	0.00000126
1384518_at	RGD1563764_predicted	9.4398	0.00000126
1372821_at	RGD1565969_predicted	9.4479	0.00000126
1393364_at	Slc12a8	9.442	0.00000126
1379264_at	Znrf1_predicted	9.4434	0.00000126
1370245_at	Ctsl	-9.4251	0.00000128
1373462_at	Eed_predicted	-9.4214	0.00000128
1390452_at	Epb4.1l1	9.422	0.00000128
1372362_at	Galk2_predicted	9.4299	0.00000128
1389108_at	LOC363309	9.4265	0.00000128
1389869_at	LOC689397	-9.4258	0.00000128
1373497_at	NA	9.4228	0.00000128
1374066_at	Cdc2l6_predicted	9.4165	0.00000129
1395654_at	NA	-9.4135	0.00000129
1374128_at	NA	9.4146	0.00000129
1376001_at	Praf1_predicted	-9.4121	0.00000129
1376465_at	RGD1563869_predicted	9.4131	0.00000129
1379668_at	RGD1564725_predicted	-9.4171	0.00000129
1372701_at	Hspca	-9.4072	0.0000013
1368391_at	Slc7a1	-9.4037	0.0000013
1383548_at	Ankrd49_predicted	-9.3987	0.00000131
1389569_at	Bxdc2	-9.4008	0.00000131
1375972_at	RGD1307493_predicted	-9.3986	0.00000131
1390885_at	NA	9.3902	0.00000132
1383123_at	NA	9.3934	0.00000132
1394564_at	NA	-9.3831	0.00000133
1383652_at	LOC502872	-9.3794	0.00000134
1376833_at	RGD1304793_predicted	-9.3755	0.00000134
1398948_at	Tax1bp1	9.3715	0.00000135
1376576_at	Dusp11	-9.3634	0.00000136
1391607_at	RGD1566319_predicted	-9.368	0.00000136

1389110_at	"Pprf18"	" -11.0713"	"0.00003441"
1389124_at	"LOC690586"	" 11.0699"	"0.00003448"
1390659_at	NA	" 11.0647"	"0.00003474"
1390782_at	"Clec14a"	" -11.0617"	"0.00003490"
1372433_at	"RGD1310211_predicted"	" -11.0606"	"0.00003496"
1369986_at	"Hagh"	" 11.0518"	"0.00003542"
1397011_at	NA	" 11.0501"	"0.00003550"
1391928_at	"Gopc_predicted"	" -11.0207"	"0.00003710"
1389514_at	"Lrrn6a"	" -11.0129"	"0.00003753"
1374591_at	"RGD1561090_predicted"	" 11.0120"	"0.00003758"
1368052_at	"Tspan8"	" 11.0029"	"0.00003809"
1367995_at	"Cat"	" 10.9999"	"0.00003826"
1372093_at	"Mxi1"	" 10.9986"	"0.00003833"
1393575_at	NA	" -10.9817"	"0.00003932"
1395620_at	"RGD1306302"	" -10.9769"	"0.00003960"
1371509_at	"Tbrg1"	" -10.9690"	"0.00004007"
1377858_at	"Prdm2"	" -10.9684"	"0.00004010"
1389349_s_at	"Il17re"	" 10.9591"	"0.00004067"
1379393_at	"Vil1_predicted"	" 10.9577"	"0.00004075"
1370067_at	"Me1"	" 10.9454"	"0.00004151"
1380346_at	"Serpinb1a"	" 10.9441"	"0.00004159"
1385211_at	"RGD1310951_predicted"	" 10.9383"	"0.00004195"
1391765_at	"Lrrc48"	" 10.9370"	"0.00004204"
1377720_x_at	NA	" 10.9344"	"0.00004220"
1375918_at	"LOC497967"	" -10.9333"	"0.00004227"
1371414_at	"Gsn"	" -10.9290"	"0.00004254"
1390555_at	"RGD1564914_predicted"	" -10.9281"	"0.00004260"
1367815_at	"Slc5a6"	" -10.9202"	"0.00004311"
1373207_at	NA	" 10.9198"	"0.00004313"
1395998_at	"Nol5a"	" -10.9001"	"0.00004442"
1389466_at	"LOC691155"	" -10.8996"	"0.00004446"
1379824_at	"Tox_predicted"	" 10.8989"	"0.00004451"
1397596_at	"Trim2"	" 10.8877"	"0.00004527"
1382975_at	"Ceacam1"	" -10.8758"	"0.00004608"
1396144_at	"RGD1561817_predicted"	" 10.8729"	"0.00004628"
1387084_at	"Dpp4"	" -10.8698"	"0.00004650"
1368991_at	"Smpd3"	" -10.8495"	"0.00004795"
1388988_at	"Abhd14b"	" 10.8479"	"0.00004806"
1369259_at	"Dio1"	" -10.8455"	"0.00004823"
1376792_at	"RGD1559797_predicted"	" 10.8341"	"0.00004907"
1398231_at	NA	" 10.8294"	"0.00004942"
1393314_at	NA	" 10.8264"	"0.00004964"
1383493_at	NA	" -10.8209"	"0.00005006"
1376060_at	NA	" 10.8195"	"0.00005016"
1373060_at	"RGD1306781_predicted"	" 10.8157"	"0.00005045"
1368533_at	"Heph"	" 10.8150"	"0.00005050"
1387670_at	"Gpd2"	" -10.7950"	"0.00005206"
1394597_at	"Ddhd1"	" -10.7920"	"0.00005230"
1385268_at	"LOC304743"	" -10.7820"	"0.00005309"
1368123_at	"Igf1r"	" 10.7729"	"0.00005382"

1380030_at	Znf593_predicted	-9.3618	0.00000136
1373876_at	Eif4e2_predicted	-9.3564	0.00000137
1385739_at	NA	9.3596	0.00000137
1371419_at	Spnb2	9.3554	0.00000137
1377007_at	NA	9.3441	0.00000139
1377197_at	NA	9.3467	0.00000139
1397748_at	NA	-9.3416	0.0000014
1377850_at	Znrf1_predicted	9.3359	0.00000141
1371818_at	NA	-9.3291	0.00000142
1379980_at	NA	9.3273	0.00000142
1375663_at	Ube2f	-9.3258	0.00000142
1398900_at	Dctn3_predicted	-9.32	0.00000143
1377794_at	NA	-9.322	0.00000143
1384794_at	NA	-9.3151	0.00000143
1379475_at	RGD1563120_predicted	9.3225	0.00000143
1368012_at	Tep1	9.3164	0.00000143
1371843_at	Yipf5	-9.3215	0.00000143
1387462_at	Chrm3	9.3067	0.00000144
1372999_at	Dcun1d5	-9.3099	0.00000144
1388576_at	Eif3s9	-9.3126	0.00000144
1372748_at	Tbc1d2b	9.3068	0.00000144
1370017_at	Emd	-9.2992	0.00000145
1367479_at	LOC691534	-9.2999	0.00000145
1371967_at	Mrpl16	-9.3043	0.00000145
1376071_at	NA	9.2979	0.00000145
1390320_at	Centg2_predicted	9.294	0.00000146
1371921_at	Catna1	9.2846	0.00000147
1380688_at	Galntl4_predicted	9.2893	0.00000147
1388849_at	LOC680423	-9.2867	0.00000147
1389310_at	NA	9.2857	0.00000147
1367790_at	Snd1	9.2897	0.00000147
1396019_at	NA	9.2771	0.00000148
1387875_at	Ptk2	9.2786	0.00000148
1388870_at	RGD1560397_predicted	9.2812	0.00000148
1368955_at	Cask	9.2661	0.0000015
1371908_at	Nxt1_predicted	-9.2699	0.0000015
1390208_at	Htatip2_predicted	-9.2563	0.00000151
1383561_at	Lig4_predicted	-9.2567	0.00000151
1375551_at	MGC94720	-9.2617	0.00000151
1383511_at	NA	9.2559	0.00000151
1392973_at	Nav2	9.2576	0.00000151
1376804_at	RGD1560646_predicted	9.2584	0.00000151
1386906_a_at	40057	9.2474	0.00000152
1378634_at	Hdac8_predicted	9.2481	0.00000152
1367636_at	Igf2r	9.25	0.00000152
1379307_at	Sap1	9.2485	0.00000152
1388369_at	Tmed9	-9.2506	0.00000152
1391461_at	RGD1306576_predicted	-9.2435	0.00000153
1376797_at	Csrp2bp_predicted	-9.235	0.00000155
1374025_at	Nmnat3	9.2363	0.00000155

1388345_at	NA	" -10.7699"	"0.00005407"
1378057_at	"Flrt3_predicted"	" 10.7630"	"0.00005464"
1367898_at	"Bnip3l"	" 10.7565"	"0.00005518"
1374604_at	NA	" 10.7552"	"0.00005529"
1370036_at	"Suox"	" -10.7538"	"0.00005541"
1375337_at	"Adam9"	" -10.7470"	"0.00005598"
1395595_at	"Mki67ip"	" -10.7426"	"0.00005636"
1367705_at	"Glrx1"	" 10.7409"	"0.00005651"
1383222_at	"LOC257646"	" -10.7341"	"0.00005709"
1398839_at	"Txn1"	" 10.7305"	"0.00005741"
1383574_at	"Man1a_predicted"	" 10.7266"	"0.00005774"
1374031_at	"RGD1310937_predicted"	" -10.7107"	"0.00005916"
1378675_at	"RGD1561831_predicted"	" -10.7082"	"0.00005938"
1382429_at	NA	" 10.7028"	"0.00005987"
1383616_at	"RGD1560373_predicted"	" 10.6985"	"0.00006026"
1369310_at	"Basp1"	" 10.6983"	"0.00006029"
1373984_at	NA	" 10.6944"	"0.00006064"
1373377_at	"Scnm1_predicted"	" -10.6915"	"0.00006092"
1382344_at	NA	" -10.6842"	"0.00006159"
1380960_at	"Coro2a"	" 10.6833"	"0.00006168"
1367824_at	"Fnta"	" -10.6797"	"0.00006202"
1388039_a_at	"Gabbr1"	" -10.6792"	"0.00006206"
1385581_at	NA	" 10.6757"	"0.00006239"
1381229_at	"Prdm2"	" -10.6624"	"0.00006367"
1371559_at	NA	" 10.6520"	"0.00006470"
1389654_at	"Pls1_predicted"	" 10.6516"	"0.00006473"
1373413_at	"RGD1310481_predicted"	" 10.6398"	"0.00006592"
1374471_at	"LOC498972"	" -10.6397"	"0.00006592"
1390193_at	NA	" 10.6393"	"0.00006596"
1386280_at	"Mettl7b"	" 10.6270"	"0.00006722"
1371997_at	"Akr1e1"	" 10.6052"	"0.00006950"
1367693_at	"Ywhah"	" -10.5961"	"0.00007048"
1393048_at	"Adra2a"	" 10.5928"	"0.00007083"
1374758_at	NA	" 10.5887"	"0.00007128"
1399137_at	NA	" -10.5784"	"0.00007242"
1390201_at	"Rap1a"	" -10.5713"	"0.00007321"
1372779_at	"B3gnt1_predicted"	" -10.5683"	"0.00007355"
1370245_at	"Ctsl"	" -10.5618"	"0.00007429"
1383833_at	"LOC500991"	" -10.5542"	"0.00007516"
1370182_at	"Ptprn2"	" 10.5412"	"0.00007668"
1388429_at	"LOC499391"	" -10.5398"	"0.00007684"
1368748_at	"Tesk2"	" -10.5205"	"0.00007916"
1387886_at	"Prelp"	" -10.5176"	"0.00007952"
1387780_at	"Dnaja2"	" -10.5090"	"0.00008057"
1372463_at	"Fcho2_predicted"	" 10.5047"	"0.00008111"
1383905_at	"Zfp216_predicted"	" -10.5024"	"0.00008140"
1368063_a_at	"Yt521"	" -10.5024"	"0.00008141"
1389883_at	"RGD1563224_predicted"	" 10.5020"	"0.00008145"
1381381_at	"Mnab_predicted"	" -10.5014"	"0.00008153"
1389399_at	"RGD1562218"	" -10.4998"	"0.00008174"

1385043_at	RGD1565362_predicted	9.2278	0.00000156
1383283_at	Ocrl	9.2252	0.00000157
1373619_at	Ankrd10	9.2168	0.00000158
1398793_at	Cdc5l	-9.2174	0.00000158
1376692_at	Hipk2_predicted	9.2166	0.00000158
1392498_at	LOC691318	9.2172	0.00000158
1379322_at	Psmd7_predicted	-9.2165	0.00000158
1384728_at	Ches1_predicted	9.2121	0.00000159
1387782_at	Dynll2	-9.21	0.00000159
1371386_at	RGD1306643_predicted	9.2099	0.00000159
1380319_at	Tex10_predicted	-9.2066	0.00000159
1372556_at	LOC502374	-9.1971	0.00000161
1383695_at	Vipr1	9.2002	0.00000161
1370912_at	Hspa1b	-9.1894	0.00000162
1368249_at	Klf15	9.1935	0.00000162
1386586_at	MGC125015	-9.1917	0.00000162
1390867_at	NA	-9.187	0.00000162
1374197_at	NA	9.1915	0.00000162
1398392_at	NA	9.1918	0.00000162
1376678_at	RGD1562438_predicted	9.1904	0.00000162
1372221_at	NA	9.1814	0.00000163
1392980_at	Tiam1	9.1774	0.00000164
1398606_at	Golph4	9.1634	0.00000168
1367736_at	Rraga	-9.1604	0.00000168
1367715_at	Tnfrsf1a	-9.1618	0.00000168
1374312_at	Uck1_predicted	-9.1622	0.00000168
1390977_at	St7l	9.1524	0.0000017
1388907_at	RGD1306053	-9.1456	0.00000171
1397854_at	NA	9.1437	0.00000172
1377457_a_at	Sorl1_predicted	9.1412	0.00000172
1386379_at	NA	9.1223	0.00000177
1368818_at	Psme4	9.1205	0.00000177
1373958_at	LOC367902	-9.1174	0.00000178
1375432_at	LOC679140	-9.1175	0.00000178
1371569_at	NA	9.1145	0.00000178
1390351_at	NA	9.1186	0.00000178
1393713_at	Gmds	9.1114	0.00000179
1393661_at	NA	9.11	0.00000179
1398450_at	NA	9.1113	0.00000179
1378740_at	Rasal2_predicted	9.1092	0.00000179
1393615_at	RGD1561030_predicted	9.104	0.0000018
1388131_at	Tubb2b	-9.0984	0.00000181
1395544_at	NA	-9.0963	0.00000182
1389760_at	Pcgf6	-9.0952	0.00000182
1385798_at	NA	9.0855	0.00000184
1371202_a_at	Nfib	9.0799	0.00000186
1399080_at	LOC688495	-9.0669	0.00000189
1384239_at	LOC680133	9.0646	0.0000019
1372075_at	LOC362264	-9.0608	0.00000191
1368889_at	Vti1a	9.0615	0.00000191

1371468_at	"LOC312502"	" -10.4939"	"0.00008249"
1378134_at	"Atp8b1_predicted"	" 10.4924"	"0.00008268"
1367974_at	"Anxa3"	" -10.4910"	"0.00008285"
1392789_at	"LOC501039"	" -10.4871"	"0.00008335"
1389602_at	NA	" 10.4827"	"0.00008392"
1367701_at	"Ramp2"	" -10.4801"	"0.00008426"
1376715_at	"Cbara1"	" 10.4795"	"0.00008434"
1386721_at	"Zfp503_predicted"	" 10.4720"	"0.00008531"
1385676_at	"Cd2bp2_predicted"	" -10.4708"	"0.00008548"
1392488_at	"RGD1307915_predicted"	" -10.4705"	"0.00008552"
1376132_at	"LOC362129"	" 10.4687"	"0.00008575"
1373064_at	"Dnajc15_predicted"	" 10.4602"	"0.00008690"
1379987_at	"RGD1304595_predicted"	" -10.4594"	"0.00008700"
1383689_at	"B4galt4"	" -10.4567"	"0.00008737"
1388159_at	NA	" 10.4557"	"0.00008750"
1390520_at	"LOC680216"	" 10.4503"	"0.00008824"
1387448_at	"Bet1l"	" -10.4479"	"0.00008856"
1377102_at	"Tmem63a_predicted"	" -10.4378"	"0.00008996"
1389207_at	"Egln1"	" 10.4365"	"0.00009015"
1375685_at	"Tcfcp2l1_predicted"	" 10.4357"	"0.00009025"
1382909_at	NA	" -10.4311"	"0.00009090"
1379877_at	"Zfp406_predicted"	" 10.4274"	"0.00009142"
1374191_at	"Rhbdf1"	" 10.4269"	"0.00009149"
1398811_at	"Jtb"	" -10.4203"	"0.00009243"
1383839_at	"Spg20"	" -10.4181"	"0.00009275"
1370251_at	"Avpi1"	" -10.4114"	"0.00009372"
1393661_at	NA	" 10.4099"	"0.00009394"
1394228_at	NA	" 10.4086"	"0.00009413"
1393452_at	"Car9_predicted"	" 10.4045"	"0.00009474"
1393201_at	"RGD1562062_predicted"	" -10.4044"	"0.00009474"
1394522_at	NA	" -10.4025"	"0.00009503"
1395663_at	"Mall"	" 10.4013"	"0.00009520"
1376619_at	"RGD1561090_predicted"	" 10.3995"	"0.00009547"
1385072_at	"Galm"	" 10.3988"	"0.00009557"
1374486_at	"Arhgef11"	" 10.3899"	"0.00009690"
1382351_at	"Gem_predicted"	" 10.3879"	"0.00009721"
1374095_at	"Slc4a8"	" 10.3733"	"0.00009945"
1374594_at	"LOC363060"	" 10.3656"	"0.00010064"
1393747_at	"Spink4"	" 10.3617"	"0.00010126"
1391707_at	"LOC691923"	" 10.3616"	"0.00010127"
1388584_at	NA	" -10.3602"	"0.00010149"
1383952_at	"Mical1_predicted"	" -10.3507"	"0.00010301"
1376200_at	"MGC72974"	" 10.3401"	"0.00010472"
1371002_at	"Pdcd2"	" -10.3400"	"0.00010475"
1372845_at	"Rpp21"	" -10.3397"	"0.00010479"
1380249_at	NA	" -10.3348"	"0.00010559"
1393184_at	NA	" -10.3125"	"0.00010935"
1373132_at	"LOC691898"	" -10.3057"	"0.00011052"
1370506_at	"Coq7"	" 10.3036"	"0.00011088"
1389941_at	"Arl2bp"	" -10.2974"	"0.00011196"

1370325_at	Gorasp2	-9.0567	0.00000192
1389384_at	Hrpap20	-9.0545	0.00000192
1398952_at	RGD1564093_predicted	-9.0524	0.00000192
1398299_at	Arhgef11	9.047	0.00000193
1374466_at	NA	9.048	0.00000193
1379312_at	Pprc1_predicted	-9.0482	0.00000193
1371024_at	Cutl1	9.0384	0.00000196
1392421_at	Baz2b_predicted	9.0316	0.00000197
1372392_at	NA	9.0309	0.00000197
1378467_at	NA	9.0348	0.00000197
1373030_at	NA	-9.0281	0.00000198
1390289_at	RGD1311375_predicted	9.0257	0.00000198
1396901_at	RGD1566319_predicted	-9.0254	0.00000198
1396955_at	NA	-9.0218	0.00000199
1390949_at	NA	9.0232	0.00000199
1373047_at	Prkci	9.0203	0.00000199
1377651_at	Trio	9.0183	0.00000199
1373027_at	NA	9.0136	0.00000201
1388992_at	Ep400	9.0065	0.00000202
1382126_at	Ncor1	9.0072	0.00000202
1376135_at	Dars2	9.001	0.00000203
1398496_at	NA	-9.0045	0.00000203
1386059_at	RGD1562346_predicted	9.0049	0.00000203
1372138_at	Cutl1	8.9972	0.00000204
1389200_at	Bysl	-8.9949	0.00000205
1398572_at	LOC679672	-8.9948	0.00000205
1392909_at	NA	8.9936	0.00000205
1382188_at	RGD1311086	-8.9938	0.00000205
1376291_at	NA	-8.9852	0.00000207
1374486_at	Arhgef11	8.9795	0.00000208
1396105_at	LOC688133	8.981	0.00000208
1373372_at	LOC501282	-8.976	0.00000209
1371610_at	Tnks_predicted	8.977	0.00000209
1372764_at	LOC689601	-8.9558	0.00000216
1376274_at	Btbd10	-8.9528	0.00000217
1374397_at	Eif2s2	-8.9497	0.00000217
1373597_at	Pop7_predicted	-8.9512	0.00000217
1377884_at	RGD1560155_predicted	8.9469	0.00000218
1373200_at	Eef1e1_predicted	-8.9428	0.00000219
1386569_at	LOC294560	8.9341	0.00000222
1388117_at	Snrpb	-8.9322	0.00000222
1373682_at	Ddx51_predicted	-8.927	0.00000224
1367590_at	Ran	-8.9248	0.00000224
1392662_at	RGD1566242_predicted	-8.9277	0.00000224
1392744_at	Atg7	8.9193	0.00000225
1371955_at	Mrpl35_predicted	-8.9208	0.00000225
1392471_at	NA	8.919	0.00000225
1392044_at	NA	8.9149	0.00000226
1389908_at	NA	8.9149	0.00000226
1376578_at	Ehmt1_predicted	8.9081	0.00000227

1375452_at	"Mgea6_predicted"	" 10.2892"	"0.00011340"
1385701_at	NA	" -10.2811"	"0.00011485"
1383688_at	NA	" -10.2783"	"0.00011537"
1370831_at	"Mgll"	" 10.2696"	"0.00011695"
1371857_at	"Kctd10"	" -10.2628"	"0.00011820"
1381525_at	"Enah"	" -10.2567"	"0.00011933"
1373763_at	"Zfp297b"	" -10.2531"	"0.00012001"
1394681_at	"Akr1cl1_predicted"	" -10.2444"	"0.00012167"
1382964_at	NA	" 10.2440"	"0.00012174"
1369979_at	"Scap2"	" 10.2414"	"0.00012224"
1378402_at	"Tfcp2l4_predicted"	" -10.2378"	"0.00012293"
1370465_at	"Abcb1a"	" 10.2335"	"0.00012376"
1388577_at	NA	" -10.2153"	"0.00012737"
1373570_at	"Npepl1_predicted"	" 10.2133"	"0.00012775"
1373027_at	NA	" 10.2051"	"0.00012942"
1371485_at	NA	" -10.2001"	"0.00013046"
1382439_at	"Itgb6"	" 10.1994"	"0.00013059"
1377994_at	NA	" -10.1971"	"0.00013106"
1372123_at	"Sdhb_predicted"	" 10.1925"	"0.00013202"
1390951_at	NA	" 10.1887"	"0.00013281"
1368834_at	"Camk2d"	" 10.1855"	"0.00013349"
1370244_at	"Ctsl"	" -10.1808"	"0.00013447"
1386999_at	"Ywhab"	" -10.1794"	"0.00013477"
1367551_a_at	"RGD1306410"	" 10.1739"	"0.00013595"
1370464_at	"Abcb1a"	" 10.1670"	"0.00013744"
1388311_at	"Mrfap1"	" -10.1648"	"0.00013792"
1390832_at	NA	" 10.1624"	"0.00013844"
1387770_at	"Ifi27l"	" -10.1611"	"0.00013873"
1380975_at	NA	" 10.1569"	"0.00013966"
1398799_at	"Eif4e"	" -10.1463"	"0.00014201"
1388809_at	"Smpdl3a"	" -10.1285"	"0.00014608"
1387521_at	"Pdcd4"	" -10.1212"	"0.00014778"
1391333_at	"Lrrfip2"	" 10.1193"	"0.00014821"
1389792_at	"Solh_predicted"	" -10.1026"	"0.00015220"
1387218_at	"Tff3"	" -10.1006"	"0.00015268"
1371705_at	"Vps26"	" -10.0935"	"0.00015441"
1368513_at	"Enpep"	" -10.0865"	"0.00015615"
1393364_at	"Slc12a8"	" 10.0844"	"0.00015665"
1382271_at	"Rps6ka5_predicted"	" 10.0816"	"0.00015737"
1372744_at	"Pkp4_predicted"	" 10.0753"	"0.00015893"
1393912_at	NA	" -10.0739"	"0.00015930"
1371392_at	"Gpi"	" 10.0736"	"0.00015937"
1373283_at	"Slc25a3"	" -10.0616"	"0.00016245"
1370298_at	"Zfp99"	" 10.0595"	"0.00016299"
1388780_at	"Terf2ip"	" -10.0547"	"0.00016423"
1374341_at	"Thap7"	" -10.0512"	"0.00016515"
1387078_at	"Inpp4a"	" -10.0507"	"0.00016528"
1368349_at	"Fgfbp1"	" -10.0498"	"0.00016553"
1390382_at	"RGD1311457_predicted"	" -10.0483"	"0.00016591"
1397146_at	NA	" 10.0468"	"0.00016631"

1388709_at	LOC362703	-8.9095	0.00000227
1373162_at	LOC681708	-8.9108	0.00000227
1384149_at	RGD1311424_predicted	-8.9128	0.00000227
1379488_at	Trp53rk_predicted	-8.9086	0.00000227
1368050_at	Ccnl1	-8.8997	0.0000023
1370280_at	Hprt	-8.8951	0.00000232
1395645_at	Sipa1l1	-8.8923	0.00000232
1392274_at	Pdgfc	8.8905	0.00000233
1389277_at	RGD1306148_predicted	8.8855	0.00000234
1379778_at	RGD1307983_predicted	-8.887	0.00000234
1374464_at	Tfg	-8.885	0.00000234
1389262_at	NA	8.8786	0.00000236
1368072_at	Btg3	-8.8713	0.00000238
1393933_at	Sorl1_predicted	8.8713	0.00000238
1367803_at	Nup54	-8.8672	0.00000239
1393605_at	RGD1565941_predicted	8.8684	0.00000239
1385342_at	Twistnb_predicted	-8.8682	0.00000239
1377842_at	Twistnb_predicted	-8.8666	0.00000239
1371390_at	Tubb2c	-8.8633	0.0000024
1374694_at	RGD1559931_predicted	8.8613	0.00000241
1396834_at	Braf	-8.8568	0.00000242
1382143_at	Farp1_predicted	8.8557	0.00000242
1391017_at	Farp1_predicted	8.851	0.00000243
1383498_at	NA	8.8532	0.00000243
1381103_a_at	RGD1562579_predicted	-8.8478	0.00000244
1390960_at	NA	-8.8405	0.00000247
1378029_at	NA	8.8391	0.00000247
1387097_at	Fut2	-8.8366	0.00000248
1373558_at	NA	8.8301	0.0000025
1378181_at	Rpp40	-8.8286	0.00000251
1399014_at	Vps4a	-8.8282	0.00000251
1386647_at	Mtap7_predicted	8.8239	0.00000252
1390517_at	NA	8.8249	0.00000252
1387025_at	Dync1i1	8.8212	0.00000253
1383942_at	LOC683687	8.8129	0.00000255
1389327_at	Mrpl32_predicted	-8.8123	0.00000255
1374194_at	Tmem16c_predicted	8.8127	0.00000255
1393458_s_at	RGD1563764_predicted	8.8056	0.00000258
1383689_at	B4galt4	-8.801	0.00000259
1374922_at	Atpbd1b_predicted	-8.8001	0.0000026
1398930_at	Atp6v0b_predicted	-8.7943	0.00000262
1373539_at	NA	-8.7922	0.00000262
1388437_at	NA	8.7926	0.00000262
1387411_at	Ptprk	8.7916	0.00000262
1379250_at	Galm	8.7878	0.00000263
1373643_at	NA	8.7873	0.00000263
1394568_at	RGD1563365_predicted	-8.7869	0.00000263
1374376_at	Rgnef_predicted	8.7832	0.00000264
1388999_at	NA	8.78	0.00000265
1389655_at	RGD1306819	-8.7817	0.00000265

1388581_at	"Hn1"	" -10.0454"	"0.00016669"
1371785_at	"Tnfrsf12a"	" -10.0353"	"0.00016940"
1399114_at	"Gtf2e2_predicted"	" -10.0323"	"0.00017021"
1367761_at	"Ndel1"	" -10.0256"	"0.00017205"
1379041_at	"Pik3ca"	" -10.0215"	"0.00017316"
1383307_at	"MGC114379"	" -10.0111"	"0.00017605"
1371611_at	"Ext2_predicted"	" 10.0106"	"0.00017619"
1369940_at	"Taldo1"	" 10.0057"	"0.00017760"
1389613_at	NA	" 9.9992"	"0.00017946"
1367499_at	"Slc35c1_predicted"	" 9.9940"	"0.00018096"
1383511_at	NA	" 9.9906"	"0.00018194"
1387812_at	"Pcsk6"	" 9.9900"	"0.00018211"
1372067_at	"Txndc1"	" -9.9798"	"0.00018509"
1378508_at	NA	" -9.9761"	"0.00018619"
1392795_at	"Sema6a_predicted"	" -9.9638"	"0.00018990"
1367901_at	"Gusb"	" -9.9624"	"0.00019033"
1388888_at	NA	" -9.9622"	"0.00019039"
1373411_at	"Eif1b_predicted"	" -9.9619"	"0.00019048"
1367803_at	"Nup54"	" -9.9607"	"0.00019085"
1384835_at	NA	" 9.9412"	"0.00019692"
1373209_at	NA	" -9.9411"	"0.00019693"
1372066_at	"RGD1310022"	" -9.9405"	"0.00019713"
1391582_at	NA	" -9.9402"	"0.00019722"
1393101_at	"Fbxl10"	" -9.9251"	"0.00020207"
1371544_at	"LOC681415"	" -9.9236"	"0.00020255"
1383283_at	"Ocrl"	" 9.9164"	"0.00020492"
1374806_at	"Sfn_predicted"	" -9.9149"	"0.00020543"
1376135_at	"Dars2"	" 9.9100"	"0.00020705"
1377281_at	"Supt16h_predicted"	" -9.9000"	"0.00021040"
1381006_at	"Hgfac"	" -9.9000"	"0.00021041"
1399034_at	"Pcnx"	" 9.8973"	"0.00021130"
1388534_at	"Cdc26"	" 9.8899"	"0.00021384"
1368021_at	"Adh1"	" -9.8854"	"0.00021540"
1388979_at	"Smndc1"	" -9.8832"	"0.00021618"
1389529_at	NA	" 9.8819"	"0.00021663"
1387310_at	"Atp2c2"	" -9.8767"	"0.00021846"
1392863_at	"Flrt3_predicted"	" 9.8757"	"0.00021881"
1372158_at	"Lrp16"	" 9.8613"	"0.00022395"
1379656_a_at	NA	" 9.8568"	"0.00022556"
1397107_at	"RGD1559968_predicted"	" 9.8535"	"0.00022677"
1392603_at	NA	" 9.8506"	"0.00022786"
1389550_at	"Sh3gl2"	" 9.8452"	"0.00022984"
1390765_at	"Wfdc2"	" -9.8446"	"0.00023006"
1371521_at	"LOC683381"	" -9.8405"	"0.00023160"
1370181_at	"Rab4a"	" 9.8380"	"0.00023254"
1376686_at	"Tmtc2_predicted"	" 9.8307"	"0.00023528"
1384980_at	NA	" -9.8264"	"0.00023692"
1375944_at	"Acss2_predicted"	" -9.8201"	"0.00023936"
1372707_at	"Rab6a"	" -9.8094"	"0.00024355"
1396643_at	"Capon"	" -9.8056"	"0.00024506"

1372093_at	Mxi1	8.7763	0.00000266
1371823_at	Stag2_predicted	8.7743	0.00000267
1388810_at	Abce1	-8.7701	0.00000268
1387969_at	Cxcl10	-8.7688	0.00000268
1388874_at	Mtss1_predicted	8.7698	0.00000268
1367488_at	RGD1562348_predicted	8.7654	0.0000027
1367711_at	Psmc2	-8.7615	0.00000271
1390838_at	NA	8.7565	0.00000272
1390468_at	NA	8.7572	0.00000272
1392653_at	NA	8.759	0.00000272
1397570_at	LOC498295	8.7532	0.00000273
1367514_at	LOC361635	8.7515	0.00000274
1388587_at	Ier3	-8.744	0.00000276
1380857_at	NA	-8.7441	0.00000276
1390149_at	Tacc2	8.7442	0.00000276
1390338_at	LOC361646	8.7407	0.00000277
1390290_at	Surf6_predicted	-8.7409	0.00000277
1368924_at	Ghr	8.7376	0.00000278
1390893_at	Tmem69	-8.7334	0.0000028
1383675_at	NA	-8.7297	0.00000281
1371851_at	Psmd6	-8.7297	0.00000281
1376584_at	NA	8.7274	0.00000282
1390232_at	RGD1311946	-8.7256	0.00000282
1385132_at	NA	-8.7156	0.00000285
1392968_at	NA	8.7161	0.00000285
1381075_at	NA	8.7189	0.00000285
1395642_at	Nol9	-8.717	0.00000285
1376860_at	Nritp	8.7187	0.00000285
1389013_at	RGD1308469_predicted	-8.7155	0.00000285
1388136_at	Timm9	-8.7143	0.00000285
1388568_at	Eif3s7	-8.7045	0.00000289
1382186_a_at	RGD1311086	-8.7017	0.0000029
1371542_at	Tuba4	-8.6955	0.00000293
1372829_at	Rbm8_predicted	-8.6889	0.00000296
1383636_at	NA	8.686	0.00000297
1371893_at	Col4a3bp_predicted	8.6834	0.00000298
1374385_at	RGD1561319_predicted	-8.679	0.000003
1396187_at	Ddx21b	-8.6753	0.00000301
1383627_a_at	Gtf2f1	-8.6771	0.00000301
1390662_at	LOC500084	8.6711	0.00000302
1373845_at	LOC688235	-8.673	0.00000302
1374767_at	RGD1309592	8.6708	0.00000302
1382275_at	MGC125015	-8.6657	0.00000305
1380376_at	NA	8.6552	0.0000031
1382332_at	Stag2_predicted	8.6546	0.0000031
1373054_at	Cdw92	8.6383	0.00000318
1376782_at	Cdc14a_predicted	8.6337	0.00000319
1372732_at	Itpa	-8.634	0.00000319
1378568_a_at	RGD1306228_predicted	-8.6333	0.00000319
1388118_at	Hibadh	8.6322	0.0000032

1379085_at	"RGD1561878_predicted"	"	9.7998"	"0.00024736"
1392417_s_at	"Trp53rk_predicted"	"	-9.7978"	"0.00024815"
1390530_at	NA	"	9.7975"	"0.00024830"
1393481_at	NA	"	9.7953"	"0.00024916"
1382821_at	"Ppfia3"	"	9.7951"	"0.00024927"
1387125_at	"S100a9"	"	-9.7935"	"0.00024988"
1374518_at	"Tmem77"	"	-9.7923"	"0.00025039"
1379810_at	"Scye1"	"	-9.7908"	"0.00025101"
1385182_at	"Pkp1_predicted"	"	-9.7819"	"0.00025465"
1375706_at	NA	"	9.7799"	"0.00025547"
1394772_at	"Tesk2"	"	-9.7783"	"0.00025613"
1380969_at	"RGD1564670_predicted"	"	9.7775"	"0.00025649"
1368967_at	"Eif2b3"	"	-9.7622"	"0.00026294"
1373607_at	"St3gal3"	"	9.7582"	"0.00026464"
1372250_at	"LOC499941"	"	-9.7559"	"0.00026564"
1371886_at	"Crat"	"	9.7471"	"0.00026945"
1373900_at	"Krt2-7"	"	-9.7430"	"0.00027125"
1373719_at	"Map4k3"	"	9.7274"	"0.00027826"
1367504_at	"Fbxo7"	"	9.7140"	"0.00028437"
1399144_at	"LOC498351"	"	-9.7115"	"0.00028553"
1368586_at	"Zg16"	"	9.7073"	"0.00028752"
1379725_at	NA	"	-9.7062"	"0.00028805"
1390019_at	"H3f3b"	"	-9.7038"	"0.00028915"
1382384_at	"MGC114410"	"	9.7011"	"0.00029046"
1388356_at	"S100a16_predicted"	"	-9.7009"	"0.00029056"
1387002_at	"G10"	"	-9.6861"	"0.00029766"
1373786_at	NA	"	9.6832"	"0.00029904"
1379544_at	"LOC689253"	"	9.6820"	"0.00029966"
1368927_at	"Mbc2"	"	-9.6814"	"0.00029996"
1388358_at	"Etfb"	"	9.6807"	"0.00030030"
1395973_at	"RGD1311640_predicted"	"	9.6716"	"0.00030479"
1388456_at	"S100a1"	"	-9.6550"	"0.00031318"
1375368_at	"RGD1307773_predicted"	"	9.6548"	"0.00031329"
1384103_at	"RGD1561416_predicted"	"	-9.6531"	"0.00031413"
1373495_at	"Ube2j1_predicted"	"	-9.6523"	"0.00031458"
1377749_at	NA	"	-9.6484"	"0.00031661"
1397729_x_at	"LOC363060"	"	9.6435"	"0.00031914"
1381445_at	"Esrrg"	"	9.6418"	"0.00032005"
1369890_at	"LOC680174"	"	-9.6414"	"0.00032025"
1398905_at	"Atp6v1g1_predicted"	"	-9.6407"	"0.00032060"
1398857_at	"Surf1"	"	9.6323"	"0.00032504"
1391774_at	NA	"	-9.6320"	"0.00032522"
1374126_at	NA	"	-9.6271"	"0.00032784"
1388786_at	NA	"	-9.6234"	"0.00032983"
1388857_at	"Sec23b_predicted"	"	-9.6128"	"0.00033561"
1377045_at	NA	"	9.6118"	"0.00033618"
1367642_at	"Suclg1"	"	9.6090"	"0.00033770"
1373488_at	NA	"	-9.6064"	"0.00033918"
1371812_at	"LOC362855"	"	9.6057"	"0.00033959"
1375843_at	"Ids"	"	9.6039"	"0.00034058"

1367826_at	Nfe2l2	-8.6304	0.0000032
1395707_at	NA	8.6274	0.00000321
1390271_at	Plekha6_predicted	8.6259	0.00000322
1392991_at	LOC682902	-8.62	0.00000324
1377740_at	NA	8.6222	0.00000324
1370330_at	Sipa1l1	8.6198	0.00000324
1386935_at	Nr4a1	-8.6179	0.00000325
1381644_at	NA	8.615	0.00000326
1373603_at	RGD1565744_predicted	-8.6153	0.00000326
1371835_at	Prkacb	8.6111	0.00000327
1370237_at	Hadhsc	8.6082	0.00000328
1373924_at	RGD1306568	8.6073	0.00000328
1393193_at	Rpusd2_predicted	-8.6093	0.00000328
1379663_at	Cachd1_predicted	8.6	0.00000332
1368031_at	Nolc1	-8.5975	0.00000333
1393478_at	NA	8.5947	0.00000334
1372077_at	Strap	-8.5937	0.00000334
1399088_at	Tlk2_predicted	8.5932	0.00000334
1379396_at	Elmo1_predicted	8.5876	0.00000337
1390576_at	LOC684233	-8.5837	0.00000339
1397255_at	MGC94720	-8.5823	0.00000339
1390382_at	RGD1311457_predicted	-8.5813	0.0000034
1391526_at	Kif16b_predicted	8.5789	0.00000341
1390865_at	LOC681395	8.5753	0.00000341
1389984_at	LOC681740	8.5761	0.00000341
1388917_at	Myo1d	8.578	0.00000341
1379352_at	Pip5k1a	8.5731	0.00000342
1389830_at	Ubtf	-8.5705	0.00000343
1369679_a_at	Nfia	8.5645	0.00000346
1367476_at	Srp14_predicted	-8.5649	0.00000346
1371444_at	Lass2	-8.5587	0.00000349
1368165_at	Prps1	-8.5509	0.00000353
1368725_at	Jag1	-8.5482	0.00000355
1371633_at	Ctnnbl1	8.5458	0.00000356
1398316_at	LOC288913	-8.5451	0.00000356
1389507_at	Nedd4l	8.5441	0.00000356
1389445_at	Zfp688_predicted	-8.5405	0.00000358
1375630_at	MGC72932	-8.5382	0.00000359
1373379_at	Irak1_predicted	-8.5353	0.0000036
1382428_at	Tasp1_predicted	8.5332	0.00000361
1372681_at	NA	8.5279	0.00000363
1368400_at	Timm8a	-8.5288	0.00000363
1374225_at	LOC500700	8.5266	0.00000364
1384377_at	Ddx28_predicted	-8.5172	0.00000368
1389061_at	Nsun5_predicted	-8.5193	0.00000368
1392099_at	Plcl3_predicted	8.5167	0.00000368
1372678_at	RGD1311283_predicted	-8.5186	0.00000368
1389765_at	Nle1_predicted	-8.5153	0.00000369
1383238_at	Qtrt1	-8.5149	0.00000369
1398977_at	Ide	8.5103	0.00000371

1373381_at	"Herc4"	" 9.6037"	"0.00034070"
1372583_at	NA	" -9.5991"	"0.00034329"
1383626_at	"RGD1562173_predicted"	" -9.5972"	"0.00034432"
1387823_at	"Plrg1"	" -9.5947"	"0.00034578"
1379450_at	"Cttnbp2nl_predicted"	" -9.5938"	"0.00034627"
1375815_at	"Ank"	" 9.5898"	"0.00034854"
1372928_at	NA	" 9.5895"	"0.00034872"
1374621_at	"Taf1c"	" -9.5857"	"0.00035093"
1376187_at	"Slc35d1_predicted"	" 9.5850"	"0.00035132"
1392542_at	"LOC691031"	" -9.5835"	"0.00035218"
1374190_at	"Clybl"	" 9.5772"	"0.00035588"
1373564_at	"Hibch"	" 9.5726"	"0.00035858"
1388105_at	"D123"	" -9.5722"	"0.00035883"
1380323_at	"Slk"	" -9.5709"	"0.00035958"
1384500_at	"RGD1305604_predicted"	" 9.5704"	"0.00035985"
1393615_at	"RGD1561030_predicted"	" 9.5701"	"0.00036007"
1374057_at	"LOC688999"	" 9.5608"	"0.00036562"
1398606_at	"Golph4"	" 9.5578"	"0.00036740"
1373892_at	"Fbxl17_predicted"	" 9.5447"	"0.00037542"
1374418_at	NA	" -9.5427"	"0.00037668"
1386929_at	"Hk1"	" -9.5416"	"0.00037740"
1390965_at	NA	" 9.5412"	"0.00037764"
1370391_at	"Crabp2"	" -9.5410"	"0.00037773"
1367876_at	"Ipo13"	" -9.5379"	"0.00037968"
1388688_at	NA	" 9.5366"	"0.00038048"
1371730_at	"RGD1305466"	" 9.5252"	"0.00038776"
1368311_at	"Mgmt"	" -9.5144"	"0.00039475"
1373099_at	"Pigs"	" -9.5087"	"0.00039850"
1385098_at	NA	" 9.5057"	"0.00040043"
1388703_at	"Esam"	" -9.5057"	"0.00040047"
1392736_at	NA	" 9.5043"	"0.00040139"
1385138_at	NA	" 9.5014"	"0.00040331"
1387117_at	"Zfp265"	" -9.4998"	"0.00040437"
1391539_at	NA	" -9.4918"	"0.00040975"
1372222_at	"RGD1311757_predicted"	" -9.4878"	"0.00041247"
1379832_at	"Polr2d_predicted"	" -9.4836"	"0.00041536"
1394135_at	NA	" 9.4827"	"0.00041602"
1393160_at	NA	" 9.4825"	"0.00041614"
1374834_at	"Sf3b4"	" -9.4765"	"0.00042028"
1387253_at	"Guca2b"	" -9.4744"	"0.00042174"
1381404_at	"Mbd1"	" -9.4706"	"0.00042446"
1372145_at	"Tars"	" -9.4686"	"0.00042581"
1390790_a_at	NA	" -9.4656"	"0.00042799"
1398922_at	NA	" 9.4631"	"0.00042973"
1395887_at	"LOC363060"	" 9.4615"	"0.00043091"
1374427_at	"Syt13"	" -9.4516"	"0.00043806"
1381028_at	"Slc27a4"	" 9.4499"	"0.00043926"
1373944_at	NA	" 9.4448"	"0.00044300"
1372728_at	"Sort1"	" -9.4437"	"0.00044384"
1371336_at	"Hn1"	" -9.4416"	"0.00044538"

1389434_at	RGD1308472_predicted	8.5108	0.00000371
1391062_at	LOC687694	8.5043	0.00000374
1379337_at	RGD1307883	-8.501	0.00000375
1372510_at	Srxn1	-8.5023	0.00000375
1385953_at	Mllt3	8.4995	0.00000376
1382602_at	Ctnnb1	8.4778	0.0000039
1377692_at	RGD1565310_predicted	8.4745	0.00000391
1377597_at	Arid2_predicted	8.4709	0.00000393
1389135_at	Ctps2	8.4717	0.00000393
1374329_at	NA	8.4661	0.00000396
1375350_at	NA	8.4607	0.00000399
1386269_x_at	Sorl1_predicted	8.458	0.000004
1398384_at	Exosc9	-8.4569	0.00000401
1372608_at	LOC313707	-8.4472	0.00000406
1367834_at	Srm	-8.4473	0.00000406
1373767_at	Zfand2a	-8.4471	0.00000406
1373611_at	Il17r_predicted	-8.4424	0.00000409
1371552_at	NA	-8.4402	0.0000041
1373055_at	NA	8.4399	0.0000041
1372220_at	NA	8.437	0.00000411
1377287_at	Mars2_predicted	-8.4314	0.00000415
1385028_at	NA	-8.4272	0.00000417
1371684_at	Ndufs4	-8.4284	0.00000417
1398894_at	Commd3	-8.4246	0.00000418
1390448_at	RGD1308317_predicted	-8.4235	0.00000418
1374279_at	Scye1	-8.4247	0.00000418
1391251_at	NA	8.4225	0.00000419
1388507_at	Itgb4bp	-8.416	0.00000422
1372312_at	Ltv1	-8.4156	0.00000422
1371378_at	Sui1-rs1_predicted	-8.4172	0.00000422
1373929_at	Mrps7	-8.408	0.00000427
1394576_at	NA	8.4071	0.00000427
1376357_at	LOC368062	8.4051	0.00000428
1398777_at	Psmb6	-8.405	0.00000428
1370196_at	Pias3	-8.4022	0.0000043
1370025_at	Pip5k2c	-8.4006	0.0000043
1373263_at	LOC690229	-8.3958	0.00000433
1374082_at	RGD1311578	-8.3913	0.00000436
1371828_at	RGD1310861	-8.3899	0.00000437
1373352_at	LOC685385	-8.3851	0.0000044
1376985_at	NA	-8.3844	0.0000044
1376520_at	NA	8.3849	0.0000044
1386959_a_at	Map2k5	8.3772	0.00000444
1374453_at	RGD1560871_predicted	-8.3771	0.00000444
1378153_at	NA	8.3761	0.00000445
1368184_at	Psmd9	-8.375	0.00000445
1393320_at	RGD1310992	-8.374	0.00000445
1395199_at	Eif3s1_predicted	-8.3712	0.00000446
1384087_at	RGD1561402_predicted	-8.3724	0.00000446
1371565_at	Txndc12	-8.3703	0.00000447

1374010_at	"Lig3"	"	-9.4398"	"0.00044672"
1392531_at	NA	"	-9.4392"	"0.00044718"
1393713_at	"Gmds"	"	9.4374"	"0.00044851"
1396090_at	"RGD1304572_predicted"	"	9.4361"	"0.00044948"
1372647_at	NA	"	-9.4335"	"0.00045145"
1390244_at	"Spats2_predicted"	"	-9.4334"	"0.00045149"
1390208_at	"Htatip2_predicted"	"	-9.4331"	"0.00045170"
1381449_s_at	"Tgfa"	"	9.4311"	"0.00045323"
1377691_at	"Sec22l1"	"	-9.4268"	"0.00045649"
1380345_at	NA	"	9.4233"	"0.00045912"
1388130_at	"Epha1_predicted"	"	-9.4142"	"0.00046612"
1370844_at	"Hnrpf"	"	-9.4045"	"0.00047378"
1385213_at	"RGD1563207_predicted"	"	9.4034"	"0.00047462"
1369313_at	"Fhl2"	"	-9.3997"	"0.00047754"
1371964_at	"Grsf1"	"	9.3962"	"0.00048036"
1387885_at	"Fcgrt"	"	-9.3955"	"0.00048093"
1383382_at	"LOC681740"	"	9.3874"	"0.00048743"
1368162_at	"Cst6"	"	9.3843"	"0.00048995"
1398971_at	"RGD1307929"	"	9.3786"	"0.00049472"
1378943_at	"RGD1566072_predicted"	"	9.3755"	"0.00049725"
1370845_at	"Entpd2"	"	-9.3752"	"0.00049746"
1384164_at	NA	"	9.3729"	"0.00049941"
1368045_at	"Slc31a1"	"	9.3600"	"0.00051033"
1367904_at	"Resp18"	"	9.3573"	"0.00051259"
1397698_at	"Fbxl17_predicted"	"	9.3564"	"0.00051338"
1374180_at	NA	"	-9.3541"	"0.00051532"
1371542_at	"Tuba4a"	"	-9.3517"	"0.00051744"
1391169_at	"RGD1562562_predicted"	"	9.3458"	"0.00052257"
1371676_at	"Lass5_predicted"	"	-9.3450"	"0.00052324"
1372882_at	"RGD1308635_predicted"	"	-9.3448"	"0.00052347"
1392952_at	"LOC619561"	"	9.3386"	"0.00052889"
1372402_at	"Nans_predicted"	"	-9.3307"	"0.00053596"
1391585_at	"LOC690164"	"	-9.3307"	"0.00053598"
1371302_at	"LOC502663"	"	9.3291"	"0.00053742"
1373310_at	NA	"	9.3247"	"0.00054141"
1383965_at	"LOC689116"	"	9.3166"	"0.00054876"
1367473_at	"Tomm22"	"	9.3142"	"0.00055094"
1370019_at	"Sult1a1"	"	9.3114"	"0.00055358"
1373620_at	"RGD1307879_predicted"	"	9.3068"	"0.00055782"
1370153_at	"Gdf15"	"	-9.3044"	"0.00056016"
1371806_at	"Dgcr8_predicted"	"	-9.2888"	"0.00057502"
1373845_at	"LOC688235"	"	-9.2879"	"0.00057587"
1379669_at	NA	"	9.2870"	"0.00057673"
1389115_at	"Evpl_predicted"	"	-9.2730"	"0.00059047"
1370020_at	"Slc25a10"	"	9.2714"	"0.00059208"
1395139_at	"Kif16b_predicted"	"	9.2706"	"0.00059283"
1369928_at	"Acta1"	"	9.2704"	"0.00059305"
1383332_at	NA	"	9.2697"	"0.00059376"
1369258_at	"Fut9"	"	9.2668"	"0.00059667"
1370287_a_at	"Tpm1"	"	-9.2653"	"0.00059814"

1389577_at	Cirh1a	-8.3668	0.00000448
1383328_x_at	Pdcd4	8.3668	0.00000448
1374177_at	Taf13_predicted	-8.367	0.00000448
1371931_at	Gtf2i	8.365	0.00000449
1376687_at	Usp1	-8.3625	0.0000045
1376938_at	Ppp2r2a	-8.3605	0.00000451
1395479_at	Bxdc2	-8.3551	0.00000455
1373103_at	Mta2	-8.3526	0.00000455
1383228_at	NA	8.3543	0.00000455
1390755_at	RGD1563497_predicted	8.3534	0.00000455
1388710_at	Rreb1_predicted	8.3535	0.00000455
1379660_at	NA	-8.3512	0.00000456
1372902_at	Alg12_predicted	-8.3459	0.00000457
1371263_a_at	Camk2d	8.3487	0.00000457
1382233_a_at	NA	-8.3495	0.00000457
1378106_at	Phlda2_predicted	-8.3469	0.00000457
1389659_at	RGD1565540_predicted	-8.3466	0.00000457
1398517_at	Ssu72	-8.3462	0.00000457
1370348_at	Ninj1	-8.3448	0.00000458
1372932_at	Nnp1	-8.3394	0.00000459
1372432_at	Prpf3_predicted	-8.34	0.00000459
1368403_at	Rbl2	8.3397	0.00000459
1371952_at	Rbm18_predicted	-8.3422	0.00000459
1383874_at	RGD1560812_predicted	-8.3409	0.00000459
1371885_at	Ckap1_predicted	-8.3375	0.0000046
1367827_at	Ppp2cb	-8.3386	0.0000046
1389530_at	NA	-8.3319	0.00000464
1372391_at	RGD1308469_predicted	-8.3311	0.00000464
1390710_x_at	Sorl1_predicted	8.3305	0.00000464
1379824_at	Tox_predicted	8.3309	0.00000464
1369962_at	Atic	-8.3281	0.00000465
1370223_at	Arfrp1	-8.3247	0.00000466
1373820_at	RGD1304773	8.3261	0.00000466
1381259_at	Stag1_predicted	8.3261	0.00000466
1371324_at	LOC680891	-8.322	0.00000468
1367883_at	Smn1	-8.3218	0.00000468
1372759_at	Cdk9	-8.3188	0.00000469
1385513_at	NA	8.3201	0.00000469
1368277_at	Ppp3ca	8.3163	0.00000471
1379296_at	NA	-8.3128	0.00000473
1376112_a_at	NTF2	-8.31	0.00000475
1372950_at	Bet1l	-8.3035	0.00000477
1377713_at	Ches1_predicted	8.306	0.00000477
1376935_at	Hmgn2	-8.3062	0.00000477
1392615_at	NA	-8.3059	0.00000477
1373933_at	Rapgef2_predicted	8.3035	0.00000477
1393105_at	RGD1562173_predicted	-8.3056	0.00000477
1387125_at	S100a9	-8.3026	0.00000477
1399044_at	Zfp91	8.3048	0.00000477
1388976_at	RGD1305975_predicted	-8.3017	0.00000478

1380168_at	"Etv4_predicted"	"	-9.2649"	"0.00059863"
1377719_a_at	NA	"	9.2640"	"0.00059947"
1377498_at	"Ampd2"	"	-9.2569"	"0.00060669"
1373199_at	"Gla"	"	-9.2533"	"0.00061044"
1371972_at	"RGD1563141_predicted"	"	-9.2529"	"0.00061085"
1385118_at	"LOC364604"	"	-9.2523"	"0.00061146"
1372902_at	"Alg12_predicted"	"	-9.2502"	"0.00061357"
1385342_at	"Twistnb_predicted"	"	-9.2485"	"0.00061537"
1371663_at	"RGD1305138_predicted"	"	-9.2471"	"0.00061680"
1395275_at	"Pus7_predicted"	"	-9.2439"	"0.00062011"
1384960_at	"Cftr"	"	9.2421"	"0.00062205"
1373501_at	"Nek7_predicted"	"	9.2413"	"0.00062290"
1370288_a_at	"Tpm1"	"	-9.2382"	"0.00062616"
1378516_at	"Rimbp2"	"	9.2376"	"0.00062673"
1397854_at	NA	"	9.2375"	"0.00062691"
1368315_at	"Entpd6"	"	9.2364"	"0.00062799"
1369554_at	"Syngr2"	"	-9.2357"	"0.00062873"
1368490_at	"Cd14"	"	-9.2348"	"0.00062973"
1378243_at	"Tmem17"	"	-9.2332"	"0.00063140"
1382076_at	"Slc37a1"	"	9.2326"	"0.00063206"
1397644_at	"Mtap_predicted"	"	-9.2282"	"0.00063679"
1374790_at	"Gnptg"	"	-9.2255"	"0.00063970"
1375910_at	"Cdc42ep3_predicted"	"	-9.2207"	"0.00064494"
1384926_at	NA	"	-9.2187"	"0.00064713"
1397788_at	"Rimbp2"	"	9.2179"	"0.00064792"
1385191_at	NA	"	9.2126"	"0.00065379"
1371396_at	NA	"	-9.2118"	"0.00065465"
1389010_at	"Lta4h"	"	-9.2112"	"0.00065530"
1383603_at	NA	"	9.2023"	"0.00066523"
1394871_at	"Cdc26"	"	9.2002"	"0.00066760"
1388868_at	"Zfp216_predicted"	"	-9.1990"	"0.00066894"
1390155_at	"Abhd6"	"	9.1989"	"0.00066911"
1372764_at	"LOC689601"	"	-9.1802"	"0.00069065"
1367806_at	"Gls"	"	9.1785"	"0.00069264"
1369888_at	"Gcg"	"	9.1755"	"0.00069615"
1393935_at	"LOC680244"	"	9.1745"	"0.00069731"
1375579_at	"LOC690871"	"	-9.1703"	"0.00070237"
1371811_at	"Bscl2"	"	-9.1660"	"0.00070749"
1375108_at	"ND3"	"	9.1639"	"0.00070995"
1368413_at	"Abp1"	"	9.1611"	"0.00071340"
1379794_at	"Gzmb"	"	9.1582"	"0.00071692"
1372772_at	NA	"	9.1573"	"0.00071793"
1376094_at	"Hint3"	"	9.1467"	"0.00073099"
1394136_at	"Papd1_predicted"	"	-9.1438"	"0.00073463"
1395081_at	"RGD1566426_predicted"	"	9.1420"	"0.00073682"
1368599_at	"Slc9a2"	"	9.1393"	"0.00074022"
1393689_at	"Ndufaf1_predicted"	"	-9.1378"	"0.00074212"
1369736_at	"Emp1"	"	9.1213"	"0.00076324"
1372395_at	"RGD1565757_predicted"	"	9.1115"	"0.00077604"
1374122_at	"Myo5c_predicted"	"	-9.1099"	"0.00077826"

1394058_at	NA	8.2968	0.00000481
1374224_at	Eif2ak4_predicted	8.2932	0.00000483
1383148_at	NA	8.2943	0.00000483
1390407_at	Cldnd1	-8.2907	0.00000484
1383734_at	RGD1561626_predicted	8.291	0.00000484
1371383_at	Drap1_predicted	-8.2833	0.0000049
1374676_at	NA	8.2767	0.00000495
1383743_at	Lrrc16_predicted	8.2746	0.00000496
1373551_at	RGD1309762_predicted	8.275	0.00000496
1398360_at	LOC679532	-8.2731	0.00000497
1388715_at	Gars	-8.2678	0.00000501
1395620_at	RGD1306302	-8.2637	0.00000504
1376038_at	Tex2	8.2618	0.00000505
1383538_at	NA	8.2541	0.0000051
1372479_at	NA	8.255	0.0000051
1370188_at	Sfrs10	-8.2548	0.0000051
1369956_at	Ifngr	-8.2507	0.00000513
1374215_at	Plekhj1	-8.2503	0.00000513
1393009_at	RGD1309326	-8.2493	0.00000513
1377045_at	NA	8.2463	0.00000515
1372661_at	Tbl3	-8.2458	0.00000515
1368852_at	Dnaja1	-8.2444	0.00000516
1372145_at	Tars	-8.2449	0.00000516
1396239_at	RGD1566036_predicted	8.2426	0.00000517
1392922_at	Rap2b	-8.2367	0.00000521
1382818_at	RGD1309752	8.2372	0.00000521
1383125_at	Htf9c	-8.2334	0.00000523
1399130_at	LOC502782	-8.2305	0.00000525
1388165_at	PORF-2	8.2298	0.00000525
1389957_at	Ttc3_predicted	8.2307	0.00000525
1372853_at	Rela	-8.2262	0.00000528
1388582_at	Psme3	-8.2245	0.00000529
1387280_a_at	Slc7a5	-8.2234	0.0000053
1392600_a_at	NA	8.2218	0.00000531
1368552_at	Grpel1	-8.2184	0.00000533
1383253_at	NA	-8.2182	0.00000533
1370979_at	Ddx20	-8.2119	0.00000537
1390769_at	LOC314140	-8.2116	0.00000537
1368262_at	Phlpp	8.2122	0.00000537
1388700_at	NA	8.2088	0.0000054
1373217_at	Ehbp1_predicted	8.207	0.00000541
1380982_at	Bcas1	8.2	0.00000547
1389790_at	NA	-8.1977	0.00000548
1376842_at	NA	8.1985	0.00000548
1368977_a_at	Fxc1	-8.1962	0.00000549
1393248_at	Btn2a2_predicted	-8.1942	0.0000055
1374547_at	LOC502663	-8.1919	0.00000552
1389336_at	Pop5_predicted	-8.1899	0.00000553
1377503_at	Riok2	-8.1881	0.00000554
1383073_at	Usp14	-8.1888	0.00000554

1374312_at	"Uck1_predicted"	" -9.1081"	"0.00078063"
1398793_at	"Cdc5l"	" -9.1071"	"0.00078190"
1367452_at	"Sumo2"	" -9.1024"	"0.00078817"
1388920_at	"Bmp6"	" -9.1010"	"0.00079003"
1389039_at	NA	" 9.0944"	"0.00079902"
1372655_at	NA	" 9.0871"	"0.00080898"
1398916_at	"Aurkaip1"	" -9.0871"	"0.00080899"
1372075_at	"LOC362264"	" -9.0802"	"0.00081867"
1389261_at	"RGD1305508_predicted"	" -9.0757"	"0.00082497"
1393794_at	"RGD1565122_predicted"	" 9.0684"	"0.00083521"
1378021_at	NA	" -9.0631"	"0.00084288"
1389667_at	"LOC683897"	" 9.0623"	"0.00084400"
1368016_at	"Pecr"	" 9.0618"	"0.00084477"
1394570_at	NA	" 9.0597"	"0.00084777"
1369473_at	"Pgm1"	" 9.0567"	"0.00085216"
1386943_at	"Pllp"	" -9.0554"	"0.00085407"
1376051_at	"Cryl1"	" 9.0538"	"0.00085632"
1387288_at	"Neurod1"	" 9.0536"	"0.00085663"
1380102_at	"RGD1560964_predicted"	" -9.0519"	"0.00085909"
1367585_a_at	"Atp1a1"	" 9.0460"	"0.00086790"
1398849_at	"H3f3b"	" -9.0411"	"0.00087510"
1395696_at	"RGD1306613_predicted"	" -9.0396"	"0.00087747"
1376891_at	NA	" 9.0342"	"0.00088553"
1387189_at	"Slc22a3"	" 9.0320"	"0.00088891"
1392082_a_at	"Cd7_predicted"	" 9.0312"	"0.00089006"
1383401_at	"LOC500040"	" -9.0287"	"0.00089387"
1372950_at	"Bet1l"	" -9.0283"	"0.00089454"
1395533_at	"Dnd1"	" -9.0273"	"0.00089610"
1374006_at	"Kat3"	" 9.0166"	"0.00091268"
1375278_at	"Trim2"	" 9.0155"	"0.00091439"
1374415_at	"Polr3e_predicted"	" -9.0148"	"0.00091552"
1379363_at	NA	" 9.0088"	"0.00092493"
1394510_at	NA	" 9.0077"	"0.00092666"
1370908_at	"Hdac2"	" -9.0023"	"0.00093526"
1367847_at	"Nupr1"	" 8.9991"	"0.00094038"
1390492_a_at	"Usp40"	" 8.9898"	"0.00095566"
1374981_at	"RGD1311135_predicted"	" -8.9833"	"0.00096625"
1373083_at	"Ppapdc2"	" -8.9831"	"0.00096669"
1380909_at	"Slc25a24_predicted"	" 8.9728"	"0.00098386"
1387924_at	"Ngef"	" 8.9705"	"0.00098775"
1398364_at	"RGD1359529"	" 8.9690"	"0.00099029"
1377019_at	NA	" -8.9659"	"0.00099571"
1371893_at	"Col4a3bp_predicted"	" -8.9641"	"0.00099869"
1374582_at	"Kctd9_predicted"	" -8.9638"	"0.00099932"
1389923_at	"Btbd9"	" 8.9619"	"0.00100257"
1380028_at	"LOC367311"	" -8.9613"	"0.00100348"
1372770_at	NA	" 8.9609"	"0.00100427"
1368133_at	"Mpdz"	" 8.9597"	"0.00100626"
1378685_at	"LOC689836"	" 8.9582"	"0.00100899"
1375029_at	NA	" -8.9551"	"0.00101440"

1383384_at	LOC682988	8.1822	0.00000559
1373803_a_at	Ghr	8.1737	0.00000567
1381476_at	NA	8.1698	0.0000057
1367687_a_at	Pam	8.1684	0.00000571
1392910_at	Bop1	-8.1631	0.00000575
1393264_at	RGD1564833_predicted	8.164	0.00000575
1378325_at	NA	8.1571	0.0000058
1371617_at	Psmd13_predicted	-8.1585	0.0000058
1386910_a_at	Apex1	-8.1492	0.00000588
1371980_at	Atad3a	-8.1471	0.00000588
1389256_at	NA	8.1486	0.00000588
1393162_at	Slc39a6	-8.1478	0.00000588
1378294_at	Fbxl12	-8.1445	0.00000589
1373822_at	RGD1306356	-8.1441	0.00000589
1389910_at	RGD1307399_predicted	-8.1449	0.00000589
1372946_at	RGD1562747_predicted	-8.1441	0.00000589
1378492_at	Farp1_predicted	8.1398	0.00000593
1373191_at	Rab34	8.1391	0.00000593
1393088_at	Rg9mtd1	-8.1378	0.00000594
1373249_at	NA	-8.1355	0.00000596
1377198_at	NA	8.1345	0.00000596
1398353_at	Sar1a	-8.1337	0.00000596
1389545_at	Tbl1xr1_predicted	8.1347	0.00000596
1371846_at	Phgdhl1	8.13	0.00000599
1375045_at	LOC690751	8.1251	0.00000604
1398708_at	Glrp1_predicted	8.1144	0.00000614
1385517_at	Gtf2i	8.1145	0.00000614
1393154_at	NA	-8.1135	0.00000614
1373851_at	NA	8.114	0.00000614
1369630_at	Adk	8.1117	0.00000616
1382187_at	RGD1311086	-8.1106	0.00000616
1393033_at	Yars2	-8.107	0.00000619
1370932_at	Lrp4	8.1036	0.00000623
1387199_a_at	Arhgef9	8.0988	0.00000627
1367701_at	Ramp2	-8.096	0.00000628
1388646_at	RGD1311784_predicted	8.0958	0.00000628
1371410_at	RGD1564058_predicted	-8.0971	0.00000628
1369979_at	Scap2	8.0978	0.00000628
1376519_at	Brp16	-8.0944	0.00000629
1376715_at	Cbara1	8.0915	0.00000632
1378543_at	Hnrpa2b1_predicted	-8.0888	0.00000634
1373612_at	NA	8.0867	0.00000636
1373284_at	Sav1_predicted	-8.0824	0.0000064
1372149_at	Auh_predicted	8.0796	0.00000642
1368980_at	Plce1	8.0802	0.00000642
1397685_at	NA	8.0787	0.00000643
1367579_a_at	Tuba6	-8.078	0.00000643
1389127_at	Smad3	8.0755	0.00000645
1388397_at	Ebna1bp2	-8.0718	0.00000649
1379327_at	Smarca5_predicted	-8.0689	0.00000652

1374945_at	"RGD1359191"	" -8.9529"	"0.00101824"
1370538_at	"Lama3"	" 8.9406"	"0.00104002"
1375144_at	NA	" -8.9395"	"0.00104196"
1395544_at	NA	" -8.9380"	"0.00104459"
1394705_at	NA	" 8.9358"	"0.00104854"
1367938_at	"Ugdh"	" 8.9307"	"0.00105781"
1373748_at	"Pdzrn3_predicted"	" 8.9273"	"0.00106407"
1373702_at	"L3mbtl2"	" -8.9262"	"0.00106609"
1388180_at	"Phax"	" -8.9220"	"0.00107391"
1383231_at	"Snip1"	" -8.9200"	"0.00107755"
1379521_at	"Alg3"	" -8.9101"	"0.00109620"
1394390_at	NA	" -8.9041"	"0.00110763"
1376085_at	"RGD1563037_predicted"	" -8.9038"	"0.00110811"
1378171_at	"Nrp2"	" 8.9013"	"0.00111294"
1372608_at	"LOC313707"	" -8.9008"	"0.00111398"
1373315_at	"Arnt2"	" 8.8994"	"0.00111664"
1382007_at	"Sh3glb1"	" -8.8975"	"0.00112030"
1381103_a_at	"RGD1562579_predicted"	" -8.8965"	"0.00112221"
1373228_at	"RGD1560755_predicted"	" -8.8948"	"0.00112552"
1382094_at	NA	" 8.8910"	"0.00113300"
1385128_at	NA	" 8.8904"	"0.00113416"
1378257_at	"Trex1"	" -8.8893"	"0.00113621"
1380815_at	"LOC304923"	" 8.8877"	"0.00113945"
1372678_at	"RGD1311283_predicted"	" -8.8870"	"0.00114086"
1372763_at	"RGD1307879_predicted"	" 8.8823"	"0.00115009"
1380472_at	"LOC683587"	" 8.8765"	"0.00116178"
1371786_at	"Trim35"	" 8.8765"	"0.00116180"
1389003_at	"RGD1563286_predicted"	" -8.8764"	"0.00116201"
1383938_at	"RGD1566426_predicted"	" 8.8682"	"0.00117855"
1384262_at	"Ppp1r3b"	" -8.8679"	"0.00117926"
1379271_at	"RGD1564914_predicted"	" -8.8663"	"0.00118237"
1397924_at	NA	" -8.8661"	"0.00118291"
1371552_at	NA	" -8.8659"	"0.00118328"
1384887_at	"Slc5a10_predicted"	" 8.8624"	"0.00119035"
1391109_x_at	NA	" 8.8607"	"0.00119394"
1373986_at	NA	" 8.8602"	"0.00119509"
1385109_at	"Josd3"	" -8.8601"	"0.00119513"
1368209_at	"Pdzk1ip1"	" -8.8578"	"0.00119996"
1391134_at	"Tmco3_predicted"	" -8.8487"	"0.00121904"
1371353_at	"Sqstm1"	" -8.8445"	"0.00122799"
1372498_at	"Ciapin1"	" -8.8426"	"0.00123202"
1374420_at	"RGD1308874"	" -8.8408"	"0.00123587"
1370074_at	"Baiap2"	" -8.8371"	"0.00124394"
1382188_at	"RGD1311086"	" -8.8361"	"0.00124593"
1379293_at	"Gzma"	" 8.8276"	"0.00126450"
1372409_at	"Mad2l1bp"	" -8.8256"	"0.00126884"
1392468_at	"RGD1310950_predicted"	" -8.8256"	"0.00126895"
1372104_at	"Tmbim1"	" 8.8192"	"0.00128319"
1373961_at	"MGC95208"	" -8.8191"	"0.00128344"
1371311_at	"Sdhc"	" 8.8180"	"0.00128591"

1379402_at	Abcc4	8.0666	0.00000654
1380690_at	NA	8.0661	0.00000654
1379815_at	LOC679869	8.0621	0.00000658
1388575_at	NA	8.0577	0.00000661
1374437_at	Nars	-8.0584	0.00000661
1372009_at	Yars	-8.0589	0.00000661
1387087_at	Cebpb	-8.0558	0.00000662
1374890_at	Ube2d2	8.0496	0.00000669
1371363_at	Gpd1	-8.0479	0.00000671
1390118_at	RGD1307679	-8.0449	0.00000673
1378958_at	MGC94720	-8.0442	0.00000674
1393813_at	Ttc3_predicted	8.0379	0.00000681
1387871_at	Cfl1	-8.0357	0.00000682
1390036_at	Slc16a6	-8.0362	0.00000682
1377183_at	Wwox_predicted	8.0297	0.00000689
1384330_at	LOC303067	-8.0242	0.00000695
1399096_at	NA	8.0218	0.00000697
1370311_at	Eif2b1	-8.0167	0.00000702
1399023_at	Ric8a	-8.0169	0.00000702
1389496_at	Akap7	8.0107	0.00000708
1373076_at	NA	8.0117	0.00000708
1387074_at	Rgs2	-8.0091	0.0000071
1387048_at	Ddx39	-8.007	0.00000712
1393186_at	RGD1564454_predicted	-8.0065	0.00000712
1394643_at	NA	-8.0048	0.00000713
1382161_at	Mphosph10_predicted	-8.0041	0.00000714
1372497_at	Nbr1	8.0007	0.00000717
1371730_at	RGD1305466	8.0012	0.00000717
1380431_at	RGD1308772_predicted	7.9996	0.00000717
1375845_at	RGD1562920_predicted	7.9995	0.00000717
1377263_at	Havcr2	-7.9968	0.0000072
1374415_at	Polr3e_predicted	-7.9875	0.00000731
1375404_at	Strbp	7.987	0.00000731
1374569_at	Grwd1	-7.986	0.00000732
1383574_at	Man1a_predicted	7.9843	0.00000733
1367495_at	RGD1560211_predicted	-7.9808	0.00000737
1371645_at	Sdf2_predicted	-7.9731	0.00000746
1375956_at	Mnat1	7.971	0.00000748
1371839_at	Sfrs2	-7.9715	0.00000748
1372099_at	LOC297530	7.9697	0.00000749
1398976_at	Ncor1	7.9599	0.00000761
1370353_at	Timm22	-7.9595	0.00000761
1372179_at	Hpcal1	7.9574	0.00000762
1376194_at	NA	7.957	0.00000762
1389622_at	RGD1565889_predicted	7.9572	0.00000762
1370978_at	Scamp1	7.9589	0.00000762
1373708_at	MGC125034	-7.9559	0.00000763
1387924_at	Ngef_predicted	7.9528	0.00000766
1376279_at	Pop1_predicted	-7.9527	0.00000766
1368049_at	Tcp1	-7.9524	0.00000766

1387505_at	"Gnai1"	"	8.8119"	"0.00129952"
1383232_at	NA	"	-8.8116"	"0.00130020"
1383189_at	NA	"	8.8114"	"0.00130075"
1376945_at	NA	"	8.8091"	"0.00130580"
1396561_x_at	"Piga"	"	-8.8022"	"0.00132159"
1368187_at	"Gpnmb"	"	-8.8008"	"0.00132493"
1386410_at	NA	"	-8.7982"	"0.00133079"
1374694_at	"RGD1559931_predicted"	"	8.7973"	"0.00133293"
1374767_at	"RGD1309592"	"	8.7965"	"0.00133487"
1383372_at	"Ptafr"	"	8.7962"	"0.00133546"
1397223_at	"RGD1565609_predicted"	"	-8.7913"	"0.00134707"
1390456_at	NA	"	8.7900"	"0.00134997"
1377541_at	"LOC500726"	"	-8.7896"	"0.00135087"
1390688_at	"Ddx50"	"	-8.7895"	"0.00135117"
1388669_at	"Sf3a3"	"	-8.7888"	"0.00135278"
1390395_at	NA	"	8.7766"	"0.00138186"
1369982_at	"Ap2a2"	"	8.7732"	"0.00139019"
1392818_at	"Gas5"	"	-8.7684"	"0.00140173"
1390069_at	NA	"	8.7663"	"0.00140694"
1372389_at	"Ier2"	"	-8.7645"	"0.00141133"
1373003_at	"Sart3_predicted"	"	-8.7626"	"0.00141612"
1382835_at	NA	"	8.7594"	"0.00142389"
1371703_at	"Ahnak"	"	-8.7584"	"0.00142655"
1390103_at	"Phf2_predicted"	"	-8.7573"	"0.00142918"
1388448_at	"LOC691031"	"	-8.7520"	"0.00144264"
1367854_at	"Acly"	"	-8.7512"	"0.00144457"
1373515_at	"RGD1307414_predicted"	"	-8.7510"	"0.00144509"
1377263_at	"Havcr2"	"	-8.7495"	"0.00144882"
1371692_at	"Mllt11"	"	-8.7383"	"0.00147744"
1378921_at	NA	"	8.7304"	"0.00149800"
1374447_at	"Usp9x_predicted"	"	8.7291"	"0.00150148"
1389123_at	"Ccl6"	"	-8.7284"	"0.00150327"
1390531_at	"RGD1306056_predicted"	"	-8.7272"	"0.00150658"
1398563_at	NA	"	8.7241"	"0.00151465"
1369160_a_at	"Slc4a7"	"	-8.7239"	"0.00151528"
1372459_at	"Vasp_predicted"	"	-8.7215"	"0.00152167"
1387135_at	"Adam15"	"	-8.7183"	"0.00153001"
1373829_at	"Fgfr2"	"	8.7163"	"0.00153541"
1379497_at	NA	"	8.7139"	"0.00154203"
1382953_at	NA	"	8.7135"	"0.00154302"
1368437_at	"Ca4"	"	8.7128"	"0.00154484"
1389782_at	"RGD1305587_predicted"	"	8.7071"	"0.00156036"
1384124_at	NA	"	-8.7063"	"0.00156247"
1388953_at	"Gnl3"	"	-8.7038"	"0.00156941"
1367624_at	"Atf4"	"	-8.7008"	"0.00157780"
1390867_at	NA	"	-8.7007"	"0.00157797"
1390326_at	"Ang1"	"	-8.6978"	"0.00158617"
1376084_a_at	"Espl1_predicted"	"	8.6969"	"0.00158844"
1398753_at	"Akr1a1"	"	-8.6962"	"0.00159050"
1370319_at	"Ppif"	"	-8.6941"	"0.00159636"

1369543_s_at	Gs3	-7.9457	0.00000774
1374886_at	Bcs1l	-7.9439	0.00000775
1389428_at	F10	-7.9441	0.00000775
1392906_at	Ubl4a_predicted	-7.9437	0.00000775
1371483_at	Nnt	7.9362	0.00000785
1371403_at	Cct3	-7.9343	0.00000786
1389968_at	Eif3s10	-7.9344	0.00000786
1371792_at	NA	7.9319	0.00000789
1395998_at	Nol5a	-7.9283	0.00000793
1387506_at	Foxa3	-7.9257	0.00000796
1374842_at	Cebpz_predicted	-7.9203	0.00000802
1393356_at	NA	7.9207	0.00000802
1372358_at	LOC686590	7.9153	0.00000809
1383005_at	NA	-7.9141	0.0000081
1388920_at	Bmp6	7.9115	0.00000812
1380168_at	Etv4_predicted	-7.9114	0.00000812
1395274_at	Dst_predicted	7.9051	0.00000821
1383332_at	NA	7.9031	0.00000823
1367837_at	Psma4	-7.9019	0.00000823
1376340_a_at	Tssc4	-7.9018	0.00000823
1398375_at	Mta3_predicted	7.9009	0.00000824
1371372_at	LOC367808	-7.8995	0.00000825
1367496_at	Tm9sf2	7.8961	0.0000083
1383512_at	Mllt10	7.895	0.00000831
1390951_at	NA	7.8908	0.00000835
1374332_at	Pdcd11_predicted	-7.8915	0.00000835
1393625_at	LOC313934	7.8862	0.00000842
1374956_at	Pcm1	7.8848	0.00000843
1387367_at	Glg1	7.8818	0.00000847
1394778_at	Nfib	7.879	0.00000849
1374738_at	Sdccag10	7.8801	0.00000849
1384210_at	RSB-11-77	7.8776	0.00000851
1389464_at	Lnx1_predicted	7.8755	0.00000853
1398758_at	Arf4	-7.874	0.00000854
1378134_at	Atp8b1_predicted	7.874	0.00000854
1371115_at	Ptpre	7.8728	0.00000855
1389031_at	Mest	7.8709	0.00000857
1388965_at	Ppp2r5e_predicted	7.8707	0.00000857
1368967_at	Eif2b3	-7.8693	0.00000858
1375672_at	RGD1308513	-7.8692	0.00000858
1398981_at	LOC691278	-7.8676	0.00000859
1372348_at	RGD1563580_predicted	7.8679	0.00000859
1381010_at	Kcnd2	7.8668	0.0000086
1384478_at	Mllt3	7.8658	0.0000086
1372185_at	RGD1306582	-7.866	0.0000086
1377838_at	RGD1562218	-7.8634	0.00000863
1373376_at	NA	7.8621	0.00000864
1370252_at	Avpi1	-7.8584	0.00000869
1371999_at	NA	-7.8556	0.00000872
1383437_at	RGD1562478_predicted	7.8555	0.00000872

1375559_at	"LOC686323"	"	8.6845"	"0.00162348"
1392991_at	"LOC682902"	"	-8.6843"	"0.00162404"
1389291_at	"Chchd3_predicted"	"	8.6841"	"0.00162477"
1371216_s_at	"Fut2"	"	-8.6810"	"0.00163345"
1382848_at	"Foxa1"	"	-8.6797"	"0.00163720"
1382230_at	"Klhdc8a"	"	8.6786"	"0.00164029"
1374472_at	"MGC116266"	"	-8.6737"	"0.00165453"
1377254_a_at	"Cohh1_predicted"	"	8.6701"	"0.00166504"
1381003_at	"Zfpn1a2_predicted"	"	-8.6644"	"0.00168177"
1383702_at	NA	"	-8.6616"	"0.00169005"
1376793_at	"RGD1308377_predicted"	"	8.6593"	"0.00169713"
1378587_at	"LOC690586"	"	8.6577"	"0.00170179"
1374279_at	"Scye1"	"	-8.6561"	"0.00170657"
1391849_at	"Mizf_predicted"	"	-8.6477"	"0.00173196"
1380766_a_at	"RGD1563510_predicted"	"	8.6433"	"0.00174537"
1390379_at	"Lmo4"	"	-8.6432"	"0.00174579"
1383582_at	"Tmem54"	"	8.6411"	"0.00175216"
1373822_at	"RGD1306356"	"	-8.6385"	"0.00176024"
1386930_at	"Psmd4"	"	-8.6369"	"0.00176525"
1368668_at	"Plaa"	"	-8.6188"	"0.00182250"
1370243_a_at	"Ptma"	"	-8.6177"	"0.00182595"
1393361_at	"RGD1310922_predicted"	"	-8.6091"	"0.00185392"
1376328_at	"RGD1310819_predicted"	"	-8.6058"	"0.00186468"
1369984_at	"Cox17"	"	8.6026"	"0.00187521"
1371838_at	"Sfrs2"	"	-8.5990"	"0.00188726"
1398591_at	"Ccrl2_predicted"	"	8.5988"	"0.00188784"
1368395_at	"Gpc3"	"	8.5988"	"0.00188804"
1367736_at	"Rraga"	"	-8.5969"	"0.00189414"
1373626_at	NA	"	8.5942"	"0.00190339"
1390271_at	"Plekha6_predicted"	"	8.5883"	"0.00192324"
1388201_at	"Bmp6"	"	-8.5857"	"0.00193210"
1371659_at	"Rhoc_predicted"	"	-8.5852"	"0.00193378"
1367869_at	"Oxr1"	"	-8.5836"	"0.00193926"
1391889_at	"Ddx52"	"	-8.5829"	"0.00194166"
1383855_at	"Degs2"	"	8.5821"	"0.00194447"
1383097_at	"RGD1311783_predicted"	"	-8.5762"	"0.00196493"
1380582_at	"Csf1"	"	8.5757"	"0.00196663"
1383813_at	"Arl2bp"	"	-8.5751"	"0.00196870"
1388107_at	"Ppp2r2d"	"	-8.5745"	"0.00197081"
1374357_at	"Cdc91l1"	"	8.5736"	"0.00197378"
1377121_at	"Dlg5_predicted"	"	8.5720"	"0.00197943"
1388556_at	"Stx6"	"	-8.5664"	"0.00199909"
1383886_at	NA	"	8.5647"	"0.00200509"
1391608_at	"RGD1565449_predicted"	"	8.5576"	"0.00203059"
1382873_at	"Cttnbp2nl_predicted"	"	-8.5531"	"0.00204698"
1383327_at	"RGD1563912_predicted"	"	-8.5515"	"0.00205265"
1383094_at	NA	"	8.5482"	"0.00206454"
1389531_at	"Zfp330_predicted"	"	-8.5478"	"0.00206630"
1398847_at	"Nudt4"	"	8.5431"	"0.00208325"
1398930_at	"Atp6v0b_predicted"	"	-8.5394"	"0.00209711"

1373272_at	Plekha5	7.853	0.00000875
1389683_at	NA	-7.8514	0.00000876
1389021_at	RGD1306332_predicted	-7.8514	0.00000876
1373209_at	RGD1305572	-7.8496	0.00000878
1382269_at	Cnnm2	7.846	0.00000882
1383188_at	NA	7.8465	0.00000882
1381058_at	NA	7.8391	0.00000892
1373664_at	Pigc	-7.8397	0.00000892
1369635_at	Sord	7.8386	0.00000892
1375565_at	Timm22	-7.8364	0.00000895
1377802_at	RGD1566399_predicted	7.8353	0.00000896
1372593_at	Zfp110	-7.8335	0.00000898
1388802_at	Bex1	-7.8312	0.000009
1373989_at	Rassf1	-7.832	0.000009
1373241_at	Mrpl49	-7.8285	0.00000904
1398877_at	Stip1	-7.828	0.00000904
1388616_at	MGC72955	-7.8254	0.00000907
1384032_at	NA	-7.8252	0.00000907
1376101_at	Lrp6_predicted	7.8236	0.00000909
1372837_at	NA	7.8221	0.0000091
1367898_at	Bnip3l	7.8208	0.00000911
1388321_at	Imp3_predicted	-7.8205	0.00000911
1368508_at	Psma3l	-7.8192	0.00000912
1372028_at	RGD1305727_predicted	-7.8198	0.00000912
1372643_at	RGD1563977_predicted	7.8171	0.00000915
1392209_at	Gcnt3	-7.8135	0.0000092
1397634_at	RGD1306359_predicted	7.8108	0.00000924
1382183_at	NA	7.8091	0.00000925
1389601_at	Nfib	7.809	0.00000925
1398783_at	Gps1	-7.8065	0.00000928
1373155_at	Mrpl46	-7.8064	0.00000928
1388554_at	Bzw1	-7.8039	0.00000931
1370614_s_at	Stk39	7.8007	0.00000936
1374444_at	Plxnb1_predicted	-7.798	0.0000094
1381555_at	Bclaf1	-7.7943	0.00000945
1375337_at	Adam9_predicted	7.7928	0.00000947
1389159_at	NA	7.7924	0.00000947
1397719_at	NA	-7.7903	0.0000095
1375029_at	NA	-7.7886	0.0000095
1389686_at	Prkx	-7.7892	0.0000095
1373846_at	RGD1561589_predicted	-7.7898	0.0000095
1382993_at	Bbc3	-7.7877	0.00000951
1395362_at	NA	7.7864	0.00000952
1389522_at	Plekha6_predicted	7.7868	0.00000952
1393228_at	Gloxd1	-7.784	0.00000954
1393096_at	Stxbp5	7.7846	0.00000954
1388679_at	Tbc1d14	7.782	0.00000957
1376407_a_at	Lsm7_predicted	-7.7782	0.00000963
1399002_at	Mrps17_predicted	-7.7731	0.00000971
1376690_at	Surb7_predicted	-7.7727	0.00000971

1374522_at	"LOC683007"	"	8.5374"	"0.00210468"
1382219_at	"RGD1307509"	"	-8.5367"	"0.00210716"
1386754_at	NA	"	8.5365"	"0.00210796"
1367578_at	"Prdx2"	"	-8.5331"	"0.00212084"
1368661_at	"Slc13a2"	"	8.5289"	"0.00213644"
1384907_at	"LOC306096"	"	8.5287"	"0.00213736"
1370223_at	"Arfrp1"	"	-8.5281"	"0.00213971"
1398317_at	"Bpnt1"	"	8.5235"	"0.00215702"
1367827_at	"Ppp2cb"	"	-8.5227"	"0.00216027"
1390566_a_at	"Ckmt1"	"	-8.5223"	"0.00216175"
1367811_at	"Phgdh"	"	-8.5194"	"0.00217303"
1375950_a_at	NA	"	-8.5177"	"0.00217955"
1377644_at	"RGD1308706_predicted"	"	8.5162"	"0.00218545"
1370286_at	"Slc38a2"	"	8.5148"	"0.00219062"
1383457_at	"RGD1566090_predicted"	"	-8.5120"	"0.00220183"
1389576_at	"Snrpb2_predicted"	"	-8.5044"	"0.00223157"
1372506_at	"Psme3"	"	-8.5028"	"0.00223780"
1378543_at	"Hnrpa2b1_predicted"	"	-8.5010"	"0.00224512"
1384017_at	"Lrrk1_predicted"	"	8.4972"	"0.00226058"
1374432_at	"Alcam"	"	8.4965"	"0.00226306"
1387726_at	"Cdx2"	"	8.4954"	"0.00226765"
1382411_at	NA	"	-8.4935"	"0.00227513"
1374648_at	NA	"	-8.4930"	"0.00227735"
1378027_at	"Pvrl3_predicted"	"	8.4876"	"0.00229952"
1374949_at	"LOC499871"	"	-8.4869"	"0.00230205"
1389032_at	"Mcoln1_predicted"	"	8.4826"	"0.00231995"
1389161_at	NA	"	8.4758"	"0.00234821"
1388960_at	"Pyp"	"	-8.4738"	"0.00235668"
1388051_at	"Slc26a3"	"	8.4704"	"0.00237103"
1379665_at	"Ppwd1_predicted"	"	-8.4622"	"0.00240583"
1388444_at	"Ubxd2"	"	-8.4590"	"0.00241940"
1398308_at	"Rpa3_predicted"	"	8.4588"	"0.00242026"
1375845_at	"RGD1562920_predicted"	"	8.4554"	"0.00243511"
1373405_at	NA	"	-8.4514"	"0.00245269"
1370050_at	"Atp2b1"	"	8.4508"	"0.00245507"
1386671_at	"Ift74"	"	8.4499"	"0.00245931"
1367833_at	"Psmc5"	"	-8.4497"	"0.00245987"
1399117_at	"RGD1311745"	"	-8.4443"	"0.00248370"
1374580_at	"Senp6_predicted"	"	-8.4431"	"0.00248937"
1370070_at	"Synj1"	"	8.4404"	"0.00250140"
1383153_at	NA	"	8.4393"	"0.00250608"
1389649_at	NA	"	8.4385"	"0.00250959"
1385529_at	"RGD1311662_predicted"	"	8.4382"	"0.00251094"
1397917_at	NA	"	8.4334"	"0.00253254"
1395694_at	"Fbxo9"	"	-8.4323"	"0.00253782"
1370062_at	"Hig1"	"	8.4313"	"0.00254232"
1373393_at	"LOC299907"	"	8.4312"	"0.00254276"
1394169_at	NA	"	-8.4283"	"0.00255568"
1372083_at	"Polr2b_predicted"	"	-8.4258"	"0.00256716"
1376868_at	"Cobll1_predicted"	"	8.4206"	"0.00259145"

1371597_at	Rnf187_predicted	-7.7715	0.00000972
1385138_at	NA	7.7687	0.00000975
1398430_at	NA	7.7692	0.00000975
1395079_at	NA	-7.767	0.00000977
1375856_at	NA	7.7664	0.00000977
1367956_at	Ncdn	-7.7663	0.00000977
1372389_at	Ier2	-7.7606	0.00000986
1384029_at	Xpa_predicted	7.7606	0.00000986
1389019_at	NA	7.7598	0.00000987
1372463_at	Fcho2_predicted	7.7567	0.00000991
1388286_a_at	Cdc34_predicted	-7.7527	0.00000998
1387864_at	Kidins220	7.7501	0.00001
1385261_s_at	NA	7.7498	0.00001
1374153_at	Pdcl3	-7.7495	0.00001
1383415_at	Ppp3ca	7.7498	0.00001
1389011_at	RGD1305156	7.7479	0.00001001
1385427_at	RGD1560397_predicted	7.7481	0.00001001
1379810_at	Scye1	-7.7469	0.00001002
1377180_at	NA	7.7446	0.00001006
1376761_at	Hdac4	7.739	0.00001015
1371884_at	Ttc3_predicted	7.735	0.00001022
1374820_at	NA	-7.7337	0.00001023
1398895_at	Golga7	-7.7296	0.00001028
1368371_at	Kcnq1	7.7308	0.00001028
1373589_at	Mtmr3	7.7305	0.00001028
1387117_at	Zfp265	-7.7297	0.00001028
1399012_at	RGD1310313_predicted	7.7282	0.00001029
1374519_at	Ggcx	7.7211	0.00001041
1370066_at	Keap1	-7.7219	0.00001041
1373590_at	Stom	-7.7205	0.00001042
1398908_at	Stoml2	-7.7159	0.00001049
1378253_at	Gnpat	-7.7126	0.00001052
1372869_at	Gtpbp4	-7.714	0.00001052
1371351_at	MGC112727	-7.7133	0.00001052
1382050_at	NA	7.7123	0.00001052
1379406_at	RGD1308396_predicted	-7.7138	0.00001052
1375579_at	LOC690871	-7.71	0.00001056
1370368_at	Cabin1	7.7058	0.00001061
1383543_at	Golt1b_predicted	-7.7061	0.00001061
1379305_at	NA	7.707	0.00001061
1371838_at	Sfrs2	-7.7022	0.00001067
1384940_at	RGD1305314	-7.6991	0.00001073
1385133_at	LOC691729	-7.6979	0.00001074
1377708_at	LOC499339	-7.6957	0.00001076
1389050_at	RGD1560049_predicted	-7.6959	0.00001076
1372417_at	Sertad1	-7.6962	0.00001076
1393142_at	LOC367153	7.694	0.00001078
1368268_at	Tdg	-7.6937	0.00001078
1376786_a_at	NA	7.6882	0.00001088
1379407_at	Imp4	-7.6856	0.00001091

1383085_at	"Sh3bgrl_predicted"	"	8.4160"	"0.00261270"
1370583_s_at	"Abcb1b"	"	8.4142"	"0.00262112"
1390119_at	"Sfrp2"	"	-8.4142"	"0.00262121"
1379488_at	"Trp53rk_predicted"	"	-8.4069"	"0.00265550"
1369224_at	"Cdh17"	"	8.4053"	"0.00266326"
1379136_at	NA	"	8.4043"	"0.00266828"
1382143_at	"Farp1_predicted"	"	8.4022"	"0.00267816"
1377112_at	"Cda_predicted"	"	8.4014"	"0.00268175"
1377096_at	"Mtfmt"	"	8.4000"	"0.00268866"
1391153_at	NA	"	-8.3989"	"0.00269382"
1389013_at	"RGD1308469_predicted"	"	-8.3969"	"0.00270359"
1393317_at	"RGD1560852_predicted"	"	8.3947"	"0.00271445"
1398952_at	"RGD1564093_predicted"	"	-8.3933"	"0.00272104"
1370009_at	"Apoc3"	"	8.3928"	"0.00272352"
1382341_at	"RGD1561878_predicted"	"	8.3915"	"0.00273006"
1389072_at	"Mtmr4_predicted"	"	8.3897"	"0.00273862"
1373732_at	"Acp6"	"	8.3896"	"0.00273907"
1389102_at	"RGD1566072_predicted"	"	8.3874"	"0.00275028"
1374521_at	NA	"	-8.3843"	"0.00276542"
1377842_at	"Twistnb_predicted"	"	-8.3810"	"0.00278165"
1388321_at	"Imp3_predicted"	"	-8.3807"	"0.00278330"
1377656_at	"Rbm13"	"	-8.3798"	"0.00278787"
1373105_at	"Tmed1"	"	-8.3793"	"0.00279008"
1374976_a_at	"Soat1"	"	8.3783"	"0.00279548"
1370043_at	"Alcam"	"	8.3776"	"0.00279864"
1389204_at	"RGD1564456_predicted"	"	-8.3709"	"0.00283247"
1376287_at	"Capn13"	"	-8.3697"	"0.00283873"
1372092_at	"Trak2"	"	8.3692"	"0.00284145"
1374987_at	NA	"	8.3612"	"0.00288237"
1369336_at	"Hr"	"	-8.3603"	"0.00288734"
1372337_at	"Keap1"	"	-8.3593"	"0.00289241"
1390410_at	NA	"	-8.3583"	"0.00289769"
1388825_at	NA	"	8.3573"	"0.00290257"
1398898_at	"Ensa"	"	-8.3531"	"0.00292479"
1399090_at	"Dync1li1"	"	-8.3526"	"0.00292729"
1378568_a_at	"RGD1306228_predicted"	"	-8.3524"	"0.00292849"
1390699_at	"RGD1311595"	"	8.3501"	"0.00294029"
1379255_at	"Atp6ap2"	"	-8.3484"	"0.00294974"
1387790_at	"Paics"	"	-8.3420"	"0.00298370"
1388887_at	NA	"	8.3416"	"0.00298600"
1375362_at	"RGD1563001_predicted"	"	8.3397"	"0.00299619"
1376620_at	NA	"	8.3394"	"0.00299742"
1387264_at	"Kcnk6"	"	-8.3390"	"0.00299995"
1375423_at	"LOC689959"	"	-8.3371"	"0.00301029"
1395063_at	"Mrpl14_predicted"	"	-8.3312"	"0.00304202"
1388875_at	"Cxxc1"	"	-8.3301"	"0.00304840"
1389507_at	"Nedd4l"	"	8.3270"	"0.00306545"
1368106_at	"Plk2"	"	-8.3212"	"0.00309747"
1367636_at	"Igf2r"	"	8.3174"	"0.00311882"
1388554_at	"Bzw1"	"	-8.3170"	"0.00312084"

1373369_at	NA	7.6859	0.00001091
1380102_at	RGD1560964_predicted	-7.6849	0.00001092
1392947_at	NA	7.6827	0.00001095
1375418_at	Trrap_predicted	7.6819	0.00001096
1383159_at	NA	7.6806	0.00001098
1388546_at	Ablim1_predicted	7.6791	0.000011
1384340_a_at	Ard1_predicted	-7.677	0.00001102
1377156_at	LOC679869	7.6768	0.00001102
1377959_at	RGD1561287_predicted	-7.6772	0.00001102
1373813_at	Dnajc10	-7.6731	0.00001108
1392454_at	RGD1308723_predicted	-7.6731	0.00001108
1383127_at	Adam10	7.6678	0.00001118
1370144_at	Gtpbp4	-7.6661	0.0000112
1372772_at	NA	7.6632	0.00001125
1379526_at	Mbp	7.6595	0.00001132
1372589_at	LOC683512	7.6581	0.00001134
1373384_at	LOC691318	7.6563	0.00001137
1389193_at	Sorcs2_predicted	7.654	0.00001141
1393177_at	LOC502020	7.6529	0.00001142
1378098_at	RGD1309748_predicted	-7.6521	0.00001143
1371936_at	Eif4a1	-7.646	0.00001155
1370295_at	Nme1	-7.6443	0.00001157
1397512_at	RGD1560511_predicted	7.6429	0.0000116
1371988_at	Man1a_predicted	7.6396	0.00001166
1393119_at	NA	-7.6348	0.00001175
1375904_at	Tmed5	-7.6329	0.00001178
1392989_at	Mib1_predicted	7.6311	0.00001181
1389556_at	Kifap3_predicted	7.6266	0.00001189
1384185_at	RGD1307704_predicted	-7.6265	0.00001189
1373986_at	NA	7.6254	0.00001191
1374729_at	LOC308846	7.6241	0.00001192
1387801_at	Ppp6c	-7.6241	0.00001192
1376753_at	Fpgt	-7.6196	0.00001199
1393363_at	LOC305913	7.6201	0.00001199
1374721_at	NA	-7.619	0.00001199
1389664_at	Rbm15b_predicted	-7.6205	0.00001199
1383044_at	NA	7.6157	0.00001205
1391320_at	Hspbap1	-7.6147	0.00001207
1376489_at	Acp1	7.6121	0.00001211
1378048_at	LOC679869	7.612	0.00001211
1367671_at	Pcna	-7.6099	0.00001215
1382313_at	NA	7.6089	0.00001216
1392918_at	Cct8_predicted	-7.608	0.00001217
1389106_at	Fbxw9	-7.6042	0.00001225
1372728_at	Sort1	7.602	0.00001229
1379282_at	Lrrfip2	7.6012	0.0000123
1374288_at	Ftsj3	-7.5988	0.00001231
1376812_at	Ing5_predicted	-7.5996	0.00001231
1384002_at	LOC361929	7.5999	0.00001231
1373632_at	Taf9	-7.5988	0.00001231

1388330_at	"Vkorc1"	"	-8.3165"	"0.00312409"
1371985_a_at	"Bat5"	"	8.3126"	"0.00314573"
1368806_at	"RGD1561503_predicted"	"	8.3106"	"0.00315702"
1393972_at	"Mtf2"	"	-8.3074"	"0.00317573"
1389371_at	NA	"	8.2986"	"0.00322612"
1395136_at	NA	"	8.2984"	"0.00322748"
1371390_at	"Tubb2c"	"	-8.2928"	"0.00326053"
1382521_at	"Gls"	"	8.2887"	"0.00328440"
1384097_at	"RGD1311662_predicted"	"	8.2826"	"0.00332071"
1372941_at	"Pdrg1"	"	-8.2826"	"0.00332076"
1372593_at	"Zfp110"	"	-8.2806"	"0.00333271"
1390388_at	"Fech_predicted"	"	-8.2791"	"0.00334167"
1390615_at	"Kpna1"	"	-8.2758"	"0.00336218"
1376569_at	"Klf2_predicted"	"	-8.2744"	"0.00337023"
1371448_at	NA	"	8.2723"	"0.00338341"
1378575_at	"Asl"	"	8.2679"	"0.00341026"
1372676_at	"Fahd1"	"	8.2670"	"0.00341598"
1383625_a_at	"RGD1562173_predicted"	"	-8.2646"	"0.00343099"
1378161_at	NA	"	8.2630"	"0.00344043"
1368921_a_at	"Cd44"	"	8.2619"	"0.00344779"
1367589_at	"Aco2"	"	8.2580"	"0.00347203"
1390931_at	NA	"	-8.2574"	"0.00347578"
1389323_at	"Wdr61"	"	-8.2562"	"0.00348306"
1380389_at	"Ptger3"	"	-8.2558"	"0.00348575"
1374291_at	"Dnajc17_predicted"	"	-8.2550"	"0.00349105"
1372185_at	"RGD1306582"	"	-8.2533"	"0.00350155"
1384027_a_at	NA	"	8.2508"	"0.00351747"
1394767_at	"LOC684205"	"	8.2474"	"0.00353900"
1389319_at	"LOC287177"	"	8.2462"	"0.00354698"
1373293_at	"Lsg1"	"	-8.2446"	"0.00355738"
1375989_a_at	"Nfkb2"	"	-8.2438"	"0.00356244"
1380255_at	NA	"	-8.2435"	"0.00356416"
1373619_at	"Ankrd10"	"	-8.2420"	"0.00357383"
1395560_at	NA	"	-8.2402"	"0.00358561"
1372634_at	"Adprhl2_predicted"	"	-8.2378"	"0.00360107"
1371640_at	"LOC500855"	"	-8.2357"	"0.00361491"
1367972_at	"Cand1"	"	-8.2356"	"0.00361581"
1371454_at	"Tmem93_predicted"	"	-8.2315"	"0.00364262"
1388777_at	"Ssr3"	"	-8.2263"	"0.00367699"
1372252_at	"Trappc1"	"	8.2261"	"0.00367869"
1383055_at	"Gnaq"	"	8.2233"	"0.00369738"
1387028_a_at	"Id1"	"	-8.2212"	"0.00371108"
1372366_at	"Htatip"	"	-8.2206"	"0.00371512"
1375267_at	"Ppic"	"	8.2202"	"0.00371781"
1393062_at	NA	"	-8.2181"	"0.00373224"
1388603_a_at	"Hbld2"	"	-8.2176"	"0.00373557"
1368883_at	"Nov"	"	-8.2109"	"0.00378164"
1388413_at	"Rrbp1_predicted"	"	8.2065"	"0.00381202"
1393088_at	"Rg9mtd1"	"	-8.2046"	"0.00382466"
1377637_at	NA	"	-8.2046"	"0.00382475"

1379074_at	NA	-7.5956	0.00001237
1397620_at	Sh3md2	-7.5953	0.00001237
1385767_at	LOC304000	7.5902	0.00001248
1368817_at	Psme4	7.5889	0.0000125
1373743_at	RGD1305062	-7.5884	0.0000125
1382223_at	Zmym4_predicted	7.584	0.00001259
1389442_at	NA	7.5821	0.00001263
1382811_at	NA	-7.5804	0.00001266
1379652_at	LOC682398	-7.5773	0.00001272
1373160_at	LOC680466	-7.5747	0.00001277
1392043_at	Insr	7.5733	0.00001278
1384439_at	NA	-7.5741	0.00001278
1374252_at	RGD1563798_predicted	7.5703	0.00001283
1377458_at	Sorl1_predicted	7.5707	0.00001283
1373652_at	RGD1564833_predicted	7.5691	0.00001285
1376113_at	Zswim1_predicted	-7.5627	0.000013
1389287_at	NA	7.5611	0.00001302
1373791_at	RGD1359127	-7.559	0.00001307
1391014_at	Zmynd19	-7.5552	0.00001315
1390514_at	MGC94223	-7.5516	0.00001322
1372026_at	RGD1359310	-7.5514	0.00001322
1371596_at	Rnps1	-7.5443	0.00001338
1381472_at	Slc25a32_predicted	-7.5426	0.00001341
1373671_at	LOC688786	-7.5389	0.00001348
1373556_at	RGD1561792_predicted	-7.5393	0.00001348
1382536_at	Thrap2_predicted	7.5366	0.00001353
1376664_at	LOC246187	-7.5357	0.00001354
1376994_at	NA	7.536	0.00001354
1382396_at	RGD1306614	-7.5347	0.00001355
1369680_at	Slc2a13	7.5336	0.00001357
1392938_s_at	RGD1306959_predicted	-7.5322	0.00001359
1377825_at	NA	7.5289	0.00001366
1389587_at	Umps	-7.5266	0.00001371
1377121_at	Dlg5_predicted	7.5243	0.00001375
1374713_at	NA	-7.5238	0.00001375
1372795_at	Txnl4b	-7.5247	0.00001375
1376351_at	NA	-7.5222	0.00001377
1381467_at	Neo1	7.5223	0.00001377
1382441_at	Arid1b	7.5177	0.00001388
1384472_at	LOC362683	7.5155	0.00001392
1383212_at	NA	7.5143	0.00001394
1378860_at	NA	7.5104	0.00001402
1384150_at	NA	7.5109	0.00001402
1395010_at	NA	7.5095	0.00001404
1372115_at	LOC363188	7.5055	0.00001413
1378434_at	Setbp1_predicted	7.5036	0.00001416
1373607_at	St3gal3	7.5041	0.00001416
1373293_at	Lsg1	-7.5018	0.00001418
1398869_at	Psmc4	-7.502	0.00001418
1390875_a_at	RGD1359593	7.5011	0.00001418

1379027_at	"RGD1308329_predicted"	" 8.1977"	"0.00387343"
1371988_at	"Man1a_predicted"	" 8.1956"	"0.00388813"
1389786_at	"LOC500419"	" 8.1930"	"0.00390645"
1386002_at	NA	" 8.1924"	"0.00391039"
1392875_at	"Fbxl4_predicted"	" 8.1917"	"0.00391560"
1387692_a_at	"Sstr1"	" -8.1909"	"0.00392147"
1368379_at	"Scarb2"	" 8.1903"	"0.00392549"
1393558_at	"Itga6"	" -8.1903"	"0.00392598"
1376164_at	"Sf4"	" -8.1889"	"0.00393530"
1389048_at	"Bmp1"	" 8.1887"	"0.00393706"
1380067_at	"Myo5c_predicted"	" -8.1880"	"0.00394186"
1367877_at	"Slc11a2"	" -8.1865"	"0.00395258"
1389597_at	"Pgbd5_predicted"	" 8.1819"	"0.00398642"
1367929_at	"Cd59"	" -8.1765"	"0.00402531"
1367762_at	"Sst"	" 8.1715"	"0.00406221"
1382276_at	"Coro1c_predicted"	" -8.1701"	"0.00407274"
1376071_at	NA	" 8.1689"	"0.00408128"
1372774_at	"Coq6"	" 8.1655"	"0.00410704"
1372837_at	NA	" 8.1641"	"0.00411722"
1374011_at	"Ercc3"	" -8.1639"	"0.00411855"
1379651_at	"Foxp1"	" -8.1628"	"0.00412736"
1389968_at	"Eif3s10"	" -8.1584"	"0.00416051"
1371839_at	"Sfrs2"	" -8.1584"	"0.00416059"
1374764_at	"RGD1305605_predicted"	" -8.1570"	"0.00417109"
1378705_at	"RGD1310433_predicted"	" 8.1567"	"0.00417294"
1372599_at	"Mgst2_predicted"	" 8.1566"	"0.00417427"
1388705_at	"RGD1565037_predicted"	" 8.1557"	"0.00418126"
1389519_at	"Psmd8"	" -8.1544"	"0.00419096"
1367632_at	"Glul"	" 8.1513"	"0.00421420"
1373729_at	NA	" 8.1497"	"0.00422720"
1370893_at	"Acaca"	" 8.1479"	"0.00424054"
1398430_at	NA	" 8.1474"	"0.00424486"
1388447_at	"LOC683626"	" 8.1472"	"0.00424587"
1393866_at	"LOC500592"	" -8.1464"	"0.00425237"
1382144_at	"Mrpl47"	" -8.1463"	"0.00425293"
1381705_at	NA	" 8.1457"	"0.00425774"
1388446_at	NA	" -8.1455"	"0.00425964"
1372221_at	NA	" 8.1440"	"0.00427091"
1398908_at	"Stoml2"	" -8.1397"	"0.00430443"
1386379_at	NA	" 8.1369"	"0.00432700"
1370190_at	"H3f3b"	" -8.1325"	"0.00436141"
1374620_at	"Ceacam1"	" -8.1323"	"0.00436359"
1379057_at	"LOC683460"	" 8.1281"	"0.00439699"
1368283_at	"Ehhadh"	" 8.1273"	"0.00440344"
1387766_a_at	"Rbp2"	" 8.1260"	"0.00441410"
1368236_at	"Mep1a"	" 8.1259"	"0.00441450"
1382165_at	"Smndc1"	" -8.1226"	"0.00444115"
1387916_at	"Cyp4f6"	" 8.1205"	"0.00445860"
1380100_at	"RGD1561817_predicted"	" 8.1180"	"0.00447841"
1373160_at	"LOC680466"	" -8.1160"	"0.00449542"

1390813_at	RGD1560397_predicted	7.5014	0.00001418
1373499_at	Gas5	-7.4989	0.00001421
1388311_at	Mrfap1	-7.499	0.00001421
1390527_at	RGD1562114_predicted	-7.4997	0.00001421
1369629_at	Adk	7.4983	0.00001422
1383224_at	NA	-7.4968	0.00001424
1374030_at	LOC684112	7.4953	0.00001427
1373319_at	Ddx1	-7.494	0.00001429
1375441_at	Sars1	-7.4941	0.00001429
1374063_at	Sfrs3_predicted	-7.4879	0.00001444
1392888_at	Gpc4	7.486	0.00001447
1383970_at	NA	7.4859	0.00001447
1372460_at	Pkn3	-7.4817	0.00001457
1389559_at	NA	7.4781	0.00001466
1388555_at	Txnl5_predicted	-7.4761	0.0000147
1383685_at	Heatr1_predicted	-7.4755	0.00001471
1371957_at	Imp4	-7.475	0.00001471
1372078_at	Strap	-7.4722	0.00001478
1371907_at	Arl6ip4	-7.465	0.00001495
1368971_a_at	Synj2	7.4655	0.00001495
1383602_at	Pum1_predicted	7.4628	0.00001499
1373821_at	Slc30a5_predicted	-7.4632	0.00001499
1382060_at	NA	7.4612	0.00001502
1376659_at	Nubpl_predicted	7.4609	0.00001502
1388516_at	LOC499235	-7.4591	0.00001505
1374657_at	LOC687561	-7.4591	0.00001505
1388428_at	Hars2_predicted	7.4579	0.00001507
1389778_a_at	Tceb3	-7.4565	0.0000151
1383921_at	NA	-7.455	0.00001513
1371971_at	RGD1560212_predicted	-7.4518	0.00001521
1378921_at	NA	7.4493	0.00001527
1388351_at	RGD1305831_predicted	-7.4436	0.00001542
1371551_at	Traf4_predicted	-7.4429	0.00001543
1373240_at	Dhrs3	7.4341	0.00001563
1371515_at	LOC691193	-7.4353	0.00001563
1373455_at	Paf1	-7.4346	0.00001563
1376824_at	RGD1311815	7.4352	0.00001563
1373195_at	RGD1566093_predicted	-7.4346	0.00001563
1377827_at	Srfbp1	-7.4303	0.00001573
1398792_at	Psmc1	-7.4261	0.00001584
1372062_at	RGD1563395_predicted	-7.4237	0.0000159
1397892_at	Eif1a	-7.4227	0.00001592
1373602_at	Lars2_predicted	7.4195	0.00001599
1379858_at	Mettl2_predicted	-7.4199	0.00001599
1376620_at	NA	7.4189	0.000016
1388416_at	Lrp1	7.4171	0.00001604
1392405_at	Tcfdp2_predicted	7.4126	0.00001616
1376206_at	NA	-7.4111	0.0000162
1381623_at	RGD1561347_predicted	-7.4077	0.00001629
1369906_s_at	Mcfd2	-7.4028	0.00001642

1375997_at	NA	"	8.1158"	"0.00449690"
1380895_at	"Aplp2"	"	-8.1155"	"0.00449961"
1370135_at	"Cav2"	"	-8.1132"	"0.00451858"
1369956_at	"Ifngr1"	"	8.1127"	"0.00452249"
1393105_at	"RGD1562173_predicted"	"	-8.1126"	"0.00452293"
1387306_a_at	"Egr2"	"	-8.1124"	"0.00452469"
1371498_at	"MGC125271"	"	-8.1096"	"0.00454790"
1371961_at	"Pld3"	"	-8.1044"	"0.00459119"
1373379_at	"Irak1_predicted"	"	-8.1022"	"0.00460970"
1374197_at	NA	"	8.1012"	"0.00461891"
1389459_at	"RGD1306613_predicted"	"	-8.0992"	"0.00463546"
1374070_at	"Gpx2"	"	-8.0921"	"0.00469616"
1380097_at	"Laptm4a"	"	-8.0914"	"0.00470206"
1376659_at	"Nubpl_predicted"	"	8.0889"	"0.00472397"
1371971_at	"RGD1560212_predicted"	"	-8.0865"	"0.00474489"
1376692_at	"Hipk2_predicted"	"	8.0843"	"0.00476400"
1372454_at	"RGD1311899"	"	-8.0820"	"0.00478408"
1370979_at	"Ddx20"	"	-8.0818"	"0.00478591"
1398900_at	"Dctn3_predicted"	"	-8.0761"	"0.00483550"
1375915_at	"Irak1bp1_predicted"	"	-8.0760"	"0.00483705"
1373673_at	NA	"	8.0754"	"0.00484203"
1398391_at	"Gnl1"	"	-8.0740"	"0.00485457"
1395587_at	"RGD1561254_predicted"	"	-8.0718"	"0.00487425"
1381181_at	NA	"	8.0715"	"0.00487671"
1383907_at	NA	"	8.0697"	"0.00489303"
1371456_at	"Abcf3"	"	-8.0610"	"0.00497183"
1384475_at	"Plekha5"	"	8.0581"	"0.00499780"
1368027_at	"Tbxas1"	"	8.0565"	"0.00501272"
1398899_at	"Polr2c"	"	-8.0556"	"0.00502134"
1384282_at	"Zipro1"	"	-8.0526"	"0.00504910"
1374177_at	"Taf13_predicted"	"	-8.0517"	"0.00505772"
1379652_at	"Ahctf1_predicted"	"	-8.0488"	"0.00508452"
1392083_at	"LOC299907"	"	8.0466"	"0.00510530"
1396000_at	NA	"	8.0394"	"0.00517247"
1374852_at	"RGD1559909_predicted"	"	-8.0316"	"0.00524732"
1395065_at	"Wdr41_predicted"	"	-8.0316"	"0.00524756"
1398876_at	"Abcf1"	"	-8.0295"	"0.00526815"
1371868_at	"Bcas2_predicted"	"	-8.0294"	"0.00526924"
1374571_at	"Pigx"	"	-8.0283"	"0.00527920"
1376937_at	"RGD1565927_predicted"	"	8.0262"	"0.00529963"
1390502_at	"RGD1309104_predicted"	"	-8.0241"	"0.00532043"
1375181_at	"LOC685320"	"	-8.0190"	"0.00537042"
1391426_a_at	"LOC288165"	"	-8.0187"	"0.00537379"
1392471_at	NA	"	8.0177"	"0.00538319"
1367677_at	"Prdx5"	"	-8.0174"	"0.00538648"
1373571_at	"Rtn3"	"	8.0172"	"0.00538865"
1374117_at	"Baiap2"	"	-8.0168"	"0.00539246"
1371469_at	"Chp"	"	8.0132"	"0.00542849"
1369029_at	"Plscr1"	"	8.0130"	"0.00543013"
1374897_at	"LOC690585"	"	-8.0122"	"0.00543856"

1379704_at	Zfp143	-7.4021	0.00001643
1378462_at	Mtap_predicted	7.4008	0.00001645
1367870_at	Txnl2	-7.4007	0.00001645
1375887_at	NA	-7.3981	0.00001652
1390485_at	March5_predicted	-7.3968	0.00001654
1393617_at	NA	7.3969	0.00001654
1380120_at	Mtap7_predicted	7.3933	0.00001663
1372808_at	LOC680308	-7.3915	0.00001668
1369533_a_at	Htr4	7.3878	0.00001677
1386930_at	Psmd4	-7.388	0.00001677
1374304_at	Xrcc4	7.3825	0.00001692
1375864_at	Cc2d1b	-7.3814	0.00001695
1392503_at	Rbm22	-7.3799	0.00001697
1398802_at	Ube2d3	-7.3801	0.00001697
1367876_at	Ipo13	-7.3788	0.00001699
1383307_at	MGC114379	-7.3755	0.00001708
1391333_at	Lrrfip2	7.3742	0.00001711
1378037_at	RGD1310738_predicted	-7.373	0.00001714
1383793_at	RGD1311909_predicted	7.372	0.00001716
1371490_at	Hsbp1	-7.3714	0.00001717
1388767_at	Pdcd6_predicted	-7.3674	0.00001728
1377819_at	RGD1561065_predicted	7.3662	0.00001731
1372948_at	LOC691814	-7.3629	0.00001739
1373438_at	Ube2o_predicted	-7.3633	0.00001739
1390022_at	Arpc5	-7.3618	0.0000174
1376868_at	Cobll1_predicted	7.3611	0.0000174
1368279_at	Mllt3	7.3612	0.0000174
1371941_at	RGD1310230	7.3621	0.0000174
1367824_at	Fnta	-7.3591	0.00001745
1388184_at	Icmt	-7.3567	0.0000175
1383090_at	Mrps14_predicted	-7.3565	0.0000175
1372334_at	NA	7.3575	0.0000175
1370163_at	Odc1	-7.3527	0.00001762
1372713_at	RGD1309550	7.3522	0.00001762
1374161_at	LOC683676	7.3482	0.00001773
1374897_at	LOC690585	-7.3481	0.00001773
1395750_at	NA	-7.3455	0.00001779
1376250_at	Nufip1	-7.3455	0.00001779
1374441_at	RGD1306649	-7.3421	0.00001789
1373495_at	Ube2j1_predicted	-7.3401	0.00001795
1375943_at	NA	-7.3338	0.00001815
1367991_at	Gcs1	-7.3302	0.00001826
1374516_at	RGD1306894_predicted	-7.3291	0.00001828
1389815_at	Ppp1r14b	-7.3285	0.00001829
1383625_a_at	RGD1562173_predicted	-7.3263	0.00001835
1380837_at	RGD1305846_predicted	7.3195	0.00001856
1371911_at	Tnfaip1	-7.3195	0.00001856
1372366_at	Htatip	-7.3163	0.00001866
1371064_at	Pcm1	7.315	0.00001867
1373447_at	RGD1305117	-7.3151	0.00001867

1371963_at	"Pcca"	" 8.0082"	"0.00547851"
1388715_at	"Gars"	" -8.0076"	"0.00548495"
1371715_at	"MGC112883"	" -8.0050"	"0.00551144"
1369642_at	"Pafah1b2"	" -8.0037"	"0.00552396"
1382284_at	"Nek3_predicted"	" 8.0035"	"0.00552662"
1387148_at	"Gprasp1"	" -8.0019"	"0.00554233"
1372070_at	"Ifi30"	" -8.0011"	"0.00555115"
1369130_at	"Rasgrp1"	" -7.9993"	"0.00556893"
1391250_at	NA	" -7.9961"	"0.00560258"
1399057_at	"Morf4l1"	" -7.9943"	"0.00562123"
1367605_at	"Pfn1"	" -7.9939"	"0.00562537"
1376146_at	"RGD1304587"	" -7.9922"	"0.00564271"
1388394_at	"Aars"	" -7.9907"	"0.00565778"
1388635_at	"RGD1309744_predicted"	" 7.9905"	"0.00566052"
1390628_at	"Cpeb2_predicted"	" 7.9904"	"0.00566148"
1372365_at	"Rin2_predicted"	" 7.9843"	"0.00572569"
1376761_at	NA	" 7.9821"	"0.00574825"
1382162_at	NA	" 7.9806"	"0.00576486"
1373666_at	"Rapgef5"	" 7.9772"	"0.00580074"
1371724_at	"Rexo2"	" -7.9745"	"0.00583038"
1376153_at	NA	" -7.9726"	"0.00584993"
1384479_at	"Galnt3"	" 7.9701"	"0.00587713"
1373546_at	"Ua20"	" 7.9693"	"0.00588628"
1379473_at	"RGD1307509"	" -7.9666"	"0.00591574"
1387076_at	"Hif1a"	" 7.9662"	"0.00592003"
1370017_at	"Emd"	" -7.9649"	"0.00593427"
1382117_at	"RGD1560538_predicted"	" -7.9634"	"0.00595100"
1388523_at	"Txndc12"	" -7.9623"	"0.00596291"
1372347_at	NA	" -7.9617"	"0.00596963"
1372007_at	"RGD1562975_predicted"	" -7.9605"	"0.00598314"
1368588_at	"Ddx52"	" -7.9604"	"0.00598332"
1377642_at	"Cav2"	" -7.9573"	"0.00601771"
1369933_at	"Vdac2"	" 7.9564"	"0.00602848"
1383328_x_at	"RGD1563912_predicted"	" -7.9533"	"0.00606288"
1379322_at	"Psmd7_predicted"	" -7.9509"	"0.00608932"
1393956_at	"Stk17b"	" 7.9502"	"0.00609756"
1379326_at	"RGD1305679"	" 7.9499"	"0.00610075"
1389388_at	NA	" 7.9489"	"0.00611230"
1377032_at	NA	" 7.9452"	"0.00615405"
1390514_at	"MGC94223"	" -7.9437"	"0.00617117"
1391097_at	NA	" 7.9413"	"0.00619950"
1382381_at	NA	" 7.9385"	"0.00623166"
1372873_at	"Fbxo38_predicted"	" -7.9366"	"0.00625314"
1391179_at	"Tcf4"	" -7.9355"	"0.00626596"
1397241_at	"RGD1306880_predicted"	" 7.9313"	"0.00631512"
1393193_at	"Rpusd2_predicted"	" -7.9285"	"0.00634730"
1398773_at	"Khdrbs1"	" -7.9238"	"0.00640272"
1368233_at	"Gtf2f2"	" -7.9237"	"0.00640427"
1368508_at	"Psma3"	" -7.9232"	"0.00640986"
1374118_at	"Lars"	" -7.9225"	"0.00641845"

1372888_at	Ube4a	7.3158	0.00001867
1393431_at	LOC687575	-7.3132	0.00001871
1376110_at	Rpp25	-7.3131	0.00001871
1371396_at	NA	-7.3106	0.00001878
1393377_at	Galnt4	7.3097	0.0000188
1388980_at	MGC72560	-7.3032	0.00001902
1376116_at	Ddx24	-7.3007	0.0000191
1383327_at	Pdcd4	7.2985	0.00001916
1398579_at	NA	7.2974	0.00001918
1377746_at	Rbm12	-7.2971	0.00001918
1389627_at	RGD1306209	-7.2959	0.00001921
1375480_at	RGD1566399_predicted	7.2938	0.00001928
1373380_at	RGD1562140_predicted	-7.2929	0.0000193
1387344_at	Aldh6a1	7.2919	0.00001932
1368059_at	Crym	-7.2903	0.00001935
1378138_at	Kif16b_predicted	7.2908	0.00001935
1376828_at	Gprc5a	-7.2884	0.00001941
1371951_at	Fhl2	-7.2855	0.0000195
1392901_at	LOC367113	7.2851	0.0000195
1381120_at	NA	-7.2847	0.0000195
1389009_at	Rsrc1	7.2829	0.00001956
1383594_at	LOC312863	7.28	0.00001965
1388954_at	RGD1306717_predicted	-7.2768	0.00001975
1393233_at	Armc6_predicted	-7.2755	0.00001977
1377345_at	Bbx_predicted	7.2761	0.00001977
1386097_at	NA	7.2726	0.00001987
1375205_at	Pcaf	7.2722	0.00001987
1385701_at	NA	7.2717	0.00001988
1380527_at	Pde7b	7.2705	0.00001991
1391656_at	NA	-7.2698	0.00001992
1389753_at	Brp16	-7.2632	0.00002016
1379076_at	NA	7.2586	0.00002032
1390972_at	Tprkb	-7.2571	0.00002036
1388772_at	Lsm8_predicted	-7.2558	0.00002038
1378531_at	NA	7.2561	0.00002038
1381081_at	NA	7.2547	0.00002041
1373500_at	Lrpprc	7.2473	0.00002068
1374297_at	Dync1li1	-7.2465	0.0000207
1388155_at	Krt1-18	-7.2416	0.00002086
1379422_at	LOC300284	-7.2417	0.00002086
1372126_at	Fem1b_predicted	-7.2411	0.00002087
1387113_at	Ctbp2	7.2403	0.00002089
1397558_at	NA	7.2396	0.0000209
1377377_at	Utrn	7.238	0.00002095
1381413_at	Arid1b	7.2374	0.00002096
1368588_at	Ddx52	-7.2328	0.00002113
1373647_at	Zfp622	-7.2309	0.00002119
1372130_at	Arfgef1_predicted	7.2295	0.0000212
1378536_at	NA	7.2295	0.0000212
1391573_at	Tnfrsf21_predicted	-7.2301	0.0000212

1368426_at	"Crot"	"	7.9219"	"0.00642612"
1374501_at	"RGD1309571"	"	-7.9210"	"0.00643623"
1382166_at	"Srprb"	"	-7.9199"	"0.00644967"
1388643_at	"Fut8"	"	7.9196"	"0.00645357"
1371475_at	"Rnase4"	"	-7.9179"	"0.00647328"
1373539_at	NA	"	-7.9178"	"0.00647535"
1382366_at	"Pik3ca"	"	-7.9145"	"0.00651502"
1396264_at	"LOC689116"	"	7.9106"	"0.00656180"
1384920_at	NA	"	7.9087"	"0.00658520"
1372620_at	"Anp32e"	"	-7.9012"	"0.00667715"
1372917_at	"RGD1306819"	"	-7.8999"	"0.00669324"
1375847_at	"Nudt16_predicted"	"	7.8964"	"0.00673695"
1384584_at	NA	"	7.8958"	"0.00674407"
1388609_at	"RGD1311815"	"	-7.8958"	"0.00674519"
1380668_at	"MGC72974"	"	7.8925"	"0.00678628"
1373168_at	"LOC288913"	"	-7.8885"	"0.00683683"
1390967_a_at	"RGD1306841"	"	-7.8843"	"0.00689095"
1377838_at	"RGD1562218"	"	-7.8699"	"0.00707700"
1380180_at	NA	"	7.8682"	"0.00709979"
1394940_at	"RGD1311381_predicted"	"	-7.8639"	"0.00715644"
1373590_at	"Stom"	"	-7.8587"	"0.00722712"
1392807_at	"Lig3"	"	-7.8579"	"0.00723759"
1368191_a_at	"Slc22a1"	"	7.8571"	"0.00724768"
1371983_at	"Josd1"	"	7.8535"	"0.00729631"
1381555_at	NA	"	-7.8491"	"0.00735750"
1390058_at	"LOC361990"	"	-7.8447"	"0.00741684"
1375340_at	NA	"	7.8433"	"0.00743621"
1390223_at	NA	"	-7.8349"	"0.00755388"
1376867_at	"RGD1307414_predicted"	"	-7.8348"	"0.00755565"
1390273_at	"Znf532_predicted"	"	-7.8268"	"0.00766979"
1389984_at	"LOC681740"	"	7.8262"	"0.00767844"
1388131_at	"Tubb2b"	"	-7.8261"	"0.00767979"
1389527_at	"LOC378467"	"	-7.8248"	"0.00769738"
1390049_at	"Fhl1"	"	-7.8178"	"0.00779917"
1379388_at	"Snx13_predicted"	"	7.8068"	"0.00796109"
1371506_at	NA	"	7.8066"	"0.00796483"
1374483_at	NA	"	-7.8063"	"0.00796805"
1371951_at	"Fhl2"	"	-7.8037"	"0.00800743"
1388725_at	"Lepr"	"	7.8027"	"0.00802184"
1391238_at	"Gmds"	"	7.8009"	"0.00804923"
1392909_at	NA	"	-7.7981"	"0.00809233"
1373329_at	"Tmprss2"	"	-7.7933"	"0.00816430"
1372918_at	"RGD1305898_predicted"	"	-7.7925"	"0.00817656"
1378068_at	NA	"	-7.7866"	"0.00826706"
1385458_a_at	"RGD1306959_predicted"	"	-7.7844"	"0.00830192"
1397892_at	"Eif1a"	"	-7.7840"	"0.00830776"
1369626_at	"Ide"	"	7.7827"	"0.00832750"
1390827_at	"Smad3"	"	-7.7825"	"0.00833098"
1387782_at	"Dynll2"	"	-7.7812"	"0.00835172"
1392465_at	"Sap18"	"	-7.7798"	"0.00837285"

1378705_at	RGD1310433_predicted	7.229	0.00002121
1373215_at	Abr_predicted	7.2265	0.00002128
1373502_at	Dym_predicted	7.2268	0.00002128
1394460_at	NA	7.2258	0.00002128
1384310_at	NA	7.2259	0.00002128
1380544_at	NA	7.2214	0.00002143
1390502_at	RGD1309104_predicted	7.2209	0.00002143
1388695_at	Shmt2	-7.2218	0.00002143
1389172_at	NA	7.2196	0.00002147
1368247_at	Hspa1a	-7.2188	0.00002148
1377021_at	RGD1308877_predicted	-7.2189	0.00002148
1372534_at	Bhmt	-7.2156	0.00002155
1373189_at	Mkl1_predicted	7.2157	0.00002155
1383232_at	NA	-7.2166	0.00002155
1373690_at	NA	-7.2156	0.00002155
1396386_at	Bace2	-7.2141	0.00002159
1387780_at	Dnaja2	-7.2141	0.00002159
1377968_at	NA	7.2129	0.00002162
1373866_at	RGD1359509	-7.2125	0.00002162
1370925_at	LOC291411	7.2117	0.00002163
1376168_at	Tmem32_predicted	-7.2116	0.00002163
1374293_at	LOC690795	-7.211	0.00002164
1398781_at	Atp6v1f	-7.2079	0.00002174
1375425_at	RGD1309400_predicted	-7.2082	0.00002174
1388861_at	RGD1307896_predicted	-7.206	0.0000218
1380067_at	Myo5c_predicted	7.2047	0.00002184
1376862_at	Ube4b_predicted	7.2042	0.00002185
1391454_at	RGD1565584_predicted	-7.2026	0.0000219
1398916_at	Aurkaip1	-7.2009	0.00002194
1383896_at	LOC683504	-7.201	0.00002194
1373113_at	NA	7.1953	0.00002216
1383466_at	NA	-7.194	0.00002217
1373588_at	RGD1310323	-7.1945	0.00002217
1379245_at	Txndc9	-7.1945	0.00002217
1389491_at	NA	7.192	0.00002224
1371486_at	Snrp1c_predicted	-7.1904	0.00002229
1374462_at	Kifap3_predicted	7.1883	0.00002237
1393584_at	Tnfrsf21_predicted	-7.188	0.00002237
1372036_at	Cd2bp2_predicted	-7.1865	0.00002242
1371682_at	Map1lc3a	-7.1811	0.0000226
1384122_at	NA	-7.1814	0.0000226
1375751_at	NA	-7.1812	0.0000226
1377720_x_at	NA	7.1782	0.00002271
1395696_at	RGD1306613_predicted	-7.1757	0.0000228
1384871_at	Osbpl6_predicted	7.1742	0.00002285
1371002_at	Pdcd2	-7.1707	0.00002299
1388399_at	Mast2_predicted	7.1701	0.000023
1389983_at	RGD1309387	-7.1675	0.0000231
1379257_at	Epb4.1l4a_predicted	7.165	0.00002318
1393079_at	Siae_predicted	7.1652	0.00002318

1384032_at	NA	"	-7.7786"	"0.00839283"
1388108_at	"Elovl6"	"	7.7763"	"0.00842870"
1368012_at	"Tep1"	"	7.7760"	"0.00843418"
1395802_at	"LOC685144"	"	-7.7720"	"0.00849722"
1394808_at	"Tm9sf4"	"	-7.7719"	"0.00849847"
1371434_at	NA	"	-7.7712"	"0.00850929"
1373695_at	NA	"	-7.7702"	"0.00852478"
1382811_at	NA	"	-7.7692"	"0.00854200"
1374326_at	"Ppan"	"	-7.7678"	"0.00856447"
1376001_at	"Praf1_predicted"	"	-7.7627"	"0.00864642"
1390799_at	NA	"	7.7602"	"0.00868672"
1374699_at	"RGD1305779_predicted"	"	-7.7588"	"0.00870971"
1376974_at	"Ttc7"	"	7.7575"	"0.00873050"
1373182_at	NA	"	7.7559"	"0.00875755"
1374129_at	"RGD1562933_predicted"	"	7.7547"	"0.00877638"
1388389_at	"Sept2"	"	-7.7533"	"0.00879949"
1378092_at	"RGD1311147_predicted"	"	7.7512"	"0.00883451"
1373848_at	"RGD1308584_predicted"	"	-7.7509"	"0.00883931"
1375895_at	NA	"	-7.7495"	"0.00886243"
1388622_at	"RGD1563521_predicted"	"	-7.7448"	"0.00894153"
1372815_at	"Magoh_predicted"	"	-7.7424"	"0.00898120"
1370300_at	"Preb"	"	-7.7412"	"0.00900182"
1389472_at	"RGD1311526_predicted"	"	-7.7388"	"0.00904273"
1378634_at	"Hdac8_predicted"	"	7.7359"	"0.00909193"
1368249_at	"Klf15"	"	7.7342"	"0.00912103"
1388155_at	"Krt1-18"	"	-7.7338"	"0.00912833"
1373052_at	"Pdcl3"	"	-7.7336"	"0.00913216"
1377703_at	"RGD1310316"	"	-7.7308"	"0.00918060"
1374040_at	"Nek9_predicted"	"	-7.7276"	"0.00923530"
1374491_at	"Cmtm8"	"	7.7261"	"0.00926059"
1398903_at	"Esd"	"	7.7218"	"0.00933611"
1388424_at	"Eif3s1_predicted"	"	-7.7178"	"0.00940603"
1378898_at	"Ddx19"	"	-7.7170"	"0.00942102"
1369854_a_at	"Ceacam1"	"	-7.7144"	"0.00946737"
1386162_at	"Txk"	"	-7.7121"	"0.00950852"
1372199_at	"Pink1_predicted"	"	7.7106"	"0.00953581"
1388586_at	"Synj1"	"	7.7105"	"0.00953694"
1397772_at	NA	"	-7.7103"	"0.00954081"
1379801_at	NA	"	7.7075"	"0.00959153"
1371505_at	"Hnrpc"	"	-7.7069"	"0.00960267"
1372743_at	"Snx5_predicted"	"	7.7059"	"0.00961944"
1380835_at	"Isg20l2"	"	-7.7038"	"0.00965797"
1383469_at	NA	"	-7.7022"	"0.00968694"
1380030_at	"Znf593_predicted"	"	-7.7016"	"0.00969759"
1374082_at	"RGD1311578"	"	-7.6978"	"0.00976694"
1398837_at	"Tceb2"	"	-7.6974"	"0.00977561"
1371188_a_at	"Ubtf"	"	-7.6964"	"0.00979309"
1392858_at	"LOC689116"	"	7.6953"	"0.00981297"
1372262_at	NA	"	7.6910"	"0.00989402"
1373337_at	"Grhpr_predicted"	"	7.6883"	"0.00994477"

1391476_at	NA	7.1637	0.00002322
1374245_at	LOC363251	7.1604	0.00002335
1398960_at	Cct6a	-7.1592	0.00002337
1372794_at	Dapk2	7.1586	0.00002337
1372607_at	Nubp2	-7.1585	0.00002337
1377474_at	RGD1308317_predicted	-7.1583	0.00002337
1382114_at	Tlk1_predicted	7.1589	0.00002337
1371546_at	LOC361128	-7.1567	0.00002342
1394086_at	Senp7_predicted	7.1537	0.00002354
1372469_at	LOC686310	7.1525	0.00002359
1371764_at	MGC94113	-7.1518	0.0000236
1389722_at	NA	7.1514	0.0000236
1388444_at	Ubxd2	-7.1499	0.00002365
1371488_at	RGD1562079_predicted	-7.1493	0.00002367
1387775_at	Gtf2a2	-7.1483	0.00002369
1379811_at	Pde4dip	7.1481	0.00002369
1367698_a_at	40057	7.145	0.00002381
1388488_at	Lsm3_predicted	-7.1448	0.00002381
1379429_at	Tmed5	-7.1439	0.00002383
1376852_at	Mccc1	7.1417	0.00002392
1398819_at	Dnaja1	-7.1398	0.00002399
1387116_at	Dnajb9	-7.1381	0.00002405
1377577_at	Gmps	-7.1328	0.00002425
1373854_at	NA	7.1334	0.00002425
1369642_at	Pafah1b2	-7.1331	0.00002425
1371399_at	LOC681123	-7.1285	0.00002443
1389372_at	RGD1308489_predicted	7.1268	0.00002449
1368936_at	Txnl1	-7.1263	0.0000245
1372619_at	Mrpl49	-7.1247	0.00002456
1372119_at	Ube1dc1	-7.1241	0.00002457
1372266_at	Rev3l	7.123	0.00002461
1373382_at	RGD1306324	-7.1202	0.00002473
1374122_at	Myo5c_predicted	7.1196	0.00002474
1383151_at	Akap9	7.1168	0.00002485
1375357_at	Dyt1	-7.1115	0.00002509
1398369_at	NA	7.1105	0.00002512
1370316_at	Hspbp1	-7.1097	0.00002513
1377696_at	Mesdc1	-7.1096	0.00002513
1367517_at	NA	7.1089	0.00002515
1377576_at	Ppa2_predicted	7.1051	0.00002531
1389216_at	NA	7.1039	0.00002536
1371027_at	Cblb	7.1024	0.00002539
1395488_at	Msh3	7.1028	0.00002539
1388865_at	Ppp4r2_predicted	-7.1018	0.00002539
1386905_at	Prkar1a	-7.1021	0.00002539
1388983_at	RGD1305045_predicted	-7.1009	0.00002542
1397706_at	NA	7.0992	0.00002547
1372917_at	RGD1306819	-7.0997	0.00002547
1377649_at	NA	7.0967	0.00002558
1389529_at	NA	7.0962	0.00002559

1379335_at	"Paip1_predicted"	"	-7.6881"	"0.00994880"
1387833_at	"Tmprss2"	"	-7.6832"	"0.01003968"
1390962_at	NA	"	-7.6744"	"0.01020744"
1376916_at	"Zbtb2_predicted"	"	-7.6720"	"0.01025460"
1392853_at	NA	"	-7.6656"	"0.01037998"
1383138_at	"Ssh3"	"	-7.6613"	"0.01046347"
1392902_at	"RGD1306067"	"	-7.6599"	"0.01049231"
1377757_at	"Shprh_predicted"	"	-7.6578"	"0.01053328"
1367713_at	"Eif2s1"	"	-7.6566"	"0.01055745"
1377779_at	"Pdcl3"	"	-7.6565"	"0.01055858"
1391016_at	"LOC690654"	"	7.6545"	"0.01059816"
1383265_at	"Tbc1d23_predicted"	"	7.6545"	"0.01060008"
1395036_at	"Impad1"	"	-7.6529"	"0.01063179"
1388720_at	NA	"	7.6493"	"0.01070425"
1389428_at	"F10"	"	-7.6425"	"0.01084276"
1372364_a_at	"RGD1305160"	"	7.6421"	"0.01085015"
1399122_at	NA	"	7.6412"	"0.01086991"
1372787_at	"Crlz1"	"	-7.6407"	"0.01087968"
1371455_at	"Pmm1"	"	7.6387"	"0.01092083"
1376351_at	NA	"	-7.6350"	"0.01099747"
1398421_at	NA	"	7.6340"	"0.01101746"
1367714_at	"Eif2b2"	"	-7.6334"	"0.01102952"
1386879_at	"Lgals3"	"	7.6326"	"0.01104677"
1395146_at	"Adipor2"	"	-7.6321"	"0.01105688"
1373164_at	NA	"	7.6319"	"0.01106147"
1391636_at	NA	"	-7.6305"	"0.01109146"
1367613_at	"Prdx1"	"	-7.6303"	"0.01109611"
1383712_at	"Rxrg"	"	-7.6272"	"0.01116062"
1369562_at	"Hpcal1"	"	-7.6252"	"0.01120370"
1376519_at	"Brp16"	"	-7.6240"	"0.01122927"
1376679_at	NA	"	-7.6216"	"0.01127864"
1397535_at	"Mpp5_predicted"	"	7.6210"	"0.01129261"
1373768_at	"Bms1l"	"	-7.6205"	"0.01130382"
1367798_at	"Ahcy"	"	7.6199"	"0.01131618"
1373653_at	"Papd1_predicted"	"	-7.6194"	"0.01132742"
1396933_s_at	"LOC191574"	"	7.6185"	"0.01134702"
1378080_at	"RGD1559538_predicted"	"	7.6178"	"0.01136127"
1398300_at	"Atp1b3"	"	-7.6138"	"0.01144851"
1378930_a_at	"Rhoc_predicted"	"	7.6134"	"0.01145535"
1373928_at	"Il17re"	"	7.6134"	"0.01145666"
1380777_at	"Tsc22d3"	"	-7.6054"	"0.01163137"
1367883_at	"Smn1"	"	-7.6037"	"0.01166887"
1393502_at	"RGD1306153"	"	-7.6037"	"0.01166928"
1374133_at	"Trit1_predicted"	"	-7.6013"	"0.01172226"
1387925_at	"Asns"	"	-7.6007"	"0.01173499"
1376562_at	"RGD1561817_predicted"	"	7.5966"	"0.01182694"
1372943_at	"LOC497952"	"	-7.5963"	"0.01183351"
1387388_at	"Chp"	"	7.5954"	"0.01185377"
1376242_at	NA	"	-7.5953"	"0.01185530"
1388562_at	"Stard7_predicted"	"	7.5927"	"0.01191570"

1367493_at	RGD1560212_predicted	-7.0955	0.00002561
1371812_at	LOC362855	-7.0921	0.00002575
1372760_at	NA	7.0917	0.00002576
1392571_at	RGD1311340_predicted	7.0879	0.00002593
1378900_at	Gcn5l2_predicted	-7.0825	0.00002618
1374797_at	Eaf1_predicted	-7.0808	0.00002625
1378068_at	NA	-7.0802	0.00002626
1369686_at	Dcamkl1	7.0793	0.00002629
1392885_at	Mbd1	-7.0777	0.00002635
1382310_at	RGD1563296_predicted	7.0772	0.00002637
1376585_at	Mrpl50_predicted	-7.0758	0.0000264
1373814_at	RGD1310066	7.0758	0.0000264
1386065_at	NA	-7.0729	0.00002652
1389550_at	Sh3gl2	7.0729	0.00002652
1390461_at	NA	7.0706	0.00002661
1374931_at	Mrpl40	-7.0701	0.00002663
1376708_at	LOC680422	7.0686	0.00002667
1374169_at	RGD1310686	7.0686	0.00002667
1385804_x_at	LOC688637	-7.068	0.00002668
1384256_at	LOC686883	-7.0663	0.0000267
1375423_at	LOC689959	-7.0667	0.0000267
1399140_at	Rapgef1	7.0672	0.0000267
1392504_at	RGD1306613_predicted	-7.0669	0.0000267
1372076_at	Hbxip_predicted	-7.0621	0.0000269
1388903_at	Tcte1l	-7.0571	0.00002714
1378007_at	RGD1306148_predicted	7.0559	0.00002719
1388829_at	LOC361399	-7.0535	0.00002726
1374368_at	NA	7.0538	0.00002726
1388567_at	Thumpd1	-7.0534	0.00002726
1376731_at	NA	7.0528	0.00002728
1379217_at	NA	7.0511	0.00002735
1371809_at	Mrps18b	-7.0495	0.00002742
1375944_at	Acss2_predicted	7.0483	0.00002747
1397508_at	Ddx18	-7.0454	0.0000276
1379982_at	NA	7.0437	0.00002767
1383933_at	RGD1308772_predicted	7.0421	0.00002774
1383692_at	LOC681037	7.0394	0.00002784
1377400_at	NA	-7.0399	0.00002784
1387152_at	Nrbf2	-7.0392	0.00002784
1372364_a_at	RGD1305160	-7.039	0.00002784
1368067_at	Zfp148	7.0381	0.00002787
1397924_at	NA	7.035	0.00002802
1379153_at	LOC362587	-7.0345	0.00002803
1374483_at	NA	-7.0331	0.00002808
1396752_at	Tnfrsf21_predicted	-7.0321	0.00002812
1391439_at	NA	7.0314	0.00002814
1385020_at	LOC499339	-7.0299	0.00002821
1370939_at	Acsl1	7.0293	0.00002822
1370386_at	Ruvbl1	-7.0237	0.00002851
1372665_at	Psat1	-7.0226	0.00002855

1396189_at	NA	" -7.5926"	"0.01191692"
1395642_at	"Nol9"	" -7.5924"	"0.01192211"
1369777_a_at	"Shank2"	" 7.5922"	"0.01192694"
1367603_at	"Tpi1"	" 7.5920"	"0.01193110"
1387058_at	"Pctp"	" 7.5909"	"0.01195566"
1384087_at	"RGD1561402_predicted"	" -7.5908"	"0.01195890"
1381762_at	"RGD1309120"	" 7.5894"	"0.01198979"
1371019_at	"Trib1"	" -7.5887"	"0.01200513"
1378887_at	NA	" -7.5884"	"0.01201237"
1398084_at	"LOC501026"	" -7.5867"	"0.01205088"
1395508_at	"Cct5"	" -7.5837"	"0.01212057"
1371447_at	"Plac8_predicted"	" -7.5833"	"0.01212881"
1379365_at	"Cxcl11"	" 7.5826"	"0.01214539"
1367543_at	"LOC685079"	" -7.5800"	"0.01220495"
1389559_at	NA	" 7.5786"	"0.01223848"
1388251_at	"Prkci"	" 7.5766"	"0.01228590"
1398299_at	"Arhgef11"	" 7.5760"	"0.01229992"
1372372_at	"RGD1306952"	" 7.5731"	"0.01236684"
1392916_at	"Mtap7_predicted"	" 7.5707"	"0.01242411"
1383999_at	NA	" 7.5694"	"0.01245387"
1377480_at	"RGD1308076"	" -7.5644"	"0.01257370"
1368203_at	"Scnn1a"	" -7.5627"	"0.01261456"
1388294_at	"Sdhd"	" 7.5622"	"0.01262489"
1394630_at	"Lmo4"	" -7.5610"	"0.01265331"
1383516_at	"Fgl2"	" 7.5608"	"0.01265972"
1376111_at	NA	" 7.5605"	"0.01266544"
1374254_a_at	"RGD1563250_predicted"	" 7.5597"	"0.01268671"
1389040_at	NA	" -7.5571"	"0.01274828"
1372741_at	"Sccpdh"	" 7.5560"	"0.01277559"
1368538_at	"Exoc7"	" -7.5559"	"0.01277901"
1376592_at	"Mcee_predicted"	" 7.5537"	"0.01283208"
1388611_at	"Tcea3"	" 7.5531"	"0.01284535"
1394205_at	"Slk"	" -7.5527"	"0.01285513"
1382536_at	"Thrap2_predicted"	" 7.5447"	"0.01305369"
1398798_at	"Metap2"	" -7.5417"	"0.01312864"
1387950_at	"Nip7"	" -7.5393"	"0.01318692"
1367519_at	"Osbpl2"	" -7.5393"	"0.01318895"
1390838_at	NA	" 7.5389"	"0.01319733"
1368029_at	"Gnai3"	" -7.5330"	"0.01334647"
1371999_at	NA	" -7.5312"	"0.01339368"
1388957_at	NA	" -7.5309"	"0.01340118"
1388889_at	"Fxna"	" 7.5283"	"0.01346650"
1372633_at	"Spg20"	" -7.5274"	"0.01349023"
1398554_at	"Atp6v0b_predicted"	" -7.5221"	"0.01362641"
1367487_at	"B4galt3"	" -7.5208"	"0.01366076"
1398913_at	"Numa1"	" 7.5194"	"0.01369901"
1391162_at	NA	" 7.5183"	"0.01372691"
1389809_at	NA	" -7.5177"	"0.01374182"
1387344_at	"Aldh6a1"	" 7.5171"	"0.01375778"
1380242_at	"Lrp5_predicted"	" 7.5148"	"0.01381758"

1378031_at	Dhx37_predicted	-7.0211	0.00002862
1388201_at	Bmp6	7.0177	0.00002878
1389004_at	Josd2_predicted	-7.0164	0.00002884
1399167_a_at	NA	7.0127	0.00002902
1376322_at	Sfxn5	7.0103	0.00002914
1398415_at	NA	7.009	0.00002919
1374152_at	Wdr46	-7.0087	0.00002919
1378590_at	NA	7.0042	0.00002942
1379043_at	NA	-7.0039	0.00002943
1373875_at	RGD1308261_predicted	-7.0033	0.00002944
1371445_at	Lrrc59	-7.0027	0.00002946
1399113_at	NA	7.0017	0.0000295
1388339_at	Pea15	-7.0009	0.00002953
1372721_at	Zfpn1a2_predicted	-6.9998	0.00002957
1374241_at	NA	6.9984	0.00002963
1375525_at	Mapre1	-6.9974	0.00002966
1370354_at	Parg	6.9973	0.00002966
1393310_at	NA	6.9931	0.00002988
1379263_at	Fkrp	-6.9921	0.00002992
1392573_at	Ube2a	-6.9911	0.00002996
1398860_at	Nedd8	-6.9902	0.00002999
1377619_at	LOC688637	-6.9857	0.00003023
1386900_at	RAMP4	-6.9835	0.00003034
1373546_at	Ua20	6.9827	0.00003037
1367960_at	Arl4a	-6.9822	0.00003038
1378070_at	NA	-6.9814	0.00003041
1390343_at	Ccnc	-6.9808	0.00003043
1384479_at	Galnt3	-6.9801	0.00003045
1393066_at	Cdh1	6.9791	0.00003049
1374639_at	LOC287522	6.976	0.00003065
1377937_at	Mrps14_predicted	-6.9744	0.00003071
1385667_x_at	NA	6.9748	0.00003071
1388830_at	Pkn2	6.974	0.00003072
1374000_at	Helz_predicted	6.9733	0.00003073
1374316_at	LOC686980	-6.9732	0.00003073
1372705_at	Cherp_predicted	-6.9724	0.00003076
1388677_at	Ubap1	-6.9712	0.00003081
1368341_at	Polb	-6.97	0.00003083
1388466_at	Psmd3	-6.9707	0.00003083
1382729_at	RGD1564019_predicted	6.9702	0.00003083
1372543_at	RGD1562502_predicted	-6.9659	0.00003105
1371653_at	Tpm4	-6.964	0.00003115
1384296_at	NA	6.9623	0.00003121
1389199_at	RGD1309079	-6.9625	0.00003121
1371449_at	Pin1_predicted	-6.9619	0.00003122
1367686_at	RAMP4	-6.9592	0.00003136
1382944_at	Igsf6	6.9566	0.00003148
1373312_at	NA	6.9568	0.00003148
1372433_at	RGD1310211_predicted	-6.9556	0.0000315
1388346_at	RGD1560953_predicted	-6.9557	0.0000315

1391131_at	NA	" -7.5136"	"0.01384966"
1386904_a_at	"Cyb5"	" 7.5119"	"0.01389413"
1394535_at	"Kif16b_predicted"	" 7.5092"	"0.01396774"
1389153_at	"Serbp1"	" -7.5073"	"0.01401805"
1378208_at	NA	" 7.5051"	"0.01407819"
1392514_at	"Bxdc1_predicted"	" -7.5047"	"0.01408840"
1398932_at	"LOC690660"	" 7.5025"	"0.01414635"
1375889_at	"Sms"	" -7.5023"	"0.01415134"
1371483_at	"Nnt"	" -7.5000"	"0.01421503"
1379286_at	NA	" 7.5000"	"0.01421560"
1373765_at	NA	" 7.4974"	"0.01428492"
1374484_at	"Tmem39a"	" -7.4925"	"0.01441875"
1372218_at	"Wdr12"	" -7.4924"	"0.01442218"
1383667_at	"Rundc1_predicted"	" -7.4920"	"0.01443286"
1368509_at	"Bbs2"	" 7.4910"	"0.01446018"
1387087_at	"Cebpb"	" -7.4887"	"0.01452545"
1369644_at	"Lphn2"	" -7.4879"	"0.01454681"
1389961_at	"Sdccag3"	" -7.4869"	"0.01457618"
1368055_a_at	"Lmna"	" -7.4854"	"0.01461623"
1372722_at	NA	" -7.4837"	"0.01466479"
1388933_at	NA	" 7.4818"	"0.01471757"
1391193_at	"RGD1560656_predicted"	" -7.4763"	"0.01487291"
1372039_at	"Nkiras2_predicted"	" 7.4743"	"0.01493080"
1391739_at	"Rundc1_predicted"	" -7.4707"	"0.01503401"
1387797_at	"Rab7"	" 7.4698"	"0.01506095"
1377307_at	"RGD1309879"	" 7.4690"	"0.01508181"
1379264_at	"Znrf1_predicted"	" 7.4678"	"0.01511806"
1373733_at	"Bok"	" 7.4672"	"0.01513564"
1389608_at	"Abcf2_predicted"	" -7.4666"	"0.01515354"
1396160_at	NA	" -7.4654"	"0.01518638"
1391378_at	"Lpin2_predicted"	" 7.4622"	"0.01528013"
1389683_at	NA	" -7.4614"	"0.01530266"
1378181_at	"Rpp40"	" -7.4602"	"0.01533857"
1393572_at	"Zfp592_predicted"	" -7.4566"	"0.01544507"
1394738_at	NA	" 7.4545"	"0.01550716"
1370191_at	"Azin1"	" -7.4540"	"0.01552235"
1382756_at	"Kpna1"	" -7.4531"	"0.01554795"
1376043_at	"MGC94192"	" -7.4509"	"0.01561418"
1388502_at	"Inpp5b"	" 7.4487"	"0.01568100"
1373690_at	NA	" -7.4465"	"0.01574685"
1370321_at	"Pdcd8"	" 7.4461"	"0.01575834"
1376655_at	NA	" -7.4432"	"0.01584769"
1392099_at	"Plcl3_predicted"	" 7.4414"	"0.01590077"
1385660_at	"RGD1309059_predicted"	" -7.4392"	"0.01596775"
1373647_at	"Zfp622"	" -7.4379"	"0.01600830"
1395704_at	NA	" 7.4357"	"0.01607791"
1384873_at	"Stim1_predicted"	" -7.4330"	"0.01616131"
1370563_at	"LOC191574"	" 7.4281"	"0.01631327"
1371419_at	"Spnb2"	" 7.4275"	"0.01633043"
1371706_at	"Sdccag3"	" -7.4227"	"0.01648227"

1398342_at	LOC501052	-6.9546	0.00003153
1390866_at	NA	-6.9548	0.00003153
1374602_at	Tspyl	-6.9542	0.00003154
1374958_at	Adpgk	-6.9535	0.00003156
1389022_at	Fxna	6.9529	0.00003158
1388842_at	Srf_predicted	-6.952	0.00003161
1376418_a_at	Iars_predicted	-6.9515	0.00003163
1380219_at	Hmgn2	-6.9475	0.00003183
1393689_at	Ndufaf1_predicted	-6.9475	0.00003183
1371963_at	Pcca	6.9473	0.00003183
1388325_at	Atp6v1d	-6.9423	0.00003211
1373888_at	Ap3b1_predicted	6.941	0.00003218
1374594_at	LOC363060	-6.9397	0.00003224
1383160_at	Chordc1_predicted	-6.9367	0.0000324
1377632_at	LOC680130	6.9346	0.00003251
1388986_at	NA	-6.9325	0.00003261
1390268_at	Rab35	-6.9328	0.00003261
1395721_at	Papss2_predicted	6.932	0.00003262
1398770_at	Rpl36a	-6.9315	0.00003264
1380975_at	RGD1564074_predicted	6.9294	0.00003275
1388401_at	Flnb_predicted	6.9269	0.00003289
1376651_at	NA	6.9232	0.0000331
1373364_at	Eif4g3_predicted	6.9202	0.00003323
1367672_at	Hsd17b4	6.9205	0.00003323
1383063_a_at	NA	6.9207	0.00003323
1378906_at	RGD1559690_predicted	-6.9144	0.00003358
1378345_at	NA	-6.9133	0.00003363
1374935_at	NA	6.9132	0.00003363
1387126_at	Atp2c1	6.9098	0.00003383
1383271_at	Ccdc59_predicted	-6.9085	0.00003389
1397210_at	LOC679869	6.9065	0.00003398
1373895_at	NA	-6.9067	0.00003398
1398771_at	Slc3a2	-6.905	0.00003406
1389137_at	Cit	6.9025	0.00003421
1392083_at	LOC299907	6.902	0.00003422
1370171_at	Hnrpu	-6.9004	0.00003425
1389733_at	NA	-6.9003	0.00003425
1372962_at	Tarbp2	-6.9008	0.00003425
1383949_at	Thrap2_predicted	6.9007	0.00003425
1389080_at	Rbm14	-6.9	0.00003426
1391411_at	RGD1560248_predicted	6.8987	0.00003433
1371857_at	Kctd10	-6.897	0.00003442
1377787_at	RGD1560367_predicted	6.8966	0.00003442
1398946_at	LOC688912	-6.8962	0.00003443
1386982_at	Mgat2	-6.8951	0.00003449
1382387_at	Tmem16a_predicted	6.8947	0.00003449
1372506_at	Psme3	-6.8922	0.00003463
1384056_at	NA	6.8909	0.0000347
1388468_at	Cdc42se1	-6.8898	0.00003476
1387448_at	Bet1l	-6.8869	0.00003493

1378524_at	"Rnf19_predicted"	"	-7.4190"	"0.01660130"
1394509_at	NA	"	-7.4184"	"0.01661970"
1379897_at	NA	"	-7.4177"	"0.01664334"
1377400_at	"Hrb2_predicted"	"	-7.4124"	"0.01681151"
1392971_at	NA	"	-7.4085"	"0.01694018"
1372121_at	"Usp9x_predicted"	"	7.4068"	"0.01699520"
1372306_at	"Ethe1_predicted"	"	7.4062"	"0.01701541"
1380431_at	"RGD1308772_predicted"	"	7.4048"	"0.01706097"
1375493_at	"Vangl2_predicted"	"	-7.4041"	"0.01708138"
1387901_at	"Ptprd"	"	-7.4028"	"0.01712588"
1388465_at	"RGD1359600"	"	7.4027"	"0.01712797"
1390392_at	"RGD1309602_predicted"	"	-7.4025"	"0.01713419"
1383159_at	NA	"	7.4025"	"0.01713669"
1392116_at	"RGD1311316"	"	-7.4022"	"0.01714589"
1374232_at	"Pik3ca"	"	-7.4006"	"0.01719699"
1388798_at	"Ube2e2"	"	7.3984"	"0.01726965"
1387566_at	"Pla2g4a"	"	7.3984"	"0.01727263"
1395325_s_at	"Tmem32_predicted"	"	-7.3972"	"0.01730949"
1387407_at	"Nap1l3"	"	-7.3956"	"0.01736509"
1368778_at	"Slc6a6"	"	7.3947"	"0.01739408"
1391183_at	"Gopc_predicted"	"	-7.3901"	"0.01754864"
1376502_at	"RGD1309228"	"	-7.3866"	"0.01766896"
1389450_at	"LOC368084"	"	-7.3853"	"0.01771143"
1370648_a_at	"Cr16"	"	7.3841"	"0.01775156"
1372853_at	"Rela"	"	-7.3810"	"0.01786024"
1368184_at	"Psmd9"	"	-7.3800"	"0.01789422"
1378840_at	NA	"	-7.3786"	"0.01794276"
1384143_at	"Cgn_predicted"	"	7.3772"	"0.01798909"
1369182_at	"F3"	"	-7.3760"	"0.01803307"
1389409_at	"LOC500040"	"	-7.3741"	"0.01809905"
1387120_at	"Psmc3"	"	-7.3717"	"0.01818368"
1398955_at	"Cops8"	"	-7.3695"	"0.01825881"
1383548_at	"Ankrd49_predicted"	"	-7.3686"	"0.01829285"
1371596_at	"Rnps1"	"	-7.3678"	"0.01831840"
1370876_at	"LOC681287"	"	-7.3671"	"0.01834492"
1376304_at	NA	"	-7.3664"	"0.01836768"
1382660_at	"RGD1563084_predicted"	"	7.3661"	"0.01837900"
1372552_at	"Acbd3"	"	-7.3655"	"0.01840057"
1368121_at	"Akr7a3"	"	7.3644"	"0.01844088"
1380241_a_at	"Lrp5_predicted"	"	7.3641"	"0.01845020"
1396279_at	"Atp6ap2"	"	-7.3625"	"0.01850862"
1390022_at	"Arpc5"	"	-7.3620"	"0.01852396"
1380120_at	"Mtap7_predicted"	"	7.3617"	"0.01853563"
1387194_at	"Centa1"	"	7.3594"	"0.01861847"
1387502_at	"Stk17b"	"	7.3580"	"0.01866855"
1371344_at	"RGD1562402_predicted"	"	-7.3562"	"0.01873230"
1372496_at	"RGD1561264_predicted"	"	-7.3520"	"0.01888584"
1388937_at	"Rnf19_predicted"	"	-7.3510"	"0.01892304"
1382220_at	"RGD1305614_predicted"	"	7.3467"	"0.01907911"
1377926_at	"Centg2_predicted"	"	7.3459"	"0.01910922"

1375278_at	Trim2	6.8865	0.00003494
1372453_at	Dr1	-6.8839	0.00003509
1397729_x_at	LOC363060	-6.8836	0.00003509
1371681_at	RGD708449	-6.8834	0.00003509
1376771_at	NA	6.8816	0.00003519
1398336_at	Rnf25	-6.8814	0.00003519
1398300_at	Atp1b3	-6.8808	0.0000352
1375353_at	Cables1_predicted	6.8805	0.00003521
1367526_at	Mrps26_predicted	-6.8797	0.00003524
1371585_at	Gspt1	-6.8785	0.0000353
1393983_at	Xpot_predicted	-6.8776	0.00003534
1398889_at	Grinl1a	-6.8762	0.00003542
1389065_at	Rbm34	-6.8759	0.00003542
1390728_at	Limd1_predicted	-6.8742	0.0000355
1373242_at	LOC689030	-6.8744	0.0000355
1391060_at	NA	6.8738	0.00003551
1391710_at	Nme2	-6.8734	0.00003551
1374105_at	Hig1	-6.8713	0.00003564
1389498_at	Tysnd1_predicted	-6.8704	0.00003568
1398833_at	Mbtps1	6.8692	0.00003574
1383889_at	NA	-6.8679	0.00003581
1371348_at	Psmb5	-6.8603	0.00003632
1387030_at	Abcc5	6.8585	0.00003642
1379238_at	Ctdspl_predicted	6.8571	0.0000365
1372502_at	NA	6.8542	0.00003668
1387982_at	Tlr4	-6.8508	0.00003691
1374920_at	NA	6.8495	0.00003698
1394397_at	LOC302898	-6.8477	0.00003709
1371650_at	Senp3	-6.8399	0.00003762
1377096_at	Mtfmt	6.8391	0.00003766
1399106_at	LOC681389	-6.8376	0.00003775
1382565_at	RGD1311455_predicted	-6.8348	0.00003794
1373094_at	Gtf2h1_predicted	-6.834	0.00003797
1383906_at	LOC316326	-6.8307	0.00003813
1378552_at	Map3k6_predicted	-6.8308	0.00003813
1398913_at	Numa1	6.831	0.00003813
1374716_at	RGD1306106	-6.8314	0.00003813
1374960_at	RGD1564887_predicted	6.828	0.0000383
1395991_at	Rimbp2	6.8278	0.0000383
1376580_at	Fxn_predicted	-6.825	0.00003849
1388722_at	Dnajb1_predicted	-6.8185	0.00003893
1368018_at	Mkln1	6.8183	0.00003893
1383239_at	Zcchc7_predicted	6.8183	0.00003893
1374793_at	Wdr3_predicted	-6.815	0.00003916
1368005_at	Itpr3	6.8122	0.00003934
1398951_at	RGD1308009	6.8098	0.0000395
1377622_at	Hbxap_predicted	6.8076	0.00003965
1373657_at	Slc31a2	-6.805	0.00003983
1370190_at	H3f3b	-6.8038	0.00003986
1395887_at	LOC363060	-6.8037	0.00003986

1374629_at	"Med8_predicted"	"	-7.3455"	"0.01912620"
1370708_a_at	"LOC191574"	"	7.3439"	"0.01918578"
1373603_at	"RGD1565744_predicted"	"	-7.3426"	"0.01923407"
1372883_at	"Cenpb_predicted"	"	-7.3423"	"0.01924251"
1373263_at	"LOC690257"	"	-7.3423"	"0.01924348"
1372370_at	"Rpusd4"	"	-7.3417"	"0.01926714"
1390132_at	NA	"	7.3407"	"0.01930388"
1388576_at	"Eif3s9"	"	-7.3315"	"0.01965102"
1391316_at	NA	"	7.3276"	"0.01979652"
1398326_at	"MGC105647"	"	7.3269"	"0.01982376"
1386978_at	"Bnip3l"	"	7.3262"	"0.01985042"
1373560_at	"RGD1562836_predicted"	"	-7.3249"	"0.01990238"
1393391_at	NA	"	-7.3245"	"0.01991705"
1369705_at	"Xtrp3"	"	7.3229"	"0.01998043"
1368943_at	"Rnase4"	"	-7.3221"	"0.02000902"
1383250_at	"Utp14a"	"	-7.3219"	"0.02001794"
1396872_at	NA	"	7.3199"	"0.02009668"
1395613_at	"Pard3"	"	7.3188"	"0.02013963"
1367759_at	"H1f0"	"	-7.3180"	"0.02017006"
1373872_at	NA	"	7.3177"	"0.02018217"
1378070_at	NA	"	-7.3163"	"0.02023580"
1373532_at	"Plekhf1"	"	7.3119"	"0.02040968"
1374874_at	"RGD1561042_predicted"	"	7.3107"	"0.02045518"
1387186_at	"Rab9"	"	7.3104"	"0.02046800"
1371277_at	"Cdx1"	"	7.3100"	"0.02048405"
1380797_at	"Asb13_predicted"	"	-7.3085"	"0.02054352"
1392097_at	NA	"	-7.3081"	"0.02056060"
1388762_at	"Iqgap1_predicted"	"	-7.3070"	"0.02060457"
1371427_at	"RGD1306911_predicted"	"	-7.3061"	"0.02063892"
1389545_at	"Tbl1xr1_predicted"	"	7.3036"	"0.02073767"
1383665_at	NA	"	7.3031"	"0.02076079"
1378146_at	NA	"	7.3027"	"0.02077601"
1373265_at	NA	"	7.3012"	"0.02083482"
1390675_at	NA	"	-7.3005"	"0.02086466"
1376272_s_at	"Zfpn1a2_predicted"	"	-7.2996"	"0.02089883"
1388770_at	"RGD1304890"	"	-7.2983"	"0.02095310"
1396254_at	"Ncoa2"	"	7.2979"	"0.02096831"
1379357_at	"RT1-Aw2"	"	7.2978"	"0.02097358"
1388497_at	"Them2_predicted"	"	7.2963"	"0.02103507"
1373791_at	"RGD1359127"	"	-7.2958"	"0.02105311"
1380387_at	"RGD1559697_predicted"	"	7.2945"	"0.02110611"
1383711_at	"Nmt1"	"	-7.2937"	"0.02114029"
1370130_at	"Rhoa"	"	-7.2917"	"0.02122285"
1390418_at	"Actr1b"	"	-7.2915"	"0.02122971"
1381082_at	"RGD1564357_predicted"	"	7.2898"	"0.02130188"
1383458_at	NA	"	7.2897"	"0.02130722"
1388829_at	"LOC361399"	"	-7.2859"	"0.02146120"
1372850_at	NA	"	-7.2821"	"0.02162265"
1380848_at	"LOC361990"	"	-7.2816"	"0.02164094"
1397685_at	NA	"	7.2815"	"0.02164841"

1367494_at	RGD1310899_predicted	-6.8039	0.00003986
1373930_at	LOC687681	6.803	0.00003988
1396029_at	Pde7b	6.8027	0.00003988
1389158_at	Rtel1	-6.8028	0.00003988
1376468_at	Dnd1	-6.8011	0.00003998
1388378_at	Eif3s8	-6.7982	0.00004018
1374480_at	Daam1_predicted	6.7971	0.00004021
1388102_at	Ltb4dh	6.7971	0.00004021
1378511_at	Wwox_predicted	6.7969	0.00004021
1371042_at	Map4k3	6.7962	0.00004025
1367980_at	Rabep1	6.7949	0.00004032
1376977_at	Ptger3	6.7939	0.00004039
1367470_at	Sar1a	-6.7936	0.00004039
1385497_x_at	Lnx1_predicted	6.79	0.00004065
1377069_at	NA	6.7868	0.00004088
1395306_at	RGD1565549_predicted	6.7817	0.00004124
1389969_at	Tomm40	-6.7817	0.00004124
1372150_at	Usp10	-6.7805	0.00004131
1383081_at	Csf2ra	-6.7792	0.00004139
1376069_at	Psmd11_predicted	-6.7764	0.0000416
1388607_at	Smurf2_predicted	6.7733	0.00004182
1388774_at	LOC680172	6.7695	0.00004211
1383302_at	Dnajb1_predicted	-6.7685	0.00004214
1392470_at	NIPBL	6.7687	0.00004214
1387900_at	Cdipt	-6.7622	0.00004264
1387160_at	Kcne3	-6.761	0.00004272
1376272_s_at	Zfpn1a2_predicted	-6.7597	0.0000428
1383922_a_at	Ard1_predicted	-6.7582	0.0000429
1373387_at	LOC688717	-6.756	0.00004306
1381925_x_at	Arid1b	6.7548	0.00004313
1399090_at	Dync1li1	-6.7536	0.00004317
1389380_at	LOC687266	-6.754	0.00004317
1388696_at	Ufd1l	-6.7536	0.00004317
1376754_at	Cars_predicted	-6.753	0.00004319
1370191_at	Azin1	-6.7527	0.0000432
1376267_at	NA	-6.751	0.00004329
1379247_at	RGD1564946_predicted	6.7512	0.00004329
1381260_at	RGD1560397_predicted	6.7503	0.00004333
1372216_at	NA	-6.7462	0.00004361
1389375_at	NA	6.7462	0.00004361
1372537_at	NA	6.7466	0.00004361
1395153_at	Cldn15_predicted	6.7449	0.00004369
1372395_at	RGD1565757_predicted	6.7434	0.00004377
1393043_at	Wdr50_predicted	-6.7435	0.00004377
1374669_at	LOC309016	6.7403	0.00004401
1391821_at	NA	6.7386	0.00004413
1372489_at	Slmap_predicted	6.738	0.00004417
1377700_at	NA	6.7344	0.00004445
1372545_at	NA	6.734	0.00004446
1384017_at	Lrrk1_predicted	6.7334	0.00004449

1379406_at	"RGD1308396_predicted"	" -7.2802"	"0.02170301"
1372742_at	"Ankmy2_predicted"	" 7.2771"	"0.02183280"
1375521_at	"Tceal8"	" -7.2744"	"0.02194790"
1389590_at	NA	" 7.2739"	"0.02196665"
1375432_at	"LOC679140"	" -7.2735"	"0.02198622"
1389638_at	"MGC94288"	" -7.2719"	"0.02205280"
1381822_x_at	"Trp53rk_predicted"	" -7.2707"	"0.02210679"
1374777_at	"LOC502894"	" -7.2704"	"0.02211751"
1369641_at	"Pafah1b2"	" -7.2696"	"0.02215129"
1373527_at	"LOC680987"	" -7.2695"	"0.02215651"
1370481_at	"Scnn1g"	" -7.2669"	"0.02226938"
1396076_at	NA	" 7.2663"	"0.02229341"
1389869_at	"LOC689397"	" -7.2656"	"0.02232461"
1380405_at	"Sept8_predicted"	" -7.2645"	"0.02237296"
1384794_at	NA	" -7.2641"	"0.02239057"
1367646_at	"Ctsb"	" 7.2632"	"0.02243020"
1388185_at	"Rb1"	" 7.2618"	"0.02248926"
1393106_x_at	"Gtf2f1"	" -7.2609"	"0.02252849"
1369635_at	"Sord"	" 7.2604"	"0.02254968"
1389627_at	"RGD1306209"	" -7.2570"	"0.02270071"
1386872_at	"Igf2r"	" 7.2561"	"0.02273985"
1389158_at	"Rtel1"	" -7.2548"	"0.02279692"
1373597_at	"Pop7_predicted"	" -7.2522"	"0.02291431"
1380301_at	NA	" -7.2519"	"0.02292640"
1391432_at	"Wdr75"	" -7.2484"	"0.02308230"
1386886_at	"Cd164"	" 7.2480"	"0.02310007"
1383671_at	"Adam9"	" -7.2467"	"0.02315865"
1381609_at	"Zfp335"	" -7.2446"	"0.02325694"
1376340_a_at	"Tssc4"	" -7.2429"	"0.02333400"
1379278_at	"RGD1311539_predicted"	" 7.2415"	"0.02339359"
1389680_at	"Ell2"	" 7.2396"	"0.02348397"
1383795_at	NA	" 7.2382"	"0.02354525"
1389998_at	"Nr2f2"	" -7.2353"	"0.02368036"
1375829_at	"RGD1308326_predicted"	" -7.2345"	"0.02371455"
1376186_at	"Waspip"	" 7.2337"	"0.02375277"
1369997_at	"Dvl1"	" -7.2329"	"0.02379074"
1390035_at	"LOC305076"	" -7.2321"	"0.02382758"
1373869_at	"Soat1"	" 7.2298"	"0.02393286"
1395655_at	NA	" 7.2297"	"0.02393814"
1367777_at	"Decr1"	" 7.2279"	"0.02402310"
1388766_at	"Mtx2"	" 7.2246"	"0.02417752"
1367740_at	"Ckb"	" -7.2244"	"0.02418956"
1374543_at	NA	" 7.2237"	"0.02422221"
1392662_at	"RGD1566242_predicted"	" -7.2226"	"0.02427186"
1387104_at	"Scnn1a"	" -7.2218"	"0.02431128"
1389166_at	"Cib2"	" -7.2212"	"0.02433953"
1376896_at	"RGD1309308_predicted"	" -7.2182"	"0.02448109"
1389480_at	"Rwdd4a"	" -7.2170"	"0.02453997"
1369586_at	"Mcpt8"	" 7.2157"	"0.02460067"
1376800_at	NA	" 7.2141"	"0.02467620"

1382679_at	LOC362703	-6.7293	0.00004482
1391433_at	Mte1	-6.7291	0.00004482
1383826_at	Rab40b_predicted	6.7283	0.00004486
1372054_at	NA	-6.7276	0.0000449
1391921_at	Rngtt_predicted	6.7265	0.00004497
1376941_at	Wbscr16_predicted	-6.7242	0.00004515
1385211_at	RGD1310951_predicted	6.7215	0.00004536
1383465_at	Fbxl14_predicted	-6.719	0.00004556
1370251_at	Avpi1	-6.7186	0.00004557
1382258_at	Rgnef_predicted	6.7171	0.00004568
1372116_at	Mrps2_predicted	-6.7167	0.00004569
1372106_at	Ehd4	-6.7146	0.00004585
1370530_a_at	Pld1	6.7129	0.00004598
1395578_at	NA	-6.7121	0.00004602
1375185_at	Ipo7_predicted	-6.7085	0.00004631
1372409_at	Mad2l1bp	-6.7084	0.00004631
1383660_at	NA	-6.706	0.0000465
1389093_at	Pak1	6.7048	0.00004659
1391432_at	Wdr75	-6.7043	0.00004661
1388514_at	Ppm1g	-6.7001	0.00004697
1389459_at	RGD1306613_predicted	-6.6943	0.00004747
1373405_at	NA	-6.6937	0.00004751
1368021_at	Adh1	6.6924	0.0000476
1372178_at	NA	6.692	0.00004761
1389057_at	Arv1_predicted	-6.6906	0.00004771
1389697_at	NA	6.6903	0.00004772
1380561_at	Iars2_predicted	6.6854	0.00004813
1384011_a_at	RGD1309729	-6.6853	0.00004813
1386662_at	NA	-6.6839	0.00004822
1371706_at	Sdccag3	-6.684	0.00004822
1375948_at	Alkbh5_predicted	-6.683	0.00004828
1376076_at	Mpp5_predicted	-6.6797	0.00004851
1383514_s_at	Narg1_predicted	-6.6799	0.00004851
1368295_at	Slco2b1	6.6798	0.00004851
1388520_at	Spag9_predicted	6.6787	0.00004856
1388410_at	Ugp2	6.679	0.00004856
1373994_at	RGD1565557_predicted	6.677	0.00004869
1393206_at	NA	6.6748	0.00004887
1393437_at	NA	6.6739	0.00004894
1367737_at	Fuca	6.6725	0.00004902
1370309_a_at	Hnrpab	-6.6728	0.00004902
1380437_at	NA	6.6713	0.00004911
1376723_a_at	RGD1310651_predicted	-6.671	0.00004911
1393910_at	RGD1309807	6.67	0.00004919
1389308_at	Dnajb11	-6.6694	0.00004922
1389182_at	RGD1311269_predicted	-6.6664	0.00004948
1374432_at	Alcam	6.6656	0.00004952
1384738_at	Nsf	-6.6647	0.00004959
1388655_at	Ssna1_predicted	-6.6644	0.0000496
1382596_a_at	RGD1311324	-6.6619	0.00004981

1385953_at	"Mllt3"	"	7.2132"	"0.02472072"
1398325_at	"Tspan3"	"	-7.2121"	"0.02477415"
1376047_at	"Papss2_predicted"	"	-7.2114"	"0.02480593"
1393282_at	"Fshprh1"	"	-7.2103"	"0.02485919"
1376081_at	"Lcmt2"	"	-7.2097"	"0.02489063"
1386718_at	"RGD1562954_predicted"	"	7.2088"	"0.02493320"
1398350_at	"Basp1"	"	7.2086"	"0.02494412"
AFFX_Rat_GAPD	"Gapdh"	"	7.2082"	"0.02496098"
1375602_at	"Rin2_predicted"	"	7.2071"	"0.02501439"
1398755_at	"Atp6v0c"	"	-7.2042"	"0.02515803"
1371793_at	"Ache"	"	-7.2001"	"0.02536125"
1383047_at	"Gas6"	"	-7.1989"	"0.02541727"
1388917_at	"Myo1d"	"	7.1968"	"0.02552436"
1384529_at	NA	"	7.1941"	"0.02565730"
1374656_at	"Exoc3"	"	7.1940"	"0.02566588"
1370330_at	"Sipa1l1"	"	7.1933"	"0.02570094"
1373838_at	"Fut4"	"	7.1909"	"0.02582006"
1376683_at	NA	"	7.1883"	"0.02594926"
1373038_at	"Mllt10"	"	7.1864"	"0.02604548"
1392938_s_at	"RGD1306959_predicted"	"	-7.1845"	"0.02614448"
1388378_at	"Eif3s8"	"	-7.1842"	"0.02615909"
1382006_at	"Snapap"	"	-7.1837"	"0.02618562"
1367565_a_at	"Fth1"	"	7.1835"	"0.02619423"
1373072_at	NA	"	-7.1822"	"0.02626448"
1389336_at	"Pop5_predicted"	"	-7.1748"	"0.02664347"
1372475_at	"Pink1_predicted"	"	7.1742"	"0.02667706"
1377628_at	NA	"	7.1719"	"0.02679429"
1393431_at	"LOC687575"	"	-7.1702"	"0.02688749"
1380941_at	NA	"	-7.1682"	"0.02698789"
1374332_at	"Pdcd11_predicted"	"	-7.1663"	"0.02708826"
1379337_at	"RGD1307883"	"	-7.1641"	"0.02720948"
1374702_at	NA	"	7.1640"	"0.02721138"
1378075_at	"Tm9sf4"	"	-7.1634"	"0.02724316"
1377735_at	"Snx24"	"	7.1573"	"0.02757436"
1395632_at	NA	"	-7.1553"	"0.02767836"
1371449_at	"Pin1_predicted"	"	-7.1529"	"0.02781282"
1392953_at	"LOC680115"	"	-7.1526"	"0.02782730"
1372546_at	"Mapkapk3"	"	-7.1509"	"0.02791879"
1398572_at	"LOC679672"	"	-7.1474"	"0.02810917"
1388397_at	"Ebna1bp2"	"	-7.1472"	"0.02812012"
1391804_at	NA	"	-7.1458"	"0.02819715"
1390576_at	"LOC684233"	"	-7.1425"	"0.02838038"
1389140_at	"Spcs2_predicted"	"	7.1413"	"0.02844703"
1397101_at	NA	"	7.1402"	"0.02851224"
1374379_at	NA	"	7.1393"	"0.02856079"
1371438_at	"Akna_predicted"	"	7.1389"	"0.02858451"
1379932_at	"Clcn4-2"	"	7.1388"	"0.02858837"
1391495_at	"RGD1311783_predicted"	"	-7.1375"	"0.02866207"
1384544_at	"Pon3"	"	7.1359"	"0.02875173"
1389197_at	"RGD1306248"	"	-7.1344"	"0.02883576"

1371437_at	Sec13l1	-6.6601	0.00004996
1376143_at	NA	-6.6585	0.00005006
1388916_at	RGD1564074_predicted	6.6588	0.00005006
1372504_at	LOC692000	-6.6573	0.00005016
1382444_at	NA	6.6567	0.00005018
1383549_at	RGD1310794	-6.6566	0.00005018
1392502_at	LOC682398	-6.6519	0.00005059
1382166_at	Srprb	-6.6518	0.00005059
1373269_at	NA	-6.6511	0.00005063
1368248_at	Cds1	6.6494	0.00005077
1395520_at	Rbm28_predicted	-6.649	0.00005079
1396139_at	Arhgap26_predicted	6.6487	0.0000508
1388708_at	LOC688813	-6.6484	0.0000508
1374947_at	Bcar3_predicted	-6.6477	0.00005084
1395852_at	Abca3	6.6468	0.00005088
1371806_at	Dgcr8_predicted	-6.6468	0.00005088
1372973_at	Lss	6.6446	0.00005108
1371675_at	NA	6.6427	0.00005123
1372140_at	RGD1310326	6.64	0.00005148
1389516_at	LOC680620	6.6386	0.0000516
1374058_at	Ascl3	-6.638	0.00005163
1375460_at	RGD1309441_predicted	-6.6368	0.00005172
1370503_s_at	Epb4.1l3	6.635	0.00005188
1389927_at	NA	6.6327	0.00005209
1380681_at	LOC685384	6.6321	0.00005212
1383108_at	Taf13_predicted	-6.6283	0.00005243
1381822_x_at	Trp53rk_predicted	-6.6288	0.00005243
1388798_at	Ube2e2	6.6285	0.00005243
1369013_a_at	Mrpl17	-6.6274	0.00005249
1378062_at	Arhgef12	-6.6243	0.00005278
1376145_at	Eif2b5	-6.6233	0.00005286
1376040_at	Sipa1l2	6.6225	0.00005292
1372081_at	NA	6.6206	0.00005309
1372612_at	Dynll2	-6.6164	0.0000535
1374836_at	Rnu3ip2_predicted	-6.6142	0.0000537
1382271_at	Rps6ka5_predicted	6.6118	0.00005393
1369313_at	Fhl2	-6.6113	0.00005394
1394109_at	Thbs1	-6.6112	0.00005394
1372011_at	NA	6.6107	0.00005396
1381814_at	RGD1566016_predicted	6.6103	0.00005398
1381404_at	Mbd1	-6.6094	0.00005405
1389580_at	Smarca3_predicted	6.6063	0.00005434
1377738_a_at	Cyhr1	6.6051	0.00005439
1373172_at	NA	6.6051	0.00005439
1383573_at	Sdccag33_predicted	6.6052	0.00005439
1398969_at	NA	-6.6043	0.00005445
1375412_at	NA	6.5999	0.0000549
1381687_at	NA	-6.5936	0.00005555
1368235_at	Clk3	-6.5925	0.00005561
1384621_at	NA	6.5926	0.00005561

1371848_at	"LOC687711"	" -7.1329"	"0.02891954"
1378672_at	NA	" 7.1327"	"0.02893193"
1374524_at	"Scly"	" 7.1326"	"0.02894061"
1378346_at	"Emilin2_predicted"	" 7.1320"	"0.02897054"
1369686_at	"Dclk1"	" 7.1300"	"0.02908417"
1374289_at	"Rpo1-1"	" -7.1288"	"0.02915276"
1373345_at	"Amigo2"	" 7.1287"	"0.02915920"
1383716_at	NA	" -7.1278"	"0.02921289"
1377180_at	NA	" 7.1249"	"0.02937682"
1396816_at	NA	" -7.1242"	"0.02941858"
1376100_at	"Tubb6"	" -7.1240"	"0.02943253"
1395751_at	"LOC680927"	" -7.1217"	"0.02956543"
1383357_a_at	NA	" 7.1210"	"0.02960657"
1372898_at	"RGD1566204_predicted"	" -7.1192"	"0.02971074"
1388600_at	NA	" -7.1182"	"0.02976964"
1392747_at	"Fbxo30"	" -7.1160"	"0.02989316"
1391023_at	NA	" 7.1087"	"0.03032595"
1390144_at	"RGD1359108"	" 7.1074"	"0.03040326"
1382794_at	"Galnt10"	" 7.1066"	"0.03045354"
1380027_at	NA	" -7.1052"	"0.03053829"
1389203_at	"LOC690898"	" -7.1044"	"0.03058630"
1383228_at	NA	" 7.1043"	"0.03058778"
1377806_at	"Vezf1_predicted"	" -7.1008"	"0.03079952"
1374142_at	"RGD1311589_predicted"	" 7.1007"	"0.03080478"
1392969_at	NA	" -7.1000"	"0.03085037"
1389556_at	"Kifap3_predicted"	" 7.0990"	"0.03090864"
1392172_at	"Ccl9"	" -7.0983"	"0.03095018"
1390478_at	"Orc4"	" 7.0978"	"0.03098555"
1387841_at	"Exoc5"	" -7.0967"	"0.03104855"
1392301_at	"Sh3tc1_predicted"	" 7.0943"	"0.03119672"
1388709_at	"LOC362703"	" -7.0923"	"0.03132026"
1374638_at	"Pex13_predicted"	" 7.0903"	"0.03144480"
1388952_at	"RGD1307935"	" 7.0883"	"0.03156771"
1379384_at	"Sp1"	" -7.0862"	"0.03169643"
1393255_at	NA	" 7.0834"	"0.03187136"
1389262_at	NA	" -7.0831"	"0.03189116"
1369636_at	"Sord"	" 7.0826"	"0.03192124"
1378032_at	"Nfkbiz_predicted"	" -7.0823"	"0.03194117"
1374548_at	"Aff4_predicted"	" -7.0819"	"0.03196863"
1370144_at	"Gtpbp4"	" -7.0818"	"0.03197384"
1379415_at	NA	" -7.0807"	"0.03204090"
1370482_at	"Scnn1b"	" -7.0778"	"0.03222725"
1384816_at	"Cxadr"	" 7.0773"	"0.03225492"
1372316_at	"RGD1311939_predicted"	" 7.0741"	"0.03246188"
1388587_at	"Ier3"	" -7.0721"	"0.03258733"
1393154_at	NA	" 7.0718"	"0.03260629"
1388450_at	"Ap1gbp1"	" -7.0701"	"0.03271623"
1388571_at	"Syngr2"	" -7.0692"	"0.03277406"
1387342_at	"Gng5"	" -7.0662"	"0.03296717"
1381816_at	"RGD1309804"	" 7.0654"	"0.03302055"

1377872_at	RGD1306936_predicted	-6.5922	0.00005562
1383632_at	NA	-6.5915	0.00005567
1370974_at	Vps54	6.5901	0.00005579
1371312_at	Chchd2	-6.5873	0.00005607

1372544_at	NA	" 7.0641"	"0.03310324"
1368818_at	"Psme4"	" 7.0637"	"0.03313012"
1382500_at	NA	" -7.0597"	"0.03339328"
1369743_a_at	"P2rx4"	" -7.0592"	"0.03342494"
1388076_at	"Serbp1"	" -7.0589"	"0.03344860"
1379257_at	"Epb4.1l4a_predicted"	" 7.0584"	"0.03348292"
1372829_at	"Rbm8_predicted"	" -7.0573"	"0.03355160"
1390384_at	"RGD1566119_predicted"	" -7.0558"	"0.03365019"
1388916_at	"RGD1564074_predicted"	" 7.0544"	"0.03374330"
1380297_at	NA	" 7.0530"	"0.03383954"
1369868_at	"Iag2"	" -7.0513"	"0.03395368"
1388154_at	"E2f5"	" -7.0505"	"0.03400206"
1380228_at	"RGD1306880_predicted"	" 7.0493"	"0.03408721"
1377676_at	"Nucks"	" -7.0476"	"0.03419700"
1393119_at	NA	" -7.0472"	"0.03422706"
1375651_at	NA	" 7.0428"	"0.03452740"
1367466_at	"Prpf8"	" -7.0405"	"0.03467929"
1367476_at	"Srp14_predicted"	" -7.0401"	"0.03470731"
1374437_at	"Nars"	" -7.0392"	"0.03477414"
1371697_at	"Pnpla2_predicted"	" 7.0379"	"0.03485833"
1398897_at	"Ube2v1_predicted"	" -7.0323"	"0.03524673"
1388379_at	"Ptpn11"	" -7.0309"	"0.03534207"
1378778_a_at	NA	" -7.0303"	"0.03538491"
1371388_at	"Pdhb"	" 7.0274"	"0.03559031"
1370776_a_at	"Kcnk6"	" -7.0228"	"0.03591321"
1374442_at	"Sfrs9"	" -7.0225"	"0.03593910"
1372318_at	NA	" 7.0174"	"0.03629895"
1371882_a_at	"Sh2bpsm1"	" -7.0159"	"0.03640951"
1389927_at	NA	" 7.0145"	"0.03650879"
1390952_at	NA	" -7.0100"	"0.03683688"
1384967_at	"Sart2_predicted"	" 7.0063"	"0.03710734"
1376665_at	NA	" 7.0060"	"0.03712997"
1389830_at	"Ubtf"	" -7.0047"	"0.03722055"
1374178_at	NA	" 6.9984"	"0.03768706"
1397953_at	"LOC687696"	" -6.9970"	"0.03779102"
1368073_at	"Irf1"	" 6.9963"	"0.03784331"
1372795_at	"Txnl4b"	" -6.9954"	"0.03791573"
1388381_at	"Eif3s4"	" -6.9949"	"0.03794778"
1374251_at	NA	" 6.9946"	"0.03797713"
1376296_at	"Pik3r1"	" 6.9935"	"0.03805531"
1390394_at	NA	" -6.9931"	"0.03808345"
1376291_at	NA	" -6.9909"	"0.03825568"
1392527_at	"RGD1560220_predicted"	" 6.9899"	"0.03832580"
1389306_at	"Cd151"	" -6.9899"	"0.03832715"
1399014_at	"Vps4a"	" -6.9897"	"0.03834687"
1383166_at	"Ncoa1_predicted"	" 6.9884"	"0.03844312"
1391017_at	"Farp1_predicted"	" 6.9882"	"0.03846068"
1398786_at	"Psmb2"	" -6.9873"	"0.03852365"
1388392_at	"Tax1bp3"	" -6.9870"	"0.03854608"
1371735_at	NA	" -6.9866"	"0.03858154"

1381470_at	NA	" 6.9849"	"0.03870688"
1367750_at	"Prpsap1"	" -6.9837"	"0.03880055"
1371214_at	"Itga7"	" 6.9835"	"0.03881655"
1371967_at	"Mrpl16"	" -6.9808"	"0.03902758"
1388897_at	"Wdr5"	" -6.9755"	"0.03943420"
1388449_at	"Eef1b2_predicted"	" -6.9742"	"0.03953728"
1385553_at	"LOC689116"	" 6.9740"	"0.03955328"
1384791_at	"B3gnt1_predicted"	" -6.9738"	"0.03956635"
1391643_at	NA	" -6.9737"	"0.03958110"
1372417_at	"Sertad1"	" -6.9723"	"0.03968480"
1399024_at	"Scyl1"	" -6.9722"	"0.03969466"
1398801_at	"Cdk105"	" -6.9709"	"0.03979457"
1373552_at	"Las1l_predicted"	" -6.9689"	"0.03995445"
1391230_at	"Sort1"	" -6.9686"	"0.03997609"
1395213_at	"RGD1562620_predicted"	" -6.9682"	"0.04001237"
1384399_at	"Ankrd50_predicted"	" 6.9674"	"0.04007690"
1390803_at	"Htatsf1_predicted"	" -6.9670"	"0.04010875"
1386078_at	"Slc35a1_predicted"	" 6.9639"	"0.04035811"
1383176_at	"RGD1306959_predicted"	" -6.9626"	"0.04046059"
1379704_at	"Zfp143"	" -6.9625"	"0.04046938"
1383557_at	NA	" 6.9614"	"0.04055070"
1391476_at	NA	" -6.9589"	"0.04075379"
1372721_at	"Zfpn1a2_predicted"	" -6.9573"	"0.04088483"
1374265_at	NA	" 6.9560"	"0.04099418"
1383969_at	"RGD1561832_predicted"	" 6.9517"	"0.04134534"
1398792_at	"Psmc1"	" -6.9514"	"0.04136371"
1383148_at	NA	" 6.9508"	"0.04141362"
1377235_a_at	"Ppp2r5b"	" 6.9496"	"0.04151551"
1383661_at	"Hs3st3b1_predicted"	" 6.9473"	"0.04170520"
1388334_at	"RGD1563123_predicted"	" 6.9471"	"0.04172306"
1376660_at	NA	" 6.9459"	"0.04182001"
1389065_at	"Rbm34"	" -6.9452"	"0.04187936"
1380544_at	NA	" -6.9451"	"0.04188414"
1383299_at	NA	" 6.9437"	"0.04200486"
1390300_at	NA	" 6.9437"	"0.04200504"
1395730_at	"RGD1559871_predicted"	" -6.9430"	"0.04206413"
1389381_at	"Sqstm1"	" -6.9416"	"0.04217622"
1371324_at	"LOC680891"	" -6.9396"	"0.04234959"
1373172_at	NA	" 6.9387"	"0.04242666"
1374156_at	"Mpp5_predicted"	" 6.9384"	"0.04245117"
1393148_at	"Gpr137b_predicted"	" -6.9367"	"0.04259433"
1381612_at	NA	" 6.9349"	"0.04274205"
1398892_at	"Npc2"	" -6.9326"	"0.04293866"
1395721_at	"Papss2_predicted"	" -6.9322"	"0.04297380"
1387911_at	"Rabggtb"	" -6.9321"	"0.04298400"
1368465_at	"Accn1"	" 6.9319"	"0.04300155"
1367711_at	"Psmc2"	" -6.9311"	"0.04307096"
1375107_at	NA	" 6.9308"	"0.04309418"
1377758_at	"Hsd17b13"	" 6.9301"	"0.04315385"
1371617_at	"Psmd13_predicted"	" -6.9293"	"0.04321884"

1373965_at	"RGD1310931_predicted"	" -6.9280"	"0.04333266"
1368268_at	"Tdg"	" -6.9263"	"0.04348395"
1399059_at	"Ezh1_predicted"	" -6.9258"	"0.04352295"
1376747_at	NA	" 6.9246"	"0.04363131"
1381905_at	"Herpud1"	" 6.9232"	"0.04375095"
1373269_at	NA	" -6.9229"	"0.04377929"
1383805_at	"RGD1561817_predicted"	" 6.9224"	"0.04382375"
1372602_at	"RGD1311800"	" 6.9214"	"0.04390507"
1368141_at	"Cnbp1"	" -6.9214"	"0.04390928"
1374662_at	"Slc35a1_predicted"	" 6.9209"	"0.04395470"
1384296_at	NA	" 6.9208"	"0.04396091"
1367807_at	"Plod1"	" 6.9200"	"0.04402808"
1371422_at	"Morf4l2"	" -6.9183"	"0.04417486"
1367661_at	"S100a6"	" -6.9183"	"0.04418125"
1380250_at	"Sned1"	" -6.9179"	"0.04421219"
1391460_at	NA	" 6.9174"	"0.04425470"
1392897_at	"LOC360990"	" 6.9166"	"0.04432386"
1384046_at	"Mtfr1_predicted"	" -6.9163"	"0.04435576"
1370888_at	"Cox5a"	" 6.9151"	"0.04446197"
1372824_at	"Plekhf2_predicted"	" -6.9145"	"0.04451100"
1374266_at	"Pcdh1_predicted"	" -6.9132"	"0.04462570"
1389577_at	"Cirh1a"	" -6.9113"	"0.04479694"
1373974_at	"Osbp_predicted"	" -6.9106"	"0.04486015"
1371822_at	"Polr3d"	" -6.9104"	"0.04487492"
1372954_at	NA	" 6.9103"	"0.04488606"
1377885_at	"LOC685203"	" -6.9102"	"0.04490002"
1387299_at	"Miz1"	" -6.9087"	"0.04503102"
1376639_at	"Rnf126"	" -6.9037"	"0.04547764"
1382765_at	"LOC691918"	" -6.9024"	"0.04559779"
1388754_at	"Nudt7_predicted"	" -6.8968"	"0.04611033"
1392960_at	"RGD1309906"	" -6.8965"	"0.04613399"
1393228_at	"Gloxd1"	" -6.8962"	"0.04616183"
1377387_a_at	NA	" -6.8955"	"0.04623212"
1376990_at	"RGD1309307_predicted"	" 6.8954"	"0.04623678"
1376734_at	NA	" -6.8932"	"0.04644610"
1387948_at	"Ick"	" 6.8915"	"0.04659485"
1374222_at	"Slc22a18"	" 6.8908"	"0.04666196"
1385251_at	"LOC500638"	" 6.8898"	"0.04676129"
1376325_at	"Nfrkb_predicted"	" -6.8859"	"0.04712274"
1375686_at	"Ppil3"	" -6.8850"	"0.04720343"
1374842_at	"Cebpz_predicted"	" -6.8838"	"0.04731568"
1373213_at	"Snap29"	" -6.8812"	"0.04756424"
1382328_at	"Gimap1"	" -6.8806"	"0.04762175"
1379659_at	"Bmp2k"	" -6.8804"	"0.04764259"
1374385_at	"RGD1561319_predicted"	" -6.8793"	"0.04774480"
1389279_at	NA	" -6.8784"	"0.04783596"
1372567_at	"R3hcc1_predicted"	" -6.8778"	"0.04788958"
1398853_at	"Psmb3"	" -6.8753"	"0.04812465"
1382012_at	"Rtf1_predicted"	" -6.8752"	"0.04813615"
1368521_at	"Napsa"	" 6.8734"	"0.04830688"

1375970_at	NA	" 6.8728"	"0.04836908"		
1398758_at	"Arf4"	" -6.8719"	"0.04846078"		
1390010_at	"Ncoa1_predicted"	" 6.8716"	"0.04848330"		
1383108_at	"Taf13_predicted"	" -6.8700"	"0.04863800"		
1382526_at	"RGD1309308_predicted"	" -6.8691"	"0.04872998"		
1383352_at	NA	" -6.8687"	"0.04876467"		
1399011_at	"Cops6_predicted"	" -6.8679"	"0.04884629"		
1376062_at	"Sdc1"	" -6.8674"	"0.04889538"		
1374185_at	"Txndc11_predicted"	" -6.8669"	"0.04894229"		
1377322_at	NA	" -6.8667"	"0.04896563"		
1373427_at	"Rragd_predicted"	" 6.8620"	"0.04941981"		
1367513_at	"Tm9sf4"	" -6.8587"	"0.04974903"		
1374737_at	"LOC690308"	" -6.8577"	"0.04985186"		
1373332_at	"Csnk1d"	" -6.8575"	"0.04987130"		
1369156_at	"Frk"	" 6.8571"	"0.04990733"		

Table S2: A) Genes with Gene Ontology Biological Processing term associated with DNA damage and repair in the AOM versus saline contrast in the proximal colon.

Probe set ID	Go Biological Processing Term	Gene Name	Gene Symbol	Pvalue	Fold change^
1368311_at	DNA repair	DNA methyltransferase	Mgmt	3.83E-05	-1.39476
1372143_at	DNA double-strand break processing	enzyme E2 variant 2	Ube2v2	6.04E-05	1.27951
1389090_at	DNA synthesis during DNA repair	interacting protein 1	Wrnip1	0.000285302	1.21353
1389011_at	DNA repair	MMS21 homolog (S.	Nsmce2	0.000325247	-1.44944
1388135_at	nucleotide-excision repair, DNA damage removal	replication protein A2	Rpa2	0.000537544	1.45881
1379264_at	DNA repair	zinc and ring finger 1	Znrf1	0.000722048	-1.15791
1368947_at	G2/M transition of mitotic cell cycle	damage-inducible 45	Gadd45a	0.000749954	1.52946
1373094_at	nucleotide-excision repair, DNA damage removal	factor II H, polypeptide	Gtf2h1	0.000787824	1.14086
1395488_at	base-excision repair	coli)	Msh3	0.00117105	-1.38696
1375956_at	nucleotide-excision repair, DNA damage removal	menage a trois 1	Mnat1	0.00125185	-1.25877
1393596_at	DNA repair	thalassemia/mental	Atrx	0.00164429	-1.34289
1368083_at	nucleotide-excision repair, DNA damage removal	cyclin H	Ccnh	0.00208666	1.22118

Table S2:B) Genes with Gene Ontology Biological Processing term associated with DNA damage and repair in the AOM versus saline contrast in the distal colon.

Probe set ID	Go Biological Processing Term	Gene Name	Gene Symbol	Pvalue	Fold change^
1395488_at	base-excision repair	mutS homolog 3 (E. coli)	Msh3	5.27E-11	-2.13062
1393963_at	base-excision repair	like 1 (E.coli)	Nthl1	0.00183121	-1.1797
1367676_at	base-excision repair, DNA ligation	2	Hmgb2	0.00896885	-1.19742
1387026_at	cell cycle checkpoint	of chromosomes 1A	Smc1a	5.08E-06	-1.1472
1374011_at	cell cycle checkpoint	excision repair cross-complementing rodent repair deficiency, complementation gr	Ercc3	0.000222108	1.11635
1371648_at	cell cycle checkpoint	binding protein 1	Ddb1	0.000910499	1.14037
1398881_at	cell cycle checkpoint	binding protein 1	Ddb1	0.00516406	1.11167
1399162_a_at	cell cycle checkpoint	binding protein 1	Ddb1	0.00546849	1.10902
1387977_at	cell cycle checkpoint_damaged DNA binding	nibrin	Nbn	0.000113082	1.27287
1372143_at	DNA double-strand break processing	enzyme E2 variant 2	Ube2v2	7.79E-12	1.60889
1397039_at	DNA metabolic process	complementing rodent repair deficiency,	Ercc4l1	8.90E-05	1.20114
1368311_at	DNA repair	DNA methyltransferase	Mgmt	1.05E-10	-1.74489
1379264_at	DNA repair	zinc and ring finger 1	Znrf1	1.68E-10	-1.35981
1389011_at	DNA repair	MMS21 homolog (S.	Nsmce2	8.80E-10	-1.95721
1386910_a_at	DNA repair	endonuclease 1	Apex1	9.24E-10	1.63157
1377700_at	DNA repair	thalassemia/mental	Atrx	4.41E-09	-1.52823

1374245_at	DNA repair	joining factor 1	Nhej1	8.79E-08	-1.58677
1386683_at	DNA repair	thalassemia/mental	Atrx	4.83E-07	-1.72997
1372548_at	DNA repair	(photolyase-like)	Cry2	7.34E-07	-1.57269
1376687_at	DNA repair	peptdiase 1	Usp1	4.24E-06	1.35579
1373280_at	DNA repair	RuvB-like protein 2	Ruvbl2	4.40E-06	1.20269
1392640_at	DNA repair	(photolyase-like)	Cry1	5.38E-06	1.40332
1372393_at	DNA repair	F-box only protein 18	Fbxo18	1.06E-05	-1.15062
1383184_at	DNA repair	zinc and ring finger 1	Znrf1	2.79E-05	-1.26121
1385616_a_at	DNA repair	function 1 homolog A	Asf1a	4.00E-05	1.17955
1373538_at	DNA repair	peptdiase 1	Usp1	6.79E-05	1.56608
1369446_at	DNA repair	(photolyase-like)	Cry2	0.000157113	-1.25513
1393367_at	DNA repair	phosphatase	Pnkp	0.00024632	1.13521
1374788_at	DNA repair	protein 53 binding	Trp53bp1	0.000304299	-1.12873
1393798_at	DNA repair	thalassemia/mental	Atrx	0.000508777	-1.74408
1377648_at	DNA repair	similarity 120B	Fam120b	0.000655171	-1.14936
1368410_at	DNA repair	glycosylase	Mpg	0.00091798	1.16907
1378640_at	DNA repair	PHD and RING finger	Uhrf1	0.0017196	1.79995
1368787_at	DNA repair	mutY homolog (E. coli)	Mutyh	0.00180015	-1.17966
1375793_at	DNA repair	mixed-lineage leukemia	Mll1	0.00186981	-1.18358
1393596_at	DNA repair	thalassemia/mental	Atrx	0.0024896	-1.26816
1385006_at	DNA repair	thalassemia/mental	Atrx	0.00521942	-1.1863
1398494_at	DNA repair	cerevisiae)	Rev1	0.00562161	1.14881
1377137_at	DNA repair	phosphodiesterase 1	Tdp1	0.00892211	-1.14795
1382752_at	DNA repair	protein 53 binding	Trp53bp1	0.0108457	-1.1095
1374210_at	DNA repair	homolog (S. cerevisiae)	Nsmce1	0.012704	-1.08763
1393654_at	DNA repair	like 1 (E. coli)	Neil1	0.0138863	1.14435
1390384_at	DNA replication checkpoint	member X	H2afx	3.08E-08	1.52198
1369010_at	DNA replication checkpoint	homolog (S. pombe)	Chek2	0.000196838	-1.40682
1388379_at	DNA replication checkpoint	phosphatase, non-	Ptpn11	0.000386222	1.11453
1377967_at	DNA replication checkpoint	DNA replication factor 1	Cdt1	0.000891174	1.46323
1387062_a_at	DNA replication checkpoint	homolog (S. pombe)	Chek1	0.000973697	1.64014
1378847_x_at	DNA replication checkpoint	member X	H2afx	0.00177604	1.28489
1387668_at	DNA replication checkpoint	phosphatase, non-	Ptpn11	0.00382196	1.14079
1367697_at	DNA replication checkpoint	protein kinase 14	Mapk14	0.00507796	-1.15285
1385090_at	DNA replication checkpoint	pombe)	Rad17	0.0135581	1.10802
1389090_at	DNA synthesis during DNA repair	interacting protein 1	Wrnip1	0.000195022	1.18388
1372366_at	double-strand break repair	protein, homolog	Htatip	2.10E-07	1.27244
1367455_at	double-strand break repair	protein	Vcp	5.60E-06	1.25116
1370931_at	double-strand break repair	complementing	Xrcc5	1.60E-05	-1.18161
1370537_at	double-strand break repair	complementing	Xrcc6	0.00540641	1.16508
1367500_at	double-strand break repair via homologous recombination	malformation	Shfm1	1.19E-05	1.13522

1393405_at	double-strand break repair via homologous recombination	complementing	Xrcc2	2.11E-05	1.4922
1399143_at	double-strand break repair via homologous recombination	enzyme E2N	Ube2n	9.09E-05	1.42579
1369617_at	double-strand break repair via homologous recombination	enzyme E2N	Ube2n	0.00103024	1.3209
1368947_at	G2/M transition of mitotic cell cycle	damage-inducible 45	Gadd45a	4.12E-06	1.70719
1374304_at	double-strand break by non-homologous end joining	complementing	Xrcc4	1.54E-13	-1.94045
1367488_at	mismatch repair	17	Ankrd17	3.19E-08	-1.22028
1399008_at	mismatch repair	17	Ankrd17	3.23E-05	-1.18747
1384523_at	mismatch repair	increased 1 (S.	Pms1	0.00155457	-1.23014
1384119_at	mismatch repair	coli)	Mlh3	0.00988104	-1.10683
1397245_at	nucleotide-excision repair	cerevisiae)	Rad23a	8.85E-05	1.22301
1368083_at	nucleotide-excision repair, DNA damage removal	cyclin H	Ccnh	2.24E-10	1.58756
1375956_at	nucleotide-excision repair, DNA damage removal	menage a trois 1	Mnat1	2.31E-10	-1.6535
1384029_at	nucleotide-excision repair, DNA damage removal	pigmentosum,	Xpa	4.09E-07	-1.48562
1388135_at	nucleotide-excision repair, DNA damage removal	replication protein A2	Rpa2	3.03E-06	1.59884
1373094_at	nucleotide-excision repair, DNA damage removal	factor II H, polypeptide	Gtf2h1	1.63E-05	1.16461
1399032_at	nucleotide-excision repair, DNA damage removal	complementing rodent repair deficiency,	Ercc1	0.000223246	1.18331
1372415_at	nucleotide-excision repair, DNA damage removal	factor IIH, polypeptide 3	Gtf2h3	0.00268885	1.08299
1394686_at	nucleotide-excision repair, DNA damage removal	complementing rodent repair deficiency,	Ercc4	0.00334532	1.19607
1385803_at	nucleotide-excision repair, DNA damage removal	factor II H, polypeptide	Gtf2h2	0.00436729	1.14548
1383346_at	nucleotide-excision repair, DNA damage removal	complementing rodent repair deficiency,	Ercc4	0.00494953	1.10233
1382030_at	nucleotide-excision repair, DNA damage removal	factor II H, polypeptide	Gtf2h2	0.00549515	1.17989
1397557_at	nucleotide-excision repair, DNA damage removal	factor II H, polypeptide	Gtf2h1	0.00706177	1.20328
1388550_at	nucleotide-excision repair, DNA damage removal	cerevisiae)	Rad23b	0.00983991	1.10832
1383953_at	nucleotide-excision repair, DNA damage removal	complementing rodent repair deficiency,	Ercc5	0.0147419	-1.19381
1372828_at	protein repair	reductase B2	Msrb2	0.00382852	-1.15413
1368025_at	response to hypoxia	transcript 4	Ddit4	0.00889726	1.36865
1383597_at	telomere maintenance	1C, PSO2 homolog (S.	Dclre1c	0.00558418	-1.14432

^positive fold change means up with AOM.

Table S3: Summary of genes^ validated by real time PCR for microarray differential expression.

Gene Symbol	Normalised to Ribosomal Protein	Proximal vs distal		AOM vs saline, all		AOM vs saline, proximal		AOM vs saline, distal	
	Test used	Expressn ratio	p-value	Expressn ratio	p-value	Expressn ratio	p-value	Expressn ratio	p-value
UDP-GlcNAc	t test on log of ratio	0.018 (0.01-0.03)	<0.00005	0.493 (0.65-1.34)	0.69	0.914 (0.41-2.01)	0.80	0.950 (0.78-1.16)	0.57
	Wilcoxon signed rank		0.0001		0.55		0.96		0.72
SLC34A2	t test on log of ratio	0.043 (0.03-0.06)	<0.00005	0.780 (0.55-2.64)	0.15	0.530 (0.29-0.98)	0.04	1.145 (0.92-1.43)	0.20
	Wilcoxon signed rank		0.0001		0.76		0.44		0.24
GlcNAc6ST-1	t test on log of ratio	0.0086 (0.006-0.01)	<0.00005	1.014 (0.72-1.43)	0.93	1.335 (0.67-2.64)	0.36	0.770 (0.65-0.91)	0.007
	Wilcoxon signed rank		0.0001		0.74		0.24		0.03
PRDX6	t test on log of ratio	2.743 (2.26-3.33)	<0.00005	1.180 (0.58-1.47)	0.13	1.295 (0.88-1.90)	0.16	1.07 (0.81-1.43)	0.56
	Wilcoxon signed rank		0.0001		0.29		0.20		0.88
HOXD10	t test on log of ratio	0.003 (0.002-0.005)	<0.00005	0.417 (0.29-0.61)	0.0001	0.375 (0.169-0.833)	0.02	0.464 (0.38-0.57)	<0.00005
	Wilcoxon signed rank		0.0001		0.0015		0.07		0.005
PRKACB	t test on log of ratio	0.042 (0.04-0.05)	<0.00005	0.638 (0.54-0.75)	<0.00005	0.775 (0.59-1.02)	0.06	0.525 (0.45-0.61)	<0.00005
	Wilcoxon signed rank		0.0001		0.002		0.15		0.005
CYP4F1	t test on log of ratio	77.822 (66.9-90.4)	<0.00005	0.844 (0.67-1.06)	0.14	0.891 (0.70-1.13)	0.31	0.800 (0.51-1.25)	0.29
	Wilcoxon signed rank		0.0001		0.39		0.44		0.57
CASC4	t test on log of ratio	1.782 (1.45-2.18)	<0.00005	0.789 (0.62-1.01)	0.06	0.919 (0.62-1.36)	0.64	0.677 (0.48-0.95)	0.03
UDP-GlcNAc	Wilcoxon signed rank		0.0001		0.18		0.92		0.06

Gene Symbol	Normalised to 18s	Proximal vs distal		AOM vs saline, all		AOM vs saline, proximal		AOM vs saline, distal	
	Test used	Expressn ratio	p-value	Expressn ratio	p-value	Expressn ratio	p-value	Expressn ratio	p-value
UDP-GlcNAc	t test on log of ratio	0.018 (0.01-0.03)	<0.00005	0.493 (0.65-1.34)	0.69	1.049 (0.0.47-2.33)	0.89	1.042 (0.46-2.34)	0.91
	Wilcoxon signed rank		0.0001		0.55		0.57		0.96
SLC34A2	t test on log of ratio	0.043 (0.03-0.06)	<0.00005	0.780 (0.55-2.64)	0.15	0.688 (0.30-1.55)	0.33	1.234 (0.70-2.17)	0.42
	Wilcoxon signed rank		0.0001		0.76		0.76		0.88
GlcNAc6ST-1	t test on log of ratio	0.0086 (0.006-0.01)	<0.00005	1.014 (0.72-1.43)	0.93	1.496 (0.69-3.23)	0.27	0.857 (0.44-1.66)	0.61
	Wilcoxon signed rank		0.0001		0.74		0.24		0.96
PRDX6	t test on log of ratio	2.743 (2.26-3.33)	<0.00005	1.180 (0.58-1.47)	0.13	1.647 (1.04-2.61)	0.04	1.184 (0.60-2.35)	0.59
	Wilcoxon signed rank		0.0001		0.29		0.02		0.51
HOXD10	t test on log of ratio	0.003 (0.002-0.005)	<0.00005	0.417 (0.29-0.61)	0.0001	0.457 (0.13-1.61)	0.19	0.506 (0.23-1.10)	0.08
	Wilcoxon signed rank		0.0001		0.0015		0.96		0.96
PRKACB	t test on log of ratio	0.042 (0.04-0.05)	<0.00005	0.638 (0.54-0.75)	<0.00005	0.963 (0.60-1.54)	0.86	0.589 (0.30-1.15)	0.11
	Wilcoxon signed rank		0.0001		0.002		0.88		0.65
CYP4F1	t test on log of ratio	77.822 (66.9-90.4)	<0.00005	0.844 (0.67-1.06)	0.14	1.095 (0.60-1.99)	0.74	0.801 (0.34-1.88)	0.57
	Wilcoxon signed rank		0.0001		0.39		0.96		0.72
CASC4	t test on log of ratio	1.782 (1.45-2.18)	<0.00005	0.789 (0.62-1.01)	0.06	1.154 (0.62-2.15)	0.61	0.654 (0.30-1.41)	0.24
	Wilcoxon signed rank		0.0001		0.18		0.65		0.96

^**Gene key:** UDP-GlcNAc: betaGal beta-1,3-N-acetylglucosaminyltransferase 7, SLC34A2: solute carrier family 34 (sodium phosphate), member 2; GlcNAc6ST-1: similar to N-acetylglucosamine 6-O-sulfotransferase (predicted), PRDX6: peroxiredoxin 6, HOXD10: homeo box D10 (predicted), PRKACB:protein kinase, cAMP dependent, catalytic, beta; CYP4F1:cytochrome P450, family 4, subfamily f, polypeptide 1, CASC4:cancer susceptibility candidate 4 (predicted).

Table S4: Genes differentially expressed in the p53 signalling pathway

Symbol	Entrez Gene Name	Affymetrix	Fold Change	Location	Function	Entrez Gene ID for Human	Entrez Gene ID for Rat
ADCK3	aarF domain containing kinase 3	1372536_at	-1.741	Cytoplasm	kinase	56997	360887
BAX	BCL2-associated X protein	1369122_at	2.034	Cytoplasm	other	581	24887
BBC3	BCL2 binding component 3	1382993_at	1.922	Cytoplasm	other	27113	317673
C12orf5	chromosome 12 open reading frame 5	1374777_at	1.958	unknown	enzyme	57103	502894
CCNG1	cyclin G1	1367764_at	4.342	Nucleus	other	900	25405
CDKN1A	cyclin-dependent kinase inhibitor 1A (p21, Cip1)	1387391_at	2.131	Nucleus	other	1026	114851
CHEK1	checkpoint kinase 1	1387062_a_at	1.64	Nucleus	kinase	1111	140583
GADD45A	growth arrest and DNA-damage-inducible, alpha	1368947_at	1.707	Nucleus	other	1647	25112
GNL3	guanine nucleotide binding protein-like 3 (nucleolar)	1388953_at	1.88	Nucleus	other	26354	290556
HIPK2	homeodomain interacting protein kinase 2	1373497_at	-1.904	Nucleus	kinase	28996	362342
JUN	jun proto-oncogene	1374404_at	1.51	Nucleus	transcription regulator	3725	24516
KAT2B	K(lysine) acetyltransferase 2B	1375205_at	-1.829	Nucleus	transcription regulator	8850	301164
MDM2	Mdm2, p53 E3 ubiquitin protein ligase homolog (mouse)	1383485_at	3.435	Nucleus	transcription regulator	4193	314856
PCNA	proliferating cell nuclear antigen	1367671_at	1.723	Nucleus	enzyme	5111	25737
PIDD	p53-induced death domain protein	1398569_at	1.588	Cytoplasm	other	55367	293625
RB1	retinoblastoma 1	1388185_at	-1.499	Nucleus	transcription regulator	5925	24708
SFN	stratifin	1374806_at	1.956	Cytoplasm	other	2810	313017
STAG1	stromal antigen 1	1389226_at	-2.758	Nucleus	other	10274	315958
TP53INP1	tumor protein p53 inducible nuclear protein 1	1391826_at	1.519	Nucleus	other	94241	297822
